# A Combination of Independent Transcriptional Regulators Shapes Bacterial Virulence Gene Expression during Infection

**DOI:** 10.1371/journal.ppat.1000817

**Published:** 2010-03-19

**Authors:** Samuel A. Shelburne, Randall J. Olsen, Bryce Suber, Pranoti Sahasrabhojane, Paul Sumby, Richard G. Brennan, James M. Musser

**Affiliations:** 1 Department of Infectious Diseases, MD Anderson Cancer Center, Houston, Texas, United States of America; 2 Center for Molecular and Translational Human Infectious Diseases Research, The Methodist Hospital Research Institute, and Department of Pathology, The Methodist Hospital, Houston, Texas, United States of America; 3 Department of Biochemistry and Molecular Biology, MD Anderson Cancer Center, Houston, Texas, United States of America; Johns Hopkins School of Medicine, United States of America

## Abstract

Transcriptional regulatory networks are fundamental to how microbes alter gene expression in response to environmental stimuli, thereby playing a critical role in bacterial pathogenesis. However, understanding how bacterial transcriptional regulatory networks function during host-pathogen interaction is limited. Recent studies in group A *Streptococcus* (GAS) suggested that the transcriptional regulator catabolite control protein A (CcpA) influences many of the same genes as the control of virulence (CovRS) two-component gene regulatory system. To provide new information about the CcpA and CovRS networks, we compared the CcpA and CovR transcriptomes in a serotype M1 GAS strain. The transcript levels of several of the same genes encoding virulence factors and proteins involved in basic metabolic processes were affected in both Δ*ccpA* and Δ*covR* isogenic mutant strains. Recombinant CcpA and CovR bound with high-affinity to the promoter regions of several co-regulated genes, including those encoding proteins involved in carbohydrate and amino acid metabolism. Compared to the wild-type parental strain, Δ*ccpA* and Δ*covR*Δ*ccpA* isogenic mutant strains were significantly less virulent in a mouse myositis model. Inactivation of CcpA and CovR alone and in combination led to significant alterations in the transcript levels of several key GAS virulence factor encoding genes during infection. Importantly, the transcript level alterations in the Δ*ccpA* and Δ*covR*Δ*ccpA* isogenic mutant strains observed during infection were distinct from those occurring during growth in laboratory medium. These data provide new knowledge regarding the molecular mechanisms by which pathogenic bacteria respond to environmental signals to regulate virulence factor production and basic metabolic processes during infection.

## Author Summary

Group A *Streptococcus* (GAS) causes diverse infections in humans ranging from pharyngitis (strep throat) to necrotizing fasciitis (the flesh-eating disease). It is well known that GAS secretes a broad array of virulence factors that are critical to its ability to cause human infections, but how GAS coordinates virulence factor production during infection is poorly understood. We discovered that two GAS proteins, catabolite control protein A (CcpA) and control of virulence regulator (CovR), regulate production of many of the same virulence factor encoding genes, indicating that GAS uses these two regulatory proteins to modulate virulence factor production in response to environmental stimuli. We determined that CcpA and CovR are able to bind to DNA from co-regulated genes, indicating that the proteins control gene expression by directly interacting with DNA. Using a mouse model of muscle infection, we found that CcpA and CovR, alone and in combination, are critical to the ability of GAS to regulate expression of virulence factor encoding genes during infection. These findings increase understanding regarding the regulatory mechanisms critical to the ability of bacterial pathogens to cause infection.

## Introduction

It has long been recognized that the gene expression profile of bacterial pathogens differs significantly during infection compared to the laboratory environment [1]. For example, a recent study of *Listeria monocytogenes* found that more than 1,000 genes were differentially expressed when comparing bacteria grown in a standard laboratory medium with the same bacteria recovered from mouse intestine [2]. Genes encoding bacterial virulence factors are often upregulated during infection, but the molecular mechanisms governing virulence gene expression in the host are only beginning to be understood [3],[4]. Specifically, there is a dearth of information available regarding how transcriptional regulatory networks function in response to host environmental stimuli to determine virulence factor production [5].

Group A *Streptococcus* (GAS) causes infections in humans ranging from uncomplicated pharyngeal and skin infections to necrotizing fasciitis and toxic shock-like syndrome [6]. The ability of GAS to cause infection in diverse human niches indicates that GAS has evolved precise mechanisms to alter gene expression depending on the distinct challenges posed by particular disease sites [7],[8],[9]. Unlike some other pathogenic bacteria, GAS does not appear to regulate virulence factor production by alternative sigma factors that can associate with core RNA polymerase [10],[11]. Thus, gene expression in GAS is heavily dependent on transcriptional regulatory networks [12]. GAS encodes two main types of regulatory proteins, namely stand-alone regulators and two-component gene regulatory systems (TCS) [12],[13]. The interaction of stand-alone regulators with DNA changes with alterations in intracellular conditions, such as the presence of an inducing substrate [12]. In contrast, TCS typically consist of a membrane-embedded sensor kinase that controls the phosphorylation state of a cognate cytoplasmic response regulator in response to environmental stimuli [14]. The phosphorylation state of the regulator influences its DNA binding activity thereby affecting gene expression [15]. Although interaction between TCS and stand-alone regulators is clearly critical to bacterial pathogenesis, information regarding how independent bacterial regulators coordinate gene expression during infection is lacking.

The best studied GAS transcription factor is the control of virulence regulator (CovR), also known as CsrR for capsule synthesis regulator [7],[16],[17],[18],[19]. CovR is the response regulator of the CovRS TCS [7]. Studies done over the past decade have revealed several intriguing aspects of the CovRS TCS. First, in contrast to most TCS regulators, CovR mainly serves to negatively affect gene expression, including repressing numerous genes encoding key virulence factors [7],[19],[20]. Thus, GAS strains in which CovR has been genetically inactivated are hypervirulent for mice [17],[20],[21],[22]. Second, CovR appears to be able to function independently of CovS as GAS strains with isogenic CovR or CovS mutations have different phenotypes [16],[23]. Third, phosphorylation of CovR generally leads to an increase in affinity for target DNA *in vitro* although the degree to which phosphorylation increases CovR binding affinity differs for various promoters [24],[25]. Finally, spontaneous mutations in *covR* or *covS* have been identified in strains from animals with experimental GAS infections and humans with invasive infections indicating that mutations in *covRS* provide an advantage in select *in vivo* environments [21],[26],[27],[28].

Although most CovRS-related research has focused on virulence factor regulation, the CovR regulon also includes many genes involved in carbohydrate catabolism and nitrogen utilization [20],[29]. Inasmuch as CovR regulates genes involved in virulence and basic metabolic processes, CovR appears to have a similar transcriptional profile to catabolite control protein A (CcpA) [30],[31]. CcpA is a stand-alone, global regulatory protein critical to selective carbon source utilization and nitrogen metabolism that has recently been shown to contribute to virulence in GAS and other Gram-positive pathogens [30],[31],[32],[33],[34],[35]. Studies in *Bacillus* species and other non-pathogenic Gram-positive bacteria have found that the binding of CcpA to DNA catabolite response element (*cre*) sites is significantly enhanced by interaction of CcpA with histidine containing phosphoprotein (HPr) phosphorylated at serine residue 46 (HPr-Ser46-P) [36],[37]. The HPr phosphorylation state is determined by the action of HPr kinase/phosphorylase (HPrK/P), a bifunctional enzyme whose activity, in turn, is affected by the intracellular concentration of carbohydrate catabolism products [38]. Orthologues of CcpA, HPr, and HPrK/P from *Bacillus* species are present in all fully sequenced Gram-positive pathogens. However, definitive evidence that the CcpA-(HPr-Ser46-P)-HPrK/P axis functions in a similar fashion in pathogenic Gram-positive organisms to that observed in *Bacillus* species is lacking, and there are limited data available regarding how CcpA influences gene expression during infection [39].

Analysis of the CcpA transcriptome in GAS led to the understanding that many genes influenced by CcpA also are part of the CovR transcriptome [30],[31]. Moreover, bioinformatic analysis suggests that CcpA and CovR DNA binding sites can be proximally located [30],[31]. Similarly, studies of CcpA and CovR orthologues in *Staphylococcus aureus* (in which the CovR orthologue is known as ArlR for autolysis related locus) and *Streptococcus mutans* have suggested significant overlap in genes regulated by these two proteins in non-GAS pathogenic bacteria [34],[40],[41],[42]. Therefore, we designed studies to test the hypothesis that the CcpA and CovRS systems co-regulate expression of a diverse array of GAS genes. Our results indicate that CcpA and CovR combine to shape the expression profile of GAS virulence factor-encoding genes and basic metabolic genes during infection. These data provide new insights into how transcriptional regulatory networks contribute to bacterial gene expression in the host environment and extend understanding of the close links between virulence and basic metabolic processes [43].

## Results

### The influence of CcpA on virulence factor production is dependent on GAS strain CovRS status

Pharyngeal GAS isolates usually have an intact (wild-type) CovRS system whereas GAS isolates recovered from invasive infections may have inactivating mutations in either CovR or CovS [21],[26],[27]. The two previous genome-wide studies on the effect of CcpA inactivation in GAS have both used the invasive clinical isolate serotype M1 strain MGAS5005, which encodes a truncated, functionally inactive CovS protein [16],[26],[30],[31]. To test the hypothesis that the effects of CcpA are dependent on CovRS status, we used non-polar insertional mutagenesis to create a Δ*ccpA* isogenic mutant (strain 2221Δ*ccpA*, Figure S1, Table 1) from the wild-type parental strain MGAS2221, a fully-sequenced serotype M1 strain that contains an intact CovRS TCS (Southern blot shown in Figure S1B) [26]. We genetically complemented the 2221Δ*ccpA* isogenic mutant strain using a CcpA-encoding plasmid that replicates in GAS to make strain comp2221Δ*ccpA*. The growth characteristic of the three strains in a standard laboratory medium (Todd-Hewitt broth with yeast extract, THY) were indistinguishable (Figure S2).

**Table 1:** Bacterial strains and plasmids used in this study.

Strain or plasmid	Description	Genotype	Reference
MGAS2221	Pharyngitis clinical isolate, serotype M1	Wild-type for *covRS*	[26]
2221Δ*ccpA*	Δ*ccpA* strain	MGAS2221 *ccpA::spc*	This study
comp2221Δ*ccpA*	Δ*ccpA* strain with CcpA complementation vector	Δ*ccpA*/*ccpA*+, Spc^r^ Cm^r^	This study
2221Δ*covR*	Δ*covR* strain	MGAS2221 *covR::kana*	[16]
2221Δ*covR*Δ*ccpA*	Δ*covR*Δ*ccpA* strain	2221Δ*covR ccpA*::*spc*	This study
MGAS5005	Invasive clinical isolate, serotype M1	Δ*covS* nt80	[67]
5005Δ*ccpA*	Δ*covS*Δc*cpA* strain	MGAS5005 *ccpA*::*spc*	[30]
comp5005Δ*ccpA*	Δ*covS*Δ*ccpA* strain with CcpA complementation vector	Δ*ccpA*/*ccpA*+, Spc^r^ Cm^r^	[30]
BL21-CcpA	*E. coli* BL21 producing recombinant GAS CcpA	BL21 pET-His2-CcpA Amp^r^	[30]
BL21-CovR	*E. coli* BL21 producing recombinant GAS CovR	BL21 pTXB1-CovR Amp^r^	This study
BL21-HPr	*E. coli* BL21 producing recombinant GAS HPr	BL21 pET-His2-HPr Amp^r^	This study
BL21-HPrK/P	*E. coli* BL21 producing recombinant GAS HPr kinase/phosphorylase	BL 21 pET21a-HPrK/P Amp^r^	This study
pSL60-1	Vector containing *aad9* gene	Spc^r^	[68]
pDC123	Chloramphenicol resistance vector	Cm^r^	[63]
pDCccpA	pDC123 with CcpA	Cm^r^	[30]
pET-His2-CcpA	pET-His2 plasmid with GAS CcpA gene	*ccpA^+^* Amp^r^	[30]
pTXB1-CovR	pTXB1 plasmid with GAS CovR gene	*covR^+^* Amp^r^	This study
pET-His2-HPr	pET-His2 plasmid with GAS HPr gene	*hpr^+^* Amp^r^	This study
pET21a-HPrK/P	pET21a plasmid with GAS HPrK/P gene	*hprK/P^+^* Amp^r^	This study

**Figure 1:**
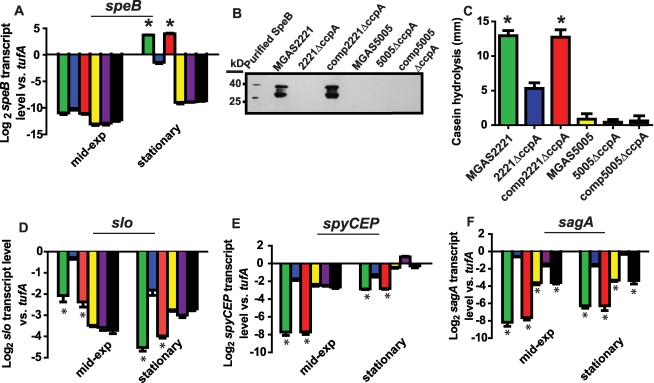
Influence of CcpA on GAS virulence factors is dependent on strain CovRS status. TaqMan real-time QRT-PCR for (A) *speB,* (D) *slo*, (E) *spyCEP*, and (F) *sagA* was performed for cells grown to indicated growth phase (mid-exp  =  mid-exponential). From left to right bars represent: MGAS2221 (CovRS wild-type, green); 2221Δ*ccpA* (blue); comp2221Δ*ccpA* (red); MGAS5005 (CovS mutant, yellow); 5005Δ*ccpA* (purple); and 5005compΔ*ccpA* (black). (B) Western immunoblot for SpeB in culture supernatants of indicated strains grown overnight in THY. The multiple bands observed represent zymogen (40 kb), intermediate, and mature (28 kb) SpeB forms reacting with anti-SpeB antibody [65]. (C) Casein hydrolysis of indicated strains as a measure of SpeB enzymatic activity. For all panels except (B) data graphed are mean +/− standard deviation of four biological replicates done on two separate occasions (i.e. eight samples). * indicates *P*<0.05 compared to CcpA inactivated strain as determined by ANOVA followed by Tukey's post-hoc test. *speB*, streptococcal pyrogenic exotoxin B; *slo*, streptolysin O; *spyCEP*, *Streptococcus pyogenes* cell envelope proteinase; *sagA*, streptolysin S.

We next determined the transcript level of the gene encoding streptococcal pyrogenic exotoxin B (SpeB) in the CovRS wild-type strains MGAS2221, 2221Δ*ccpA*, and comp2221Δ*ccpA* and in the CovS mutated strains MGAS5005, 5005Δ*ccpA*, and comp5005Δ*ccpA*. *speB* encodes a broad-spectrum, extracellular cysteine protease that is a key GAS virulence factor [44]. We analyzed *speB* because it is negatively regulated by CovR in serotype M1 strains, and a putative *cre* site is located approximately 100 bps into the *speB* open reading frame suggesting it could be directly regulated by CcpA [20] (Figure S3). During growth in THY, there was no significant difference in *speB* transcript level between strain MGAS5005 and its CcpA inactivated isogenic mutant strain (Figure 1A). In contrast, *speB* transcript level was significantly increased at the stationary phase of growth in strains MGAS2221 and comp2221Δ*ccpA* compared to strain 2221Δ*ccpA* (Figure 1A). Western immunoblot analysis (Figure 1B) and casein hydrolysis assays (Figure 1C) demonstrated that the observed transcript level variances translated into differences in immunoreactive SpeB in culture supernatants and in functional SpeB activity. These data demonstrate that, under the conditions tested, CcpA positively contributed to *speB* expression in the presence of a functional CovRS TCS but did not affect *speB* expression when CovS was inactive.

To determine if CovRS status affected the influence of CcpA on GAS virulence factors other than SpeB, we performed similar experiments to those described for SpeB using streptolysin O (Slo), an actively secreted cytotoxin [45] and *Streptococcus pyogenes* cell envelope proteinase (SpyCEP), an IL-8 degrading enzyme [46]. Putative *cre* sites are located in the *slo* promoter, which is co-transcribed with the *nga* gene (herein labeled *nga/slo cre*), and in the early *spyCEP* intragenic region (Figure S3), and CovR negatively regulates *nga/slo* and *spyCEP* in serotype M1 strains [16],[20]. As observed for SpeB, CcpA influenced the level of *slo* and *spyCEP* transcript in strain MGAS2221 but not strain MGAS5005 (Figure 1D, 1E). In contrast to our findings with *speB, slo*, and *spyCEP,* the transcript level of *sagA*, which encodes the first gene of the operon encoding the potent cytolysin streptolysin S (SLS), was significantly increased by CcpA inactivation in strain MGAS2221 as well as in strain MGAS5005 (Figure 1F). We and others have previously demonstrated binding of CcpA to the *sagA* promoter [30],[31]. Taken together, we conclude that the effect of CcpA on the expression of several key GAS virulence encoding genes varies depending on the functional CovRS status of the parental GAS strain.

### Recombinant CcpA-(HPr-Ser46-P) binds to DNA from multiple GAS CovR-regulated virulence factor encoding genes

**Figure 2:**
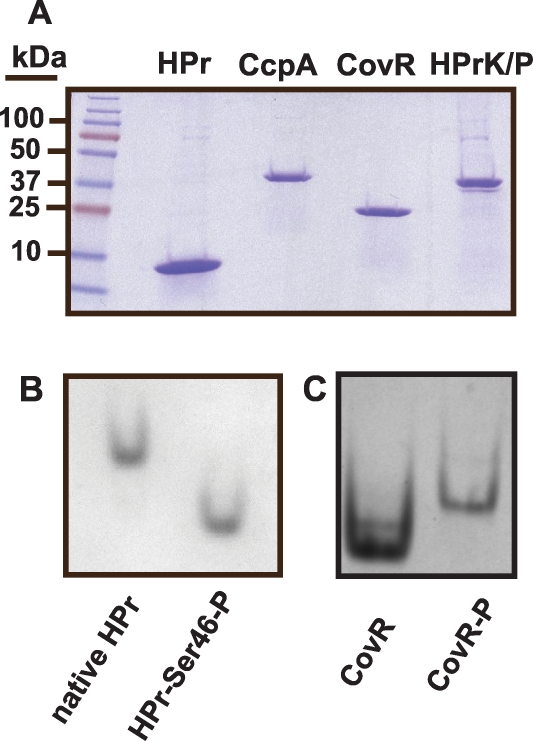
Recombinant GAS protein production and phosphorylation assays of recombinant GAS proteins. (A) SDS-PAGE analysis of recombinant GAS proteins. (B) Recombinant HPr was incubated with HPrK/P, purified, and analyzed under native conditions as described in Materials and Methods. Phosphorylation of HPr results in faster gel migration under these conditions. (C) Recombinant CovR was phosphorylated using acetyl phosphate as described in Materials and Methods. Unphosphorylated and phosphorylated CovR were run as described for HPr. Phosphorylation of CovR results in dimerization and thus slower migration through the gel under these conditions.

**Figure 3:**
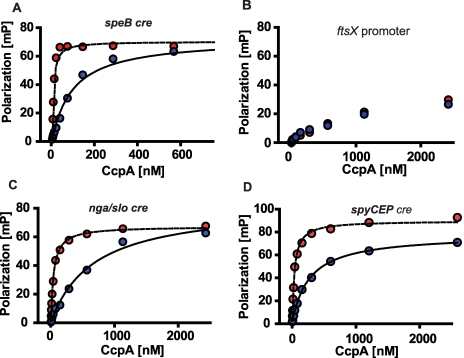
Recombinant CcpA-(HPr-Ser46-P) binds specifically to DNA from GAS genes encoding virulence factors. Fluorescence polarization based assay of (A) CcpA-*speB*, (C) CcpA-*nga/slo*, and (D) CcpA-*spyCEP cre* interaction. (B) shows CcpA interaction with DNA from non-CcpA regulated gene (*ftsX*, i.e. negative control). Binding assays were done with (red circles) and without (blue circles) 10 µm HPr-Ser46-P. Representative fluorescence polarization-based binding isotherms are shown of experiments done on four occasions. For (A), (C), and (D) lines indicate non-linear fit of binding data as described in Materials and Methods. *speB*, streptococcal pyrogenic exotoxin B; *ftsX*, cell division protein; *nga*, NAD glycohydrolase; *slo*, streptolysin O; *spyCEP*, *Streptococcus pyogenes* cell envelope proteinase.

We next tested the hypothesis that CcpA specifically binds to the putative *speB*, *nga/slo*, and *spyCEP cre* sites. For these studies, recombinant GAS CcpA, HPr, and HPrK/P were overexpressed and purified, and HPrK/P was used to produce HPr-Ser46-P as described in Materials and Methods (Figure 2). CcpA alone bound with high affinity to the *speB cre* site with a K_d_ of 100 nM (Figure 3A). As expected for a CcpA DNA binding site, the addition of HPr-Ser46-P significantly increased the affinity of CcpA for the *speB cre* site to a K_d_ of 5 nM (Figure 3A). Conversely, analysis of CcpA-(HPr-Ser46-P) binding to DNA from the *ftsX* promoter, a gene whose transcript level was not influenced by CcpA inactivation (e.g. a negative control), produced a non-specific DNA binding pattern (Figure 3B). Similarly, recombinant CcpA and the CcpA-(HPr-Ser46-P) complex also bound with high affinity to the *nga/slo* (K_d_ of 674 nM and 42 nM, respectively) and *spyCEP* (K_d_ of 257 nM and 33 nM, respectively) *cre* sites (Figure 3C and 3D). Together with previously published data indicating binding of CcpA to the *sagA* promoter, these data demonstrate that recombinant CcpA and the CcpA-(HPr-Ser46-P) complex bind with high affinity to *cre* site DNA from multiple virulence factor encoding genes that are directly regulated by CovR.

### CovR inactivation does not influence CcpA transcript levels under laboratory conditions

One potential mechanism by which the CovRS status of GAS could affect CcpA function is for CovR to regulate CcpA expression. The consensus GAS CovR binding sequence is ATTARA, where R  =  A or G [7]. There is one ATTARA motif in the vicinity of the CcpA promoter. We tested whether CovR influenced CcpA transcript level by determining *ccpA* transcript level in strain 2221Δ*covR* [16]. During growth in THY there was no significant difference in *ccpA* transcript level between strains MGAS2221, 2221Δ*covR*, or MGAS5005 (the *covS* mutant strain) (Figure S4). There are no putative *cre* sites in the CovR promoter region. Thus, not surprisingly, there was no significant difference in *covR* transcript level between strains MGAS2221 and 2221Δ*ccpA* or strains MGAS5005 and 5005Δ*ccpA* (Figure S4). These data suggest that although CovR and CcpA each influence the expression of several of the same key GAS virulence factors, CovR and CcpA do not influence the transcript level of the other regulator under the conditions studied.

### Identification of significant overlap between the CcpA and CovR transcriptomes

To better understand the relationship between CcpA and the CovRS TCS, we created a CcpA/CovR double mutant strain by genetically inactivating *ccpA* in strain 2221Δ*covR*, resulting in strain 2221Δ*covR*Δ*ccpA* (Table 1, Figure S1). There was no significant difference in the doubling time or final density of organisms grown in THY between strain 2221Δ*covR*Δ*ccpA* and strains MGAS2221, 2221Δ*ccpA*, and 2221Δ*covR* (Figure S2C, Table S1). Although previous experiments suggested that CcpA and CovR influence expression of many of the same GAS genes, the CcpA and CovR transcriptomes have not been directly compared [20],[29],[30],[31]. Therefore, we next tested the hypothesis that inactivation of CcpA and CovR have similar effects on the GAS transcriptome by performing expression microarray analysis of strains MGAS2221, 2221Δ*ccpA*, 2221Δ*covR*, and 2221Δ*covR*Δ*ccpA* during the mid-exponential and stationary phases of growth in THY (see Figure S2C for RNA isolation points and Figure S5 for principal component analyses of the microarray data). Quadruplicate replicates were performed for each strain at each time point. At both the mid-exponential and stationary growth points, the percent of total ORFs with a significant difference in transcript levels compared to the wild-type strain was approximately 10% for strain 2221Δ*ccpA*, 15% for strain 2221Δ*covR*, and 20% for strain 2221Δ*covR*Δ*ccpA* (Tables S2, S3 and S4).

**Figure 4:**
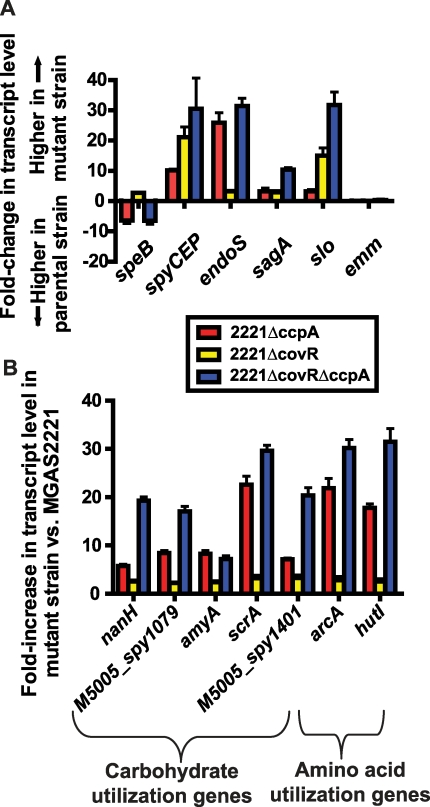
Identification of significant overlap between the CcpA and CovR transcriptomes. Differences in gene transcript levels measured by expression microarray analysis for select GAS virulence factor encoding genes (A) and genes involved in carbohydrate and amino acid utilization (B). Data graphed are mean +/− standard deviation for quadruplicate samples for strains as indicated in the insert box. All transcript levels are significantly different compared to strain MGAS2221 except for the *emm* gene which is shown for reference purposes. A significant difference was defined as at least two-fold difference in median gene transcript level and *P*<0.05 for the indicated isogenic mutant strain compared to the parental wild-type strain MGAS2221. *speB*, streptococcal pyrogenic exotoxin B; *spyCEP*, *Streptococcus pyogenes* cell envelope proteinase; *endoS*, endoglycosidase S; *sagA*, streptolysin S; *slo*, streptolysin O; *emm*, M protein; *nanH*, acetylneuraminate lyase*; amyA*, cyclomaltodextrin glucanotransferase; *scrA*, sucrose transport enzyme; *arcA*, arginine deiminase; *hutI*, imidazolonepropionase.

We discovered significant overlap in the CcpA and CovR transcriptomes, primarily in genes encoding proteins known to be or putatively involved in virulence and in the transport and metabolism of carbohydrates and amino acids (Table 2). Genes encoding proteins known to be or putatively involved in virulence that were affected by both CcpA and CovR included *speB*, *spyCEP*, *endoS,* and the operons encoding SLS and Slo (Figure 4A). EndoS (endoglycosidase S) cleaves a glycan side chain from human immunoglobulin G [47]. The effects of CcpA and CovR on *sagA, slo*, *spyCEP*, and *speB* transcript level were confirmed by QRT-PCR (Figure S6). A casein hydrolysis assay confirmed that strain-to-strain differences in *speB* transcript levels resulted in altered functional SpeB activity (Figure S6E). All putative virulence factor genes that were affected by CcpA inactivation were also affected by CovR inactivation. However, the CovR transcriptome included several GAS virulence factors not influenced by CcpA such as the genes encoding streptokinase and the immunoglobulin cleaving protease Mac-1/IdeS (Tables S2 and S3).

**Table 2:** Selected genes/operons co-regulated by CcpA and CovR under laboratory conditions.

Gene category	M5005 spy number	Gene name	Function of encoded protein(s)	2221Δ*ccpA*	2221Δ*covR*	2221Δ*covR*Δ*ccpA*
Virulence factors						
	0139	*nga*	NAD glycohydrolase	3.6^a^	12.1	31.2
	0141	*slo*	Pore forming cytoxin	2.7	12.3	25.3
	0341	*spyCEP*	IL-8 degrading protease	8.3	17.3	25.5
	0562	*sagA*	Secreted cytotoxin	2.7	2.3	8.4
	1415	*sdaD2*	DNase	2.3	2.7	2.2
	1540	*endoS*	Immunoglobulin modifying protein	21.3	2.6	26.6
	1714	*fba*	Fibronectin-binding protein	3.2	4.1	5.0
	1735	*speB*	Secreted cysteine protease	−5.2	2.2	−5.2
Carbohydrate utilization						
	0212-8^b^		Sialic acid production and catabolism	13.7	4.8	37.0
	1079-83		Cellobiose transport	8.4	3.1	12.7
	1062-7		Cyclodextrin transport and catabolism	7.9	2.4	8.8
	1538-43		Sucrose transport and catabolism	13.3	2.3	19.0
	1632-8		Lactose transport and catabolism	6.32	2.9	11.3
Amino acid utilization						
	1271-5		Arginine catabolism	22.0	2.7	29.4
	1770-8		Histidine catabolism	5.73	2.3	11.3

a Numbers show mean-fold-change in transcript level between wild-type (strain MGAS2221) and indicated isogenic mutant strain with positive numbers indicating higher transcript level in isogenic mutant strain compared to wild-type.

b Numbers listed for carbohydrate and amino acid utilization operons indicate mean-fold-change in transcript level for all genes in the indicated operon for isogenic mutant strain compared to wild-type.

In addition to finding that CovR and CcpA influenced expression of several of the same virulence factor genes, we also observed alterations in the transcript levels of many of the same metabolic gene operons for the 2221Δ*ccpA*, 2221Δ*covR*, and 2221Δ*covR*Δ*ccpA* strains compared to strain MGAS2221 (Table 2, Figure 4B). These included operons known to be or putatively involved in carbohydrate metabolism and operons encoding proteins in the arginine deiminase and histidine degradation pathways. For metabolic operons, CcpA inactivation tended to result in more significant alteration in transcript levels compared to CovR inactivation (e.g. the differences in the transcript of *arcA*, the first gene in the arginine deminase operon, were ∼20-fold between wild-type and strain 2221Δ*ccpA* vs. ∼3-fold between wild-type and strain 2221Δ*covR*, Figure 4B). Moreover, CcpA inactivation affected several metabolic operons that were not affected by CovR inactivation (e.g. the putative mannose/fructose transport system operon). Taken together, we conclude that under laboratory conditions CcpA and CovR influence expression of many of the same genes with CovR having a greater impact on virulence gene expression whereas CcpA has a greater influence on the expression of genes involved in basic metabolic processes.

### Recombinant GAS CovR and CcpA-(HPr-Ser46-P) bind with high affinity to promoter DNA of the same metabolic genes

**Figure 5:**
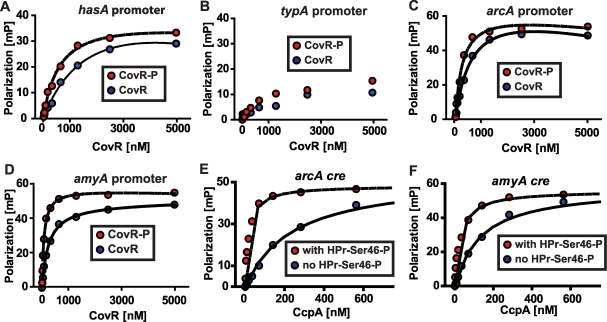
Recombinant CovR and CcpA bind to DNA from promoter regions of the same genes. (A-D) Representative fluorescence polarization-based isotherms of unphosphorylated (blue circles) and phosphorylated CovR (red circles, CovR-P) binding to 1 nM of fluorescein-labeled DNA. Millipolarization units (mP) are plotted against the CovR concentration. (A) Recombinant CovR interaction with DNA from the *hasA* promoter (positive control). (B) CovR interaction with DNA from promoter of the non-CovR regulated gene *typA* (i.e. negative control). For (B) note linear increase in MP values with increasing CovR concentration indicating low affinity protein-DNA interaction. (C) Recombinant CovR interacting with DNA from the amino acid utilization gene *arcA*. (D) CovR interaction with DNA from the carbohydrate utilization gene *amyA*. CcpA interaction with *arcA cre* (E) and *amyA cre* (F) is shown with (red circles) and without (blue circles) the presence of 10 µm HPr-Ser46-P. For all panels experiments were done on at least three occasions, and lines indicate fit of binding data as described in Materials and Methods. *hasA*, hyaluronan synthase; *typA*, GTP-binding protein; *arcA*, arginine deminase; *amyA*, cyclomaltodextrin glucanotransferase.

The significant overlap between the CovR and CcpA transcriptomes led us to hypothesize that these two proteins bind to the promoter DNA of several of the same genes. Recombinant CovR was overexpressed, purified, and phosphorylated as described in Materials and Methods (Figure 2). This purified CovR lacks non-native residues, can be readily concentrated, and remains soluble at high concentrations. We used fluorescence polarization to study protein-DNA interaction because this method is equilibrium-based and done in solution, thereby approximating the *in vivo* environment (for details on fluorescence polarization see the Materials and Methods section) [48]. Fluorescence polarization has not been previously used to study CovR-DNA interaction. Therefore, we first tested the binding of recombinant CovR to the *hasA* promoter for which CovR-DNA interaction has been well characterized [18],[25],[49]. Recombinant CovR bound to the *hasA* promoter DNA with an approximately 4-fold increase in affinity when CovR was phosphorylated (K_d_ decreased from 2200 nM to 640 nM, Figure 5A), which is consistent with previous reports regarding the effects of phosphorylation on CovR-*hasA* promoter binding [18],[25]. Analysis of recombinant CovR with labeled DNA from the promoter region of the non-CovR regulated gene *typA* (i.e. a negative control) produced low polarization changes and linear binding consistent with low affinity, non-specific DNA binding (Figure 5B). These data indicated that we could reliably use fluorescence polarization to investigate CovR-DNA binding.

In terms of genes involved in basic metabolic processes, the transcriptome data demonstrated altered transcript levels of *arcA*, which encodes a protein involved in arginine utilization, and *amyA*, which encodes an actively secreted carbohydrate-degrading protein, in strains 2221Δ*ccpA* and 2221Δ*covR* compared to wild-type (Figure 4B). Thus, we next tested the hypothesis that recombinant CovR and recombinant CcpA-(HPr-Ser46-P) bind with high affinity to DNA from the *arcA* and *amyA* promoters. Recombinant CovR bound specifically and with reasonably high affinity to the promoter regions of the *arcA* (K_d_ of 637 nM and 230 nM for unphosphorylated and phosphorylated CovR respectively, Figure 5C) and *amyA* genes (K_d_ of 245 nM and 77 nM for unphosphorylated and phosphorylated CovR respectively, Figure 5D). Similarly, recombinant CcpA bound with high affinity to putative *cre* sites from the *arcA* (K_d_ of 219 and 18 nM without and with HPr-Ser46-P respectively) and *amyA* (K_d_ of 160 and 32 without and with HPr-Ser46-P respectively) promoters (Figure 5E and 5F). Together with previous data regarding the binding of CovR and CcpA-(HPr-Ser46-P) to DNA from virulence factor encoding genes (Figure 2) [18], these data provide a mechanism for the extensive overlap observed in the CcpA-CovR transcriptome data.

### CcpA and CovR contribute to the alteration in GAS gene expression observed during growth in human saliva

**Figure 6:**
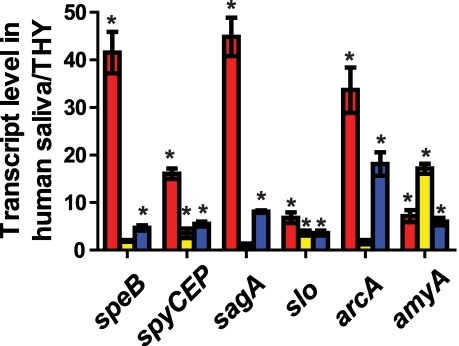
CcpA and CovR contribute to GAS gene expression during growth in human saliva. GAS strains (red bars  =  strain MGAS2221, yellow bars  =  strain 2221Δ*ccpA*, and blue bars  =  strain 2221Δ*covR*) were grown to late exponential phase in standard laboratory medium (THY) or human saliva and transcript level of indicated genes were assessed using TaqMan QRT-PCR. Quadruplicate replicates of each strains were assessed on two separate occasions for a total of eight replicates. * indicates *P*<0.05 for difference in mean transcript level of the indicated gene in human saliva compared with THY as determined by Student's *t-*test assuming unequal variances using Bonferroni's adjustment for multiple comparisons. *speB*, streptococcal pyrogenic exotoxin B; *spyCEP*, *Streptococcus pyogenes* cell envelope proteinase; *sagA*, streptolysin S; *slo*, streptolysin O; *arcA*, arginine deiminase; *amyA*, cyclomaltodextrin glucanotransferase.

We have previously demonstrated that GAS markedly alters its transcriptome during interaction with human saliva compared with growth in a laboratory medium [30],[50]. Given that the CcpA-(HPr-Ser46-P) complex and CovRS TCS are known to be part of the process by which GAS responds to changes in the environment [30],[51], we next tested the hypothesis that CcpA and CovR contribute to how GAS modifies gene expression in response to interaction with human saliva. We determined the transcript levels of six genes known to be directly regulated by CcpA and CovR, four that encode virulence factors and one each encoding a carbohydrate utilization and amino acid utilization protein. For the parental wild-type strain MGAS2221, the transcript level of *speB*, *spyCEP*, *slo*, *sagA*, *arcA*, and *amyA* was significantly increased during growth in human saliva compared to THY (Figure 6). In contrast, the transcript level of *speB*, *sagA*, and *arcA* were not significantly different in strain 2221Δ*ccpA* between growth in human saliva and laboratory medium indicating that CcpA was needed for the altered gene expression pattern observed in the wild-type strain in human saliva compared with THY (Figure 6). Although the transcript level of *spyCEP* and *slo* were increased in strain 2221Δ*ccpA* during growth in human saliva, the increase in gene transcript level between the two conditions was significantly less than that observed for strain MGAS2221. Similarly, the transcript level of each of the six genes tested was increased in human saliva to a lesser degree in strain 2221Δ*covR* compared to wild-type (Figure 6). These data indicate that, for the genes tested, CcpA and CovR participate in the remodeling of GAS gene expression in response to human saliva.

### CcpA and CovR affect GAS virulence and contribute to the GAS gene expression profile during infection

**Figure 7:**
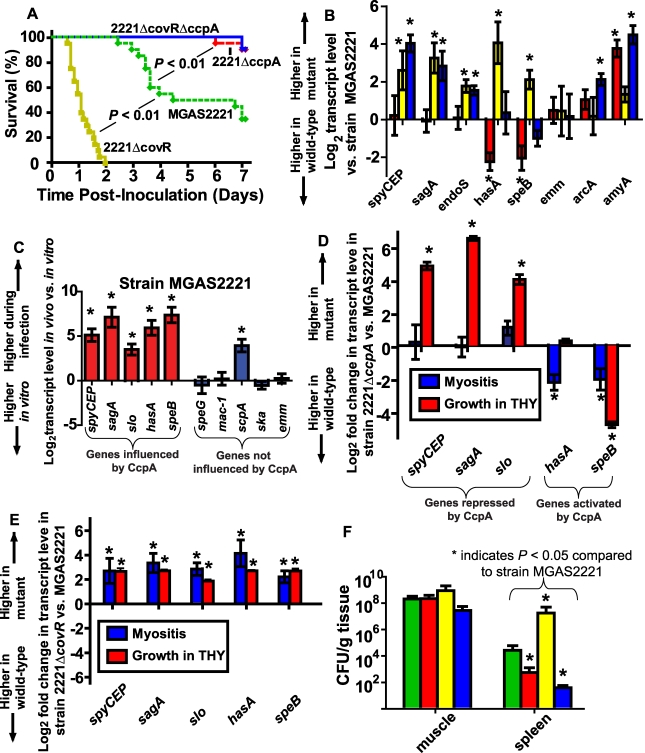
CcpA inactivation significantly decreases GAS virulence and affects GAS virulence gene expression during infection. (A) 20 outbred CD-1 mice per GAS strain were infected intramuscularly with 2.5×10^7^ CFU of indicated strains. Data graphed are survival over time with *P* values derived from Kaplan-Meier survival analysis. (B) Transcript levels of select GAS genes were determined in mouse muscle using QRT-PCR. From left to right bars represent 2221Δ*ccpA* (red); 2221Δ*covR* (yellow); and 2221Δ*covR*Δ*ccpA* (blue). (C) Comparison of gene transcripts in strain MGAS2221 during infection compared with late exponential growth phase in THY. Comparison of gene transcripts between (D) strains MGAS2221 and 2221Δ*ccpA* and (E) strains MGAS2221 and 2221Δ*covR* during infection (blue bars) and during late exponential growth phase in THY (red bars). For (B-E) data graphed are mean +/− standard deviation of four biologic replicates analyzed in duplicate. (F) Bacterial density in muscle (lesion site) and spleen (disseminated infection site). Bars are as for panel A except that green bars represent strain MGAS2221. For all panels, * indicates *P*<0.05 for indicated comparison. *spyCEP*, *Streptococcus pyogenes* cell envelope proteinase; *sagA*, streptolysin S; *slo*, streptolysin O; *endoS*, endoglycosidase S; *hasA,* hyaluronan synthase; *speB*, streptococcal pyrogenic exotoxin B; *emm*, M protein; *arcA*, arginine deiminase; *amyA*, cyclomaltodextrin glucanotransferase; *speG*, streptococcal pyrogenic exotoxin G; *mac-1*, IgG degrading protease; *scpA*, streptococcal C5a peptidase; *ska*, streptokinase.

Thus far in the work, our data had shown significant overlap in the CcpA and CovR transcriptomes, that CcpA and CovR bind to DNA from several of the same genes, and the CcpA and CovR are key to how GAS remodels its gene expression profile during interaction with human saliva. To study the *in vivo* relevance of how CcpA and CovR together contribute to GAS pathogenesis, we compared the virulence of strain MGAS2221 to mutant strains 2221Δ*ccpA*, 2221Δ*covR*, and 2221Δ*covR*Δ*ccpA* using a mouse myositis model [52]. As expected for a negative virulence-gene regulator, CovR inactivation significantly decreased mouse survival compared to wild-type infected animals (Figure 7A, *P*<0.01). Conversely, mice infected with strain MGAS2221 had a significantly increased mortality rate compared to mice infected with mutant strain 2221Δ*ccpA* or mutant strain 2221Δ*covR*Δ*ccpA* (Figure 7A). We analyzed RNA recovered from GAS in mouse muscle to correlate the GAS gene expression profile with the mortality data. The elevated transcript level of virulence factor encoding genes in strain 2221Δ*covR* compared to strain MGAS2221 is consistent with the hypervirulent phenotype of the CovR mutant strain (Figure 7B). However, in contrast to what was observed during growth in THY, there was no significant difference in *spyCEP, sagA*, and *slo* transcript level between wild-type and strain 2221Δ*ccpA* during infection (Figure 7B). In terms of metabolic genes, there was no significant difference in *arcA* transcript level during infection between strain 2221Δ*ccpA* and its parental, wild-type strains whereas *amyA* transcript level was significantly increased in the CcpA-inactivated strain (Figure 7B). Finally, compared to strain MGAS2221, *speB* and *hasA* transcript levels were significantly decreased in the CcpA-inactivated strains in mouse muscle (Figure 7B), providing a potential explanation for the diminished virulence of the CcpA inactivated strains.

To gain further insight into the molecular mechanisms underlying GAS gene expression during invasive infection, we next compared select virulence gene transcript levels in strain MGAS2221 during infection with those observed during growth in THY. We determined the relative transcript levels of five virulence factor encoding genes known to be influenced by CcpA and five genes not known to be influenced by CcpA (Figure 7C). The transcript level of all of the CcpA-influenced genes were increased in strain MGAS2221 during infection compared to growth in THY whereas the transcript level of only one of the non-CcpA-influenced genes was increased during infection (Figure 7C). These data suggest that CcpA may be either repressing gene transcript levels during growth in THY or activating gene expression during infection. By comparing gene transcript levels in strain MGAS2221 and 2221Δ*ccpA*, we found that CcpA repressed *spyCEP*, *sagA*, and *slo* during growth in laboratory medium but not during infection (Figure 7D). Conversely, the transcript level pattern of *hasA* and *speB* indicated that CcpA was activating these genes during infection. Comparison of transcript levels during infection versus growth in THY for strain MGAS2221 and 2221Δ*covR* demonstrated that CovR inactivation resulted in relatively similar effects on GAS gene expression for the two conditions (Figure 7E). Taken together, we conclude that CcpA and CovR contribute to the virulence gene expression profiles of GAS during infection but that the effect of CcpA on GAS gene expression differs significantly depending on the studied environment.

### CcpA inactivation decreases emergence of GAS strains with spontaneous covRS mutations

The decreased virulence of the CcpA-inactivated strains in the mouse myositis model suggested there was either a decreased intensity of the local infectious process for the CcpA-inactivated strains or that the CcpA-deficient strains had a diminished rate of bacterial dissemination from the primary infection site. To distinguish between these two possibilities, we determined the number of viable GAS CFUs present in mouse limbs (local infection site) and mouse spleens (disseminated infection site) 48 hrs after infection. We observed no significant difference among the four strains in the number of viable GAS CFUs present in the infected limbs at 48 hrs post-inoculation (Figure 7F). However, the wild-type and 2221Δ*covR* strains were recovered at significantly higher CFUs from mice spleens compared to the CcpA inactivated strains (Figure 7F).

Invasive GAS infection can be associated with spontaneous mutations in *covRS* [21],[26],[27],[28]. Therefore, one explanation for the decreased dissemination rate of the CcpA inactivated strains is that CcpA contributes to the emergence of GAS strains with *covRS* mutations. To test this hypothesis, we sequenced the *covRS* operon of GAS isolates from the spleens of mice infected with strain MGAS2221 and strain 2221Δ*ccpA*. *covRS* mutations were found in 17 of 24 isolates from mice infected with strain MGAS2221. In contrast, none of the 24 isolates from mice infected with strain 2221Δ*ccpA* had a *covRS* mutation (*P*<0.01 by Fisher's exact test). Isolates derived from strain MGAS2221 had missense mutations in CovR and nonsense mutations in CovS (see Table 3 for mutation details). These results indicate that interplay between the CcpA and CovRS systems contribute to the pathogenesis of invasive GAS infection.

**Table 3:** *covRS* mutations detected in GAS isolates from mice spleens following intramuscular inoculation with strain MGAS2221 and 2221Δ*ccpA*.

Parental strain	Gene	Mutation	Number of isolates	Effect of mutation
MGAS2221	*covS*	Deletion of GAAAA at bp 1209	6	Truncated CovS protein
MGAS2221	*covS*	Deletion of GAAAG at bp 1250	6	Truncated CovS protein
MGAS2221	*covR*	G286A	5	Missense mutation resulting in A96T amino acid change
2221Δ*ccpA*		No mutations identified		

## Discussion

It has long been recognized that bacteria react to environmental changes by altering expression of genes involved in basic metabolic processes. Indeed, early work on regulation of transcription demonstrated how activity of the *lac* operon varied in response to lactose concentration [53]. Similarly, for many years it has been recognized that bacterial virulence factor production changes in response to alterations in the environment [54],[55]. However, the molecular mechanisms underlying the control of bacterial virulence factor expression in particular environmental conditions, such as those encountered during human infection, are just beginning to be fully elucidated [51],[56]. Specifically, there has been limited investigation into how combinations of transcriptional regulators control gene expression during infection despite the clear importance of regulatory networks to microbial pathogenesis [5],[57]. The data generated herein demonstrate that the global metabolic gene regulator CcpA and the virulence factor regulator CovR act together to control expression of diverse GAS genes thereby contributing to the critical ability of GAS to remodel its transcriptome in response to distinct environmental cues.

**Figure 8:**
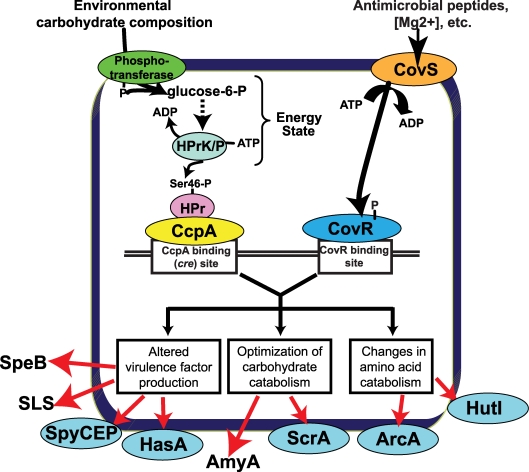
Model for how CcpA and CovR contribute to GAS gene expression profile. Transport of environmental carbohydrates through the phosphotransferase system mediates HPrK/P kinase/phosphorylase activity thereby affecting the formation of HPr-Ser46-P and thus the interaction of CcpA with various *cre* sites. Similarly, CovS responds to environmental stimuli such as low Mg^2+^ concentration or the presence of the human cathelicidin LL-37 [51],[69] by altering the phosphorylation of status of CovR thereby modifying CovR-DNA interaction. The binding (or lack thereof) of CcpA and CovR to GAS DNA results in altered expression of genes encoding virulence factors, carbohydrate catabolism proteins, and amino acid catabolism proteins critical to the pathogenesis of GAS infection. Proteins that are freely secreted into the extracellular environment (e.g. SpeB) are shown without a surrounding ellipse. SpeB, streptococcal pyrogenic exotoxin B; SLS, streptolysin S; SpyCEP, *Streptococcus pyogenes* cell envelope proteinase; HasA, hyaluronan synthase; AmyA, cyclomaltodextrin glucanotransferase; ScrA, sucrose transport enzyme; ArcA; arginine deiminase; HutI, imidazolonepropionase.

Examination of the gene expression and protein binding data generated during this investigation, along with data from previous studies [16],[51],[56],[58], allows us to generate a model for how CcpA and CovR participate in the alteration of GAS gene expression observed when GAS shifts from growth in laboratory medium to the host (Figure 8). The different environmental carbohydrate concentrations encountered during infection eventually result in a change in the ratio of kinase/phosphorylase activity of HPrK/P thereby altering the concentration of HPr-Ser46-P. The differences in HPr-Ser46-P levels will affect CcpA *cre* site interaction (as demonstrated by the protein binding data in Figures 3 and 5) thereby altering transcription of GAS virulence factor, carbohydrate catabolism, and amino acid catabolism encoding genes (Figure 8). At the same time, CovS responds to changes in environmental ion concentrations, such as Mg^2+^, and to the presence of innate immune peptides, by changing the phosphorylation status of CovR [51],[56],[58]. Phosphorylation/dephosphorylation of CovR alters its interaction with DNA, again changing the transcription of diverse GAS genes [16]. By having CcpA and CovR regulate expression of many of the same genes, the expression of a broad array of key GAS genes can be varied in response to an array of environmental signals. Appreciation of the potential complexity of the GAS CcpA-CovRS transcriptional network was broadened by a recent finding that CovS can either activate or repress CovR-mediated gene expression depending on the CovR target gene [16].

Our data demonstrate that the effect of CcpA on gene expression during host-pathogen interaction was significantly different from that observed during growth in standard laboratory medium and was dependent on whether the particular gene was activated or repressed by CcpA during growth in THY (Figure 7D). For example, during growth in THY CcpA repressed the transcript level of the key virulence factor encoding genes *sagA*, *spyCEP*, and *slo*. However, the transcript levels of these three genes were not increased in strain 2221Δ*ccpA* compared to strain MGAS2221 during infection Our protein-binding data indicate that binding of CcpA to *cre* sites in *sagA*, *spyCEP*, and *slo* at physiologic CcpA concentrations requires the presence of HPr-Ser46-P (Figure 3) [30],[59]. Thus, if GAS is experiencing a low HPr-Ser46-P state during infection, the absence of the CcpA-(HPr-Ser46-P) complex will likely release CcpA from *cre* site interaction thereby resulting in the increased *sagA*, *spyCEP*, and *slo* transcript levels observed in strain MGAS2221 in the host (Figure 7C). In contrast, the transcript levels of genes that were increased during infection in strain MGAS2221 and are activated by CcpA, such as *speB*, remained decreased in the CcpA inactivated strain compared to wild-type during infection (Figure 7D). Our findings that CcpA positively influences *speB* transcript level and directly binds to the *speB* regulatory region are in concert with a recent study examining the role of CcpA in GAS virulence gene expression (39). A possible explanation for these data is that recombinant CcpA binds to the *speB cre* site (Figure 3A) in the absence of HPr-Ser46-P with a K_d_ (∼100 nM) that is within the potential physiologic concentration of CcpA as determined in *Bacillus* species (20–250 nM) [59]. A previous study of CcpA in *B. subtilis* found that CcpA-mediated gene activation did not require the presence of HPr-Ser46-P [60]. Such a finding is consistent with our data demonstrating decreased *speB* transcript level in the CcpA-inactivated strain under conditions where HPr-Ser46-P levels are expected to be low or absent, such as growth in human saliva (Figure 6). Thus, the effect of CcpA on GAS gene expression *in vivo* is likely occurring by more than one mechanism. Our conclusions that CcpA affects gene expression during infection via multiple mechanisms and that CcpA-inactivation does not alter *sagA* transcript levels during host-pathogen interaction are similar to other recently published data despite the fact that a subcutaneous, rather than myositis, mouse model was used in that investigation [39].

Inactivation of CcpA markedly attenuated the virulence of the parental strain MGAS2221 whereas CovR inactivation significantly increased virulence (Figure 7A). A possible explanation for these observations can be derived from the expression microarray data which showed marked increases in the transcript levels of basic metabolic genes in the Δ*ccpA* isogenic mutant strain (Figure 4B). Thus, there are likely to be profound metabolic consequences of CcpA inactivation through inefficient production of proteins involved in carbon source acquisition and catabolism. Although there were also increases in metabolic gene transcript levels in the CovR isogenic mutant, the increases were smaller in comparison to strain 2221Δ*ccpA* (Figure 4B). This finding indicates that there may be less metabolic cost of CovR inactivation compared to CcpA inactivation. Inactivation of CcpA in the Δ*covR* background markedly decreased GAS virulence suggesting, in simplistic terms, that the metabolic consequences of CcpA inactivation outweighed the overexpression of virulence factors resulting from CovR inactivation. Such interplay between energy use and virulence factor production may have contributed to the lack of emergence of spontaneous mutations in the *covRS* operon in strain 2221Δ*ccpA* during murine soft-tissue infection.

Our discovery that significant overlap exists between the CovRS and CcpA transcriptional regulatory systems adds to understanding of the molecular mechanisms used by pathogenic human microbes to alter protein production in response to environmental changes. Interestingly, GAS CcpA and CovR binding sites can be proximal, indicating that the spatial organization of GAS promoters may allow for protein-protein interaction between the two regulators. The first description of cooperative DNA binding of a response regulator and an independent transcriptional regulator in a prokaryote was recently made in a study of developmental gene expression in *Myxococcus xanthus* [61]. We are currently investigating whether direct interaction between CcpA and CovR contributes to the ability of GAS to modulate global gene expression during infection. It has recently been demonstrated that targeting bacterial virulence factor regulation during infection can decrease infection severity [3],[62]. The data presented herein suggest that the CcpA-CovRS regulatory network is a potential target for the development of novel antimicrobials.

## Materials and Methods

### Ethics statement

Saliva was collected from human volunteers who gave their written informed consent under an MD Anderson Cancer Center Institutional Review Board approved protocol. Mouse experiments were performed according to protocols approved by the Methodist Hospital Research Institute Institutional Animal Care and Use Committee.

### Bacterial strains and culture media

The strains and plasmids used in this work are presented in Table 1, and primers used for isogenic mutant strain creation are listed in Table S5. The serotype M1 group A streptococcal (GAS) strains MGAS2221 and MGAS5005 are genetically representative of the serotype M1 clone responsible for most contemporary (post-1987) human infections, and both genomes have been sequenced [26]. Strain MGAS2221 and MGAS5005 are essentially genetically identical except for a truncation of the CovS protein in strain MGAS5005 [26]. Strain 5005Δ*ccpA* and comp5005Δ*ccpA* were described previously [30]. Strain 2221Δ*ccpA* and comp2221Δ*ccpA* were created using non-polar insertional mutagenesis and plasmid pDC*ccpA* in the same fashion as that described for CcpA isogenic mutant strains created from strain MGAS5005 [30]. pDC*ccpA* was created from plasmid pDC123, which is a low-copy number plasmid capable of replicating in Gram-positive organisms, by cloning the *ccpA* gene and promoter region from strain MGAS5005 into the multi-cloning site of pDC123 [63]. Selection for CcpA inactivation was via spectinomycin at 150 µg/mL and selection for the CcpA-complementing plasmid was done with chloramphenicol at 4 µg/mL. Strain 2221Δ*covR* was created as described [16]. Strain 2221Δ*covR*Δ*ccpA* was created by placing the spectinomycin resistance cassette in place of the CcpA gene in the 2221Δ*covR* strain with selection via spectinomycin. Strains were grown in a nutrient-rich medium (Todd-Hewitt broth with 0.2% yeast extract (THY)) at 37°C with 5% CO_2_.

### TaqMan transcript level analysis for bacteria grown in laboratory media

RNA was purified from four biological replicates on two separate occasions using an RNeasy Mini Kit (Qiagen). TaqMan real-time QRT-PCR (primers and probes listed Table S5) was performed with an Applied Biosystems 7500 system using the previously validated *tufA* gene as an internal control as described [64]. For QRT-PCR, a significant difference in transcript level was defined as having at least a 2-fold difference in the mean transcript level along with a *P* value of<0.05 for a two-sample *t-*test assuming unequal variance. QRT-PCR data are graphed in a log_2_ format to facilitate demonstration of either positive or negative regulation by CcpA and/or CovR.

### Expression microarray analysis

Samples for expression microarray analysis were performed in quadruplicate. A custom-made Affymetrix GeneChip® that contains 100% of the ORFs of strain MGAS2221 was used for expression microarray (transcriptome) studies as described [30]. To compare gene transcript levels between the wild-type and mutant strains, a two-sample *t*-test (unequal variance) was applied followed by a false discovery rate correction (*Q*<0.05) to account for multiple testing using Partek Genomics Suite version 6.4. Transcript levels were considered significantly different when the corrected *P* value was <0.05 and the mean difference was at least 2-fold. Principal component analysis was performed using the Partek Genomics Suite (Figure S5).

### Western immunoblot analysis

GAS strains were grown to indicated growth phases in THY. SDS-PAGE and immunoblotting were performed using specific anti-SpeB antibody [65].

### Casein hydrolysis assays

Functional SpeB protease activity was determined using casein hydrolysis as described [44].

### Purification and phosphorylation of recombinant GAS proteins

Recombinant GAS CcpA was purified to homogeneity from *Escherichia coli* as previously described (Figure 2A) [30]. Recombinant GAS HPr was obtained using the same cloning strategy as previously described for recombinant GAS CcpA (Figure 2A) [30]. To obtain functional HPrK/P, the GAS *hprK/P* gene was cloned from strain MGAS5005 into plasmid pET21a (Novagen) with primers designed such that no His tag was attached to the recombinant HPrK/P protein. An *E. coli* extract enriched for recombinant GAS HPrK/P was created by growing the BL21-HPrK/P cells in LB/ampicillin with 0.5 mM IPTG to an OD_600_ of 2.0. Cells were centrifuged and washed in 20 mM Tris-HCl pH 7.6 with 3 mM DTT, recentrifuged, and lysed via sonication in a buffer containing 0.2 mM Tris-HCl pH 7.6, 0.03 mM DTT, and 0.5 mM PMSF (a serine protease inhibitor). This lysate is enriched for HPrK/P (Figure 2A).

Phosphorylation of HPr at serine-46 was accomplished by incubating 500 µl of recombinant HPr for 20 mins at 37°C with 599 µl of HPrK/P extract in 5 mM ATP, 10 mM fructose-1,6-bisphosphate, 20 mM Tris-HCl pH 7.5, 1 mM DTT, and 5 mM MgCl_2_. To obtain purified HPr-Ser46-P, 100 µL of nickel resin (Qiagen) was added and the mixture was rotated for 1 hr at room temperature. The mixture was washed 4 times with 50 mM NaH_2_PO_4_ pH 8.0, 300 mM NaCl, and 20 mM imidazole, and HPr-Ser-46P was eluted with the same buffer except that the imidazole concentration was increased to 250 mM. The phosphorylation state of HPr was assayed by running the unphosphorylated and phosphorylated proteins on a native glycine gel (pH 10.4) (Figure 2B). Repeated analyses showed that phosphorylation of HPr was stable for at least one week.

To maintain CovR in its soluble form and to work with recombinant CovR protein that lacked a tag, we cloned the *covR* gene from MGAS5005 into plasmid pTXB1 (New England BioLabs) which resulted in a fusion protein with an intein tag and a chitin binding domain. Recombinant CovR was obtained following the manufacturer's instructions with release of the intein tag using DTT (Figure 2A). CovR was phosphorylated as described [25] with phosphorylation assessed by running unphosphorylated and phosphorylated CovR protein under non-denaturing conditions as described for HPr-Ser46-P (Figure 2C). Repeated assays showed a CovR phosphorlyation half-life of about 90 minutes, which is consistent with previous reports [66]. Thus, all experiments with phosphorylated CovR were performed immediately following phosphorylation completion. To remove all phosphorylation reagents, CovR was spun through protein desalting columns (Pierce) into freshly made DNA binding buffer (20 mM Tris, pH 7.5, 50 mM NaCl, 2 mM DTT, and 10 µg/mL of polydI:dC). All protein concentrations were assessed using the Bradford assay (Bio-Rad).

### DNA binding assays

DNA binding activity of CcpA and CovR was studied using a fluorescence polarization based assay. In brief, fluorescence polarization is an indirect measurement of the rotation of a molecule in solution that employs a fluorescently labeled molecule as a reporter. When two molecules interact, such as a protein binding to DNA that has been labeled with fluorescein, the intrinsic rotation of the DNA is slowed which can be observed as an increase in the polarization of the fluorescein. By titrating known amounts of protein into the binding solution, the equilibrium dissociation constant (K_d_) can be determined [48]. Fluorescence polarization was used as previously described to determine a series of CcpA-DNA binding constants with and without HPr-Ser46-P [30]. CovR binding affinities were measured using fluorescence polarization by titrating solutions of CovR (unphosphorylated or phosphorylated) into 200 µl of solution containing labeled DNA (1 nM) in 20 mM Tris, pH 7.5, 50 mM NaCl, and 2 mM DTT, and 10 µg/mL of polydI:dC. Polarization was measured at 25°C on a Beacon 2000 fluorescence polarization instrument (PanVera, Madison, WI). Data were analyzed assuming a 1:1 binding stoichiometry between functional protein unit and labeled DNA. Binding parameters were determined via non-linear regression using the equation Y  =  ((Bmax • X)/(K_d_ • X)) + NS • X where Bmax is the polarization value at maximum specific binding, K_d_ is the equilibrium dissociation constant and NS is the slope of non-specific binding. Goodness of fit (R^2^) values for each of the binding assays was >0.98.

### Mouse infection studies

Twenty female outbred CD-1 Swiss mice (Harlan-Sprague-Dawley) were injected intramuscularly in the right hind limb with 2.5×10^7^ GAS CFU using an established model of GAS intramuscular infection [52]. Comparison of mortality rates was performed by Kaplan-Meier survival analysis. Differences in mortality rates were considered significant for a *P* value of <0.05 after accounting for multiple comparisons. For quantitation of inoculation site CFUs, four mouse limbs per strain treatment group were homogenized in phosphate buffered saline and plated onto sheep blood agar, incubated for 24 hrs, and CFU counted. For quantitation of GAS dissemination, the same protocol was employed using mouse spleens instead of limbs. To compare rates of spontaneous *covRS* mutations, GAS colonies from spleens of mice that had been infected with strain MGAS2221 and 2221Δ*ccpA* were randomly selected for sequencing of the entire *covRS* operon. Six GAS colonies per mouse (4 mice were inoculated with each strain) were selected for sequencing for a total of 24 colonies per strain.

### Measurement of RNA levels during mouse infection

For transcript level measurement during infection, mice limbs were immediately placed into RNAlater (Qiagen) and then snap frozen with liquid nitrogen. GAS RNA was isolated from mouse limbs as previously described [22]. In brief, the frozen limbs were subjected to vigorous mechanical lysis with a series of sharp blows using a three pound drill hammer and FastPrep Lysing Matrix B (MP Biomedicals). RNA was isolated using a Qiagen RNeasy kit and treated vigorously with Turbo DNAse (Ambion). cDNAs were prepared with and without reverse transcriptase to ensure that TaqMan QRT-PCR signal amplification did not reflect DNA contamination. Mouse limbs inoculated with PBS were also included in the analysis to ensure that the observed signal did not arise from eukaryotic RNA.

### Gene IDs [Entrez-Gene numbers]

*covR*, 3572611; *covS*, 3572612; *ccpA*, 3572471; *hpr*, 3571784; *hprK/P*, 3572422; *speB*, 3571136; *nga*, 3572762; *slo*, 3572764; *spyCEP*, 3760194; *sagA*, 3572347; *ftsX*, 3572932; *endoS*, 3571346; *hasA*, 3571023; *typA*, 3571645; *arcA*, 3571626; *amyA*, 3571845.

### Expression microarray data

Expression microarray data have been deposited at the Gene Expression Omnibus database at National Center for Biotechnology Information (http://www.ncbi.nlm.nih.gov/geo) and are accessible through accession number GSE20212.

## Supporting Information

Figure S1 Strain schematic and Southern blot analysis of GAS isogenic mutant strains used in this study. Isogenic mutant strains were derived (as indicated by solid lines) from the clinical serotype M1 isolates MGAS2221 (*covRS* wild-type) and MGAS5005 (Δ*covS*) as described in Materials and Methods. Dashed line between strains MGAS2221 and MGAS5005 indicates that the two strains are essentially genetically identical except for a truncated CovS protein in strain MGAS5005 [26],[65]. (A) Pictures show colony morphology of the indicated strains after growth on sheep blood agar plates overnight. (B, C) Southern blot of genomic DNA from the indicated GAS strains was digested with (B) *Kpn*I and (C) *Hind*III. (B) CcpA inactivation introduces a *Kpn*I restriction site reducing fragment size from 11 kb to 5.5 kb. (C) CovR inactivation eliminates a *Hind*III restriction site increasing fragment size from 3.6 kb to 4.2 kb.
(1.59 MB DOC)

Figure S2 Growth curves of various GAS strains and indicators of growth points used for RNA analysis. For (A), (B), and (C) the indicated strains were grown overnight and then placed into fresh THY at a 1:100 dilution. OD_600_ readings were taken hourly until the end of the experiment. For (A) and (B) arrows indicate points at which RNA was isolated for transcript level analysis by QRT-PCR. For (C) arrows indicate points at which RNA was isolated for expression microarray analysis.
(0.31 MB DOC)

Figure S3 Putative *cre* sites present in the gene regions of select GAS virulence factor genes. Shown are putative cre sites for the early intragenic (*speB, spyCEP*) and promoter (*nga/slo*) regions for the serotype M1 strain MGAS2221 and their relationship to transcription and translation start sites. The transcription start site for *speB* is not shown as there are multiple start sites some 600–1000 bps upstream of the translation start site.
(0.20 MB DOC)

Figure S4 CovR and CcpA do not influence transcript level of the other regulator. Transcript levels of *ccpA* (left) and *covR* (right) were determined in indicated strains at mid-exponential phase of growth in THY. For all panels data graphed are mean +/− standard deviation of four biological replicates done on two separate occasions (i.e. total of eight samples).
(0.06 MB DOC)

Figure S5 Principal component analysis of the CcpA and CovR transcriptome analysis. Four biological replicates of each of the indicated strains were grown to (A) mid-exponential (B) and stationary growth phases in THY with expression microarray analysis performed as described in Materials and Methods. Shown are principal components analyses (PCA) plots, which capture the variance in a dataset in terms of principal components and displays the three most significant of these on the X, Y, and Z axes.
(0.51 MB DOC)

Figure S6 Confirmatory QRT-PCR of expression microarray data and functional SpeB assay. (A to D) QRT-PCR analyzing transcript level of indicated genes encoding GAS virulence factors found to have significantly different transcript levels in isogenic mutant strains compared to wild-type by expression microarray analysis. Indicated strains were grown to labeled growth phases as detailed in Figure S2B. (E) Casein hydrolysis assays as marker of SpeB activity. For all panels, data graphed are mean +/− standard deviation of four biological replicates done on two separate occasions (i.e. total of 8 samples). * indicates P<0.05 compared to parental strain as determined by ANOVA followed by Tukey's post-hoc test. For all panels the relationship of bar color to GAS strain is indicated in legend.
(0.29 MB DOC)

Table S1 Growth characteristics of various strains in a laboratory medium (THY)
(0.04 MB DOC)

Table S2 Genes regulated by CcpA in strain MGAS2221 during growth in standard laboratory medium
(0.39 MB DOC)

Table S3 Genes regulated by CovR in strain MGAS2221 during growth in standard laboratory medium
(0.85 MB DOC)

Table S4 Genes with altered transcript levels in strain 2221Δ*covR*Δ*ccpA* vs. wild-type
(1.19 MB DOC)

Table S5 Primers and probes used in this study
(0.09 MB DOC)
