# Supplementary material for: A Combination of Independent Transcriptional Regulators Shapes Bacterial Virulence Gene Expression during Infection
Source: PLoS Pathog. 2010 Mar 19;6(3):e1000817. doi: 10.1371/journal.ppat.1000817 (PMC2841617; doi:10.1371/journal.ppat.1000817)
Supplement: Figure S1 — Strain schematic and Southern blot analysis of GAS isogenic mutant strains used in this study. Isogenic mutant strains were derived (as indicated by solid lines) from the clinical serotype M1 isolates MGAS2221 (covRS wild-type) and MGAS5005 (ΔcovS) as described in Materials and Methods. Dashed line between strains MGAS2221 and MGAS5005 indicates that the two strains are essentially genetically identical except for a truncated CovS protein in strain MGAS5005 [26],[65]. (A) Pictures show colony morphology of the indicated strains after growth on sheep blood agar plates overnight. (B, C) Southern blot of genomic DNA from the indicated GAS strains was digested with (B) KpnI and (C) HindIII. (B) CcpA inactivation introduces a KpnI restriction site reducing fragment size from 11 kb to 5.5 kb. (C) CovR inactivation eliminates a HindIII restriction site increasing fragment size from 3.6 kb to 4.2 kb. (1.59 MB DOC) [file ppat.1000817.s001.doc]

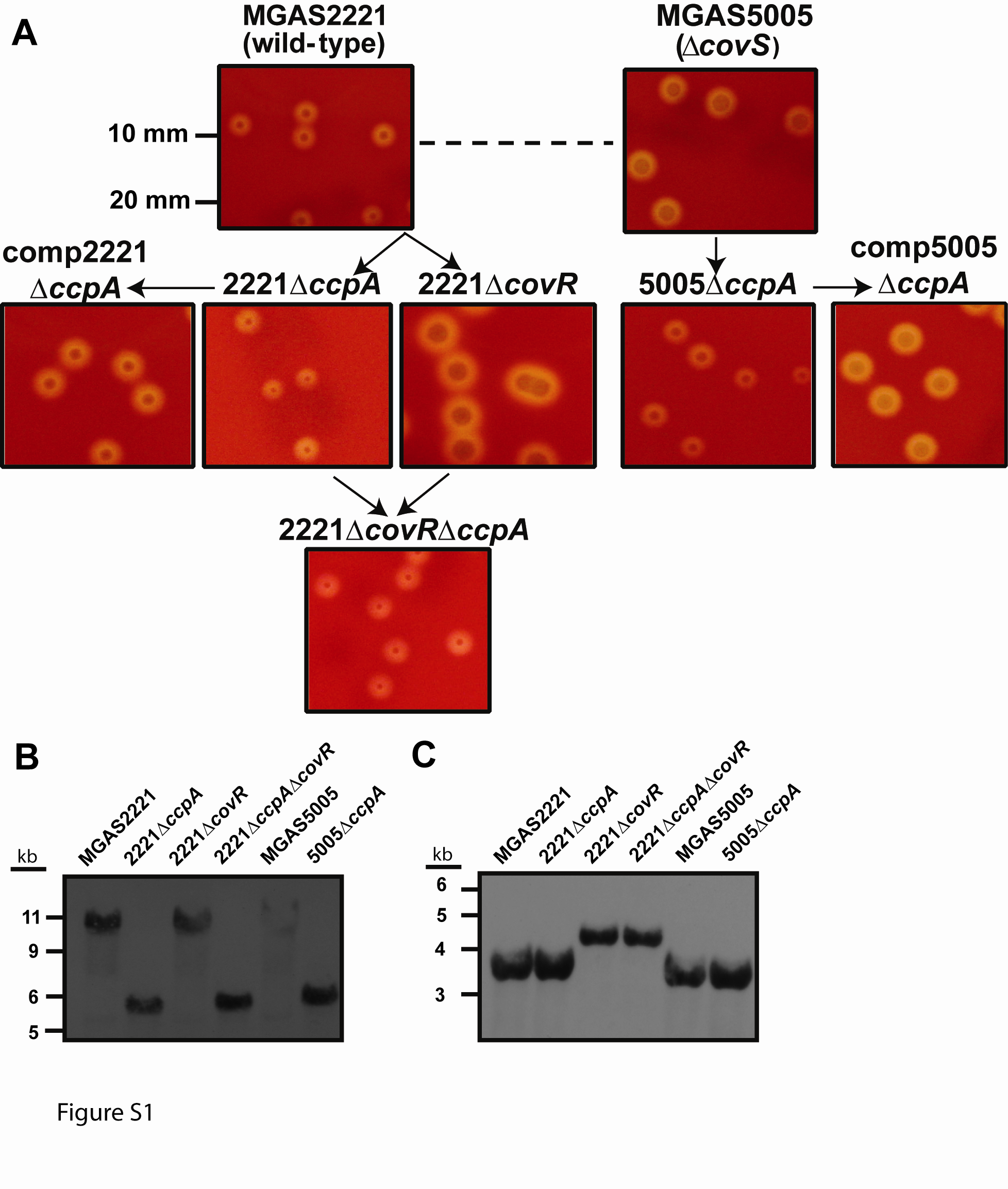


**Figure S1. Strain schematic and Southern blot analysis of GAS isogenic mutant strains used in this study.** Isogenic mutant strains were derived (as indicated by solid lines) from the clinical serotype M1 isolates MGAS2221 (*covRS* wild-type) and MGAS5005 (∆*covS*) as described in Materials and Methods. Dashed line between strains MGAS2221 and MGAS5005 indicates that the two strains are essentially genetically identical except for a truncated CovS protein in strain MGAS5005 [26,65]. (A) Pictures show colony morphology of the indicated strains after growth on sheep blood agar plates overnight. (B, C) Southern blot of genomic DNA from the indicated GAS strains was digested with (B) *Kpn*I and (C) *Hind*III. (B) CcpA inactivation introduces a *Kpn*I restriction site reducing fragment size from 11 kb to 5.5 kb. (C) CovR inactivation eliminates a *Hind*III restriction site increasing fragment size from 3.6 kb to 4.2 kb.
