# Supplementary material for: A Combination of Independent Transcriptional Regulators Shapes Bacterial Virulence Gene Expression during Infection
Source: PLoS Pathog. 2010 Mar 19;6(3):e1000817. doi: 10.1371/journal.ppat.1000817 (PMC2841617; doi:10.1371/journal.ppat.1000817)
Supplement: Figure S2 — Growth curves of various GAS strains and indicators of growth points used for RNA analysis. For (A), (B), and (C) the indicated strains were grown overnight and then placed into fresh THY at a 1:100 dilution. OD600 readings were taken hourly until the end of the experiment. For (A) and (B) arrows indicate points at which RNA was isolated for transcript level analysis by QRT-PCR. For (C) arrows indicate points at which RNA was isolated for expression microarray analysis. (0.31 MB DOC) [file ppat.1000817.s002.doc]

**
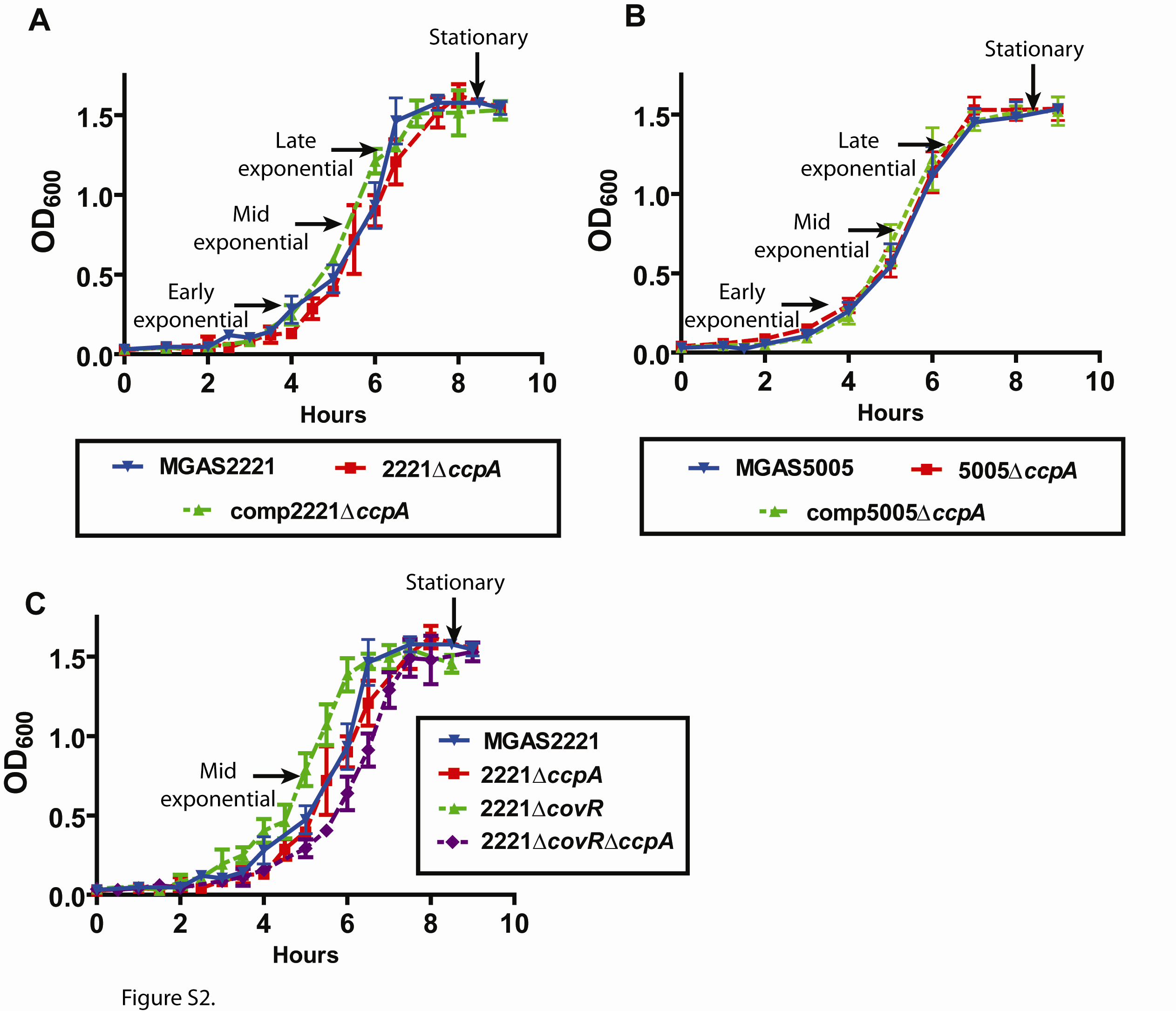
**

**Figure S2.** **Growth curves of various GAS strains and indicators of growth points used for RNA analysis.** For (A), (B), and (C) the indicated strains were grown overnight and then placed into fresh THY at a 1:100 dilution. OD_600_ readings were taken hourly until the end of the experiment. For (A) and (B) arrows indicate points at which RNA was isolated for transcript level analysis by QRT-PCR. For (C) arrows indicate points at which RNA was isolated for expression microarray analysis.
