# Supplementary material for: A Combination of Independent Transcriptional Regulators Shapes Bacterial Virulence Gene Expression during Infection
Source: PLoS Pathog. 2010 Mar 19;6(3):e1000817. doi: 10.1371/journal.ppat.1000817 (PMC2841617; doi:10.1371/journal.ppat.1000817)
Supplement: Figure S3 — Putative cre sites present in the gene regions of select GAS virulence factor genes. Shown are putative cre sites for the early intragenic (speB, spyCEP) and promoter (nga/slo) regions for the serotype M1 strain MGAS2221 and their relationship to transcription and translation start sites. The transcription start site for speB is not shown as there are multiple start sites some 600–1000 bps upstream of the translation start site. (0.20 MB DOC) [file ppat.1000817.s003.doc]

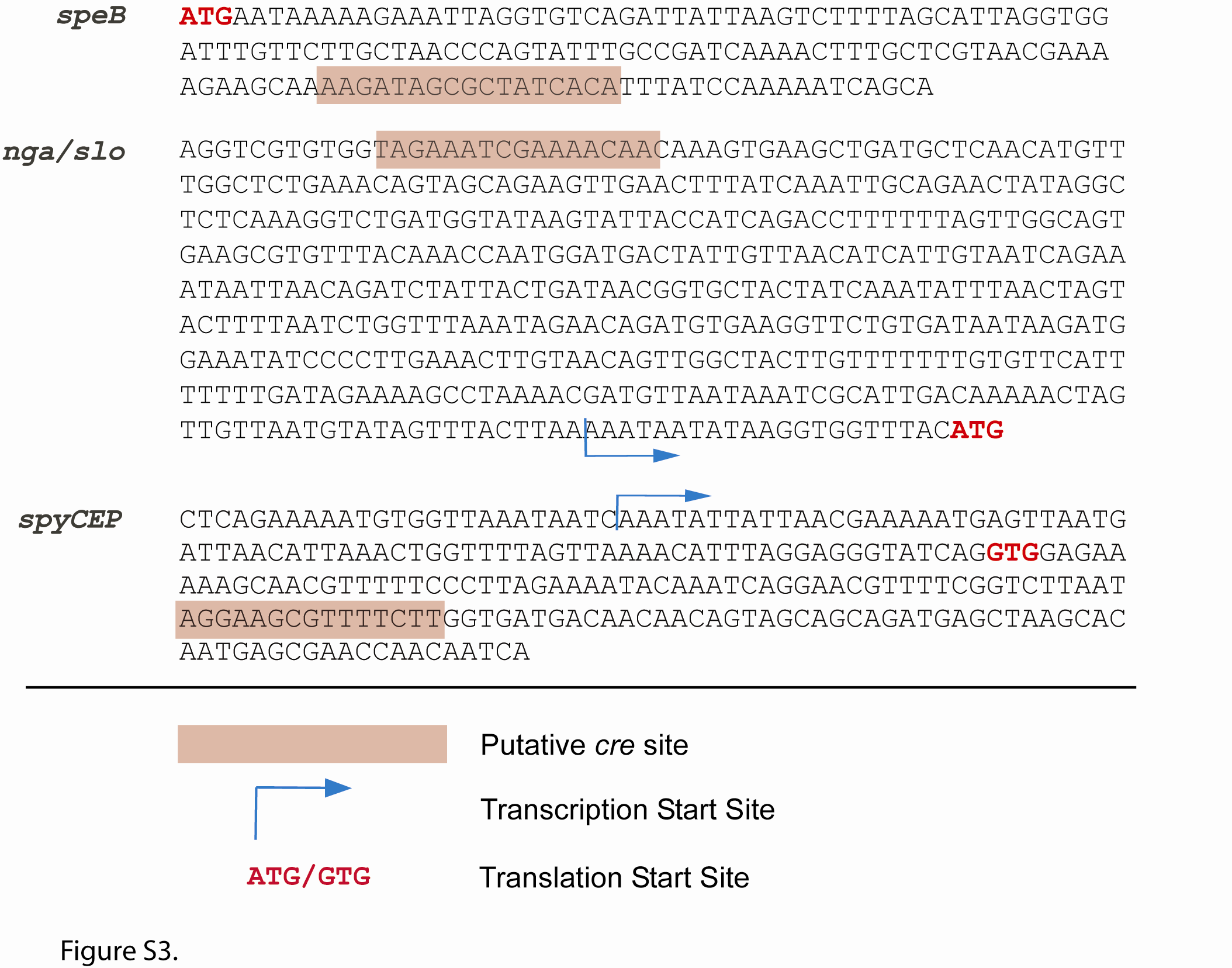


**Figure S3. Putative *cre* sites present in the gene regions of select GAS virulence factor genes.** Shown are putative *cre* sites for the early intragenic (*speB, spyCEP*) and promoter (*nga/slo*) regions for the serotype M1 strain MGAS2221 and their relationship to transcription and translation start sites. The transcription start site for *speB* is not shown as there are multiple start sites some 600-1000 bps upstream of the translation start site.
