# Supplementary material for: A Combination of Independent Transcriptional Regulators Shapes Bacterial Virulence Gene Expression during Infection
Source: PLoS Pathog. 2010 Mar 19;6(3):e1000817. doi: 10.1371/journal.ppat.1000817 (PMC2841617; doi:10.1371/journal.ppat.1000817)
Supplement: Figure S4 — CovR and CcpA do not influence transcript level of the other regulator. Transcript levels of ccpA (left) and covR (right) were determined in indicated strains at mid-exponential phase of growth in THY. For all panels data graphed are mean +/− standard deviation of four biological replicates done on two separate occasions (i.e. total of eight samples). (0.06 MB DOC) [file ppat.1000817.s004.doc]

**
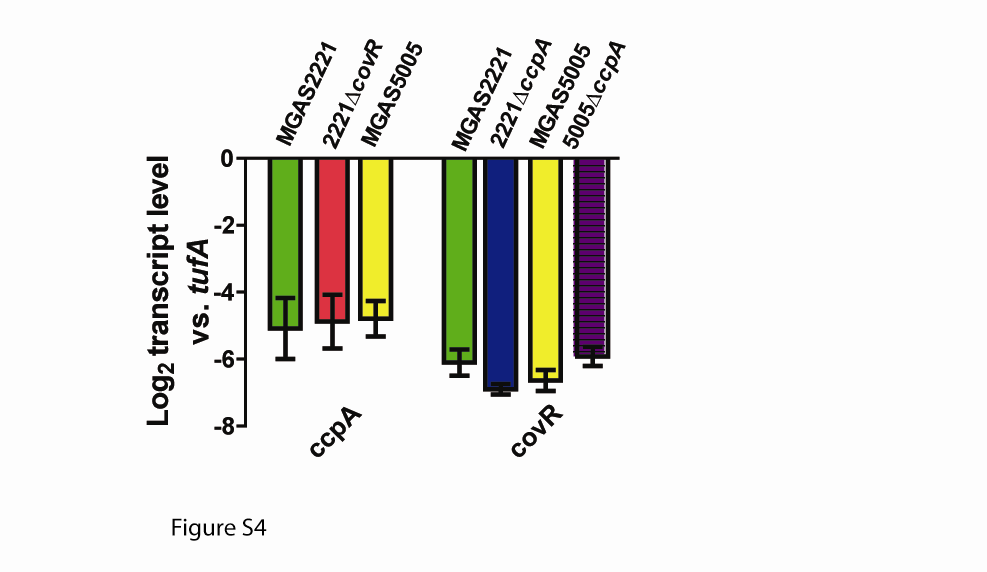
**

**Figure S4. CovR and CcpA do not influence transcript level of the other regulator.**  Transcript levels of *ccpA* (left) and *covR* (right) were determined in indicated strains at mid-exponential phase of growth in THY. For all panels data graphed are mean +/- standard deviation of four biological replicates done on two separate occasions (i.e. total of eight samples).
