# Supplementary material for: A Combination of Independent Transcriptional Regulators Shapes Bacterial Virulence Gene Expression during Infection
Source: PLoS Pathog. 2010 Mar 19;6(3):e1000817. doi: 10.1371/journal.ppat.1000817 (PMC2841617; doi:10.1371/journal.ppat.1000817)
Supplement: Figure S5 — Principal component analysis of the CcpA and CovR transcriptome analysis. Four biological replicates of each of the indicated strains were grown to (A) mid-exponential (B) and stationary growth phases in THY with expression microarray analysis performed as described in Materials and Methods. Shown are principal components analyses (PCA) plots, which capture the variance in a dataset in terms of principal components and displays the three most significant of these on the X, Y, and Z axes. (0.51 MB DOC) [file ppat.1000817.s005.doc]

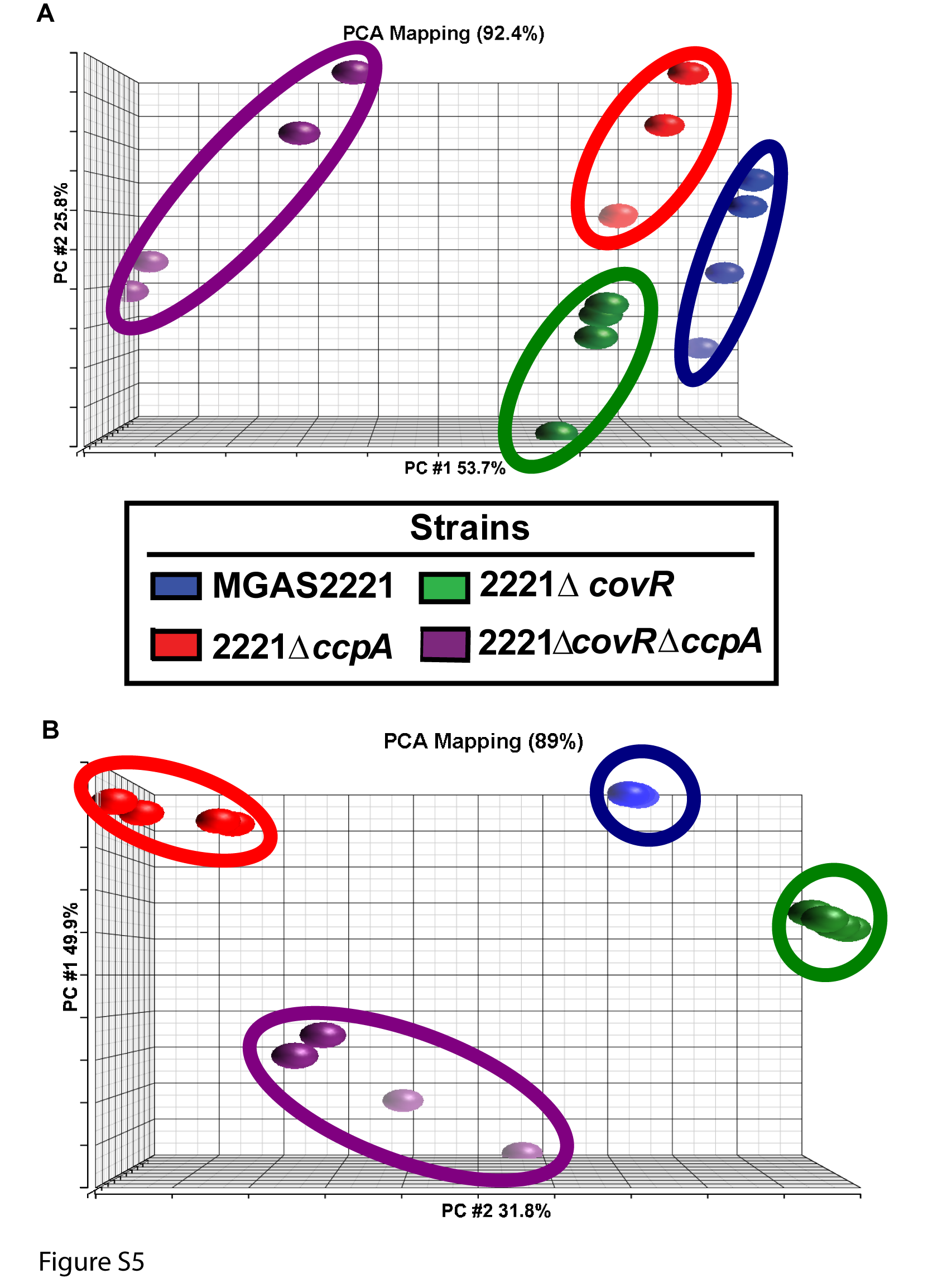


**Figure S5. Principal component analysis of the CcpA and CovR transcriptome analysis.** Four biological replicates of each of the indicated strains were grown to (A) mid-exponential (B) and stationary growth phases in THY with expression microarray analysis performed as described in Materials and Methods. Shown are principal components analyses (PCA) plots, which capture the variance in a dataset in terms of principal components and displays the three most significant of these on the X, Y, and Z axes.
