# Supplementary material for: A Combination of Independent Transcriptional Regulators Shapes Bacterial Virulence Gene Expression during Infection
Source: PLoS Pathog. 2010 Mar 19;6(3):e1000817. doi: 10.1371/journal.ppat.1000817 (PMC2841617; doi:10.1371/journal.ppat.1000817)
Supplement: Figure S6 — Confirmatory QRT-PCR of expression microarray data and functional SpeB assay. (A to D) QRT-PCR analyzing transcript level of indicated genes encoding GAS virulence factors found to have significantly different transcript levels in isogenic mutant strains compared to wild-type by expression microarray analysis. Indicated strains were grown to labeled growth phases as detailed in Figure S2B. (E) Casein hydrolysis assays as marker of SpeB activity. For all panels, data graphed are mean +/− standard deviation of four biological replicates done on two separate occasions (i.e. total of 8 samples). * indicates P<0.05 compared to parental strain as determined by ANOVA followed by Tukey's post-hoc test. For all panels the relationship of bar color to GAS strain is indicated in legend. (0.29 MB DOC) [file ppat.1000817.s006.doc]

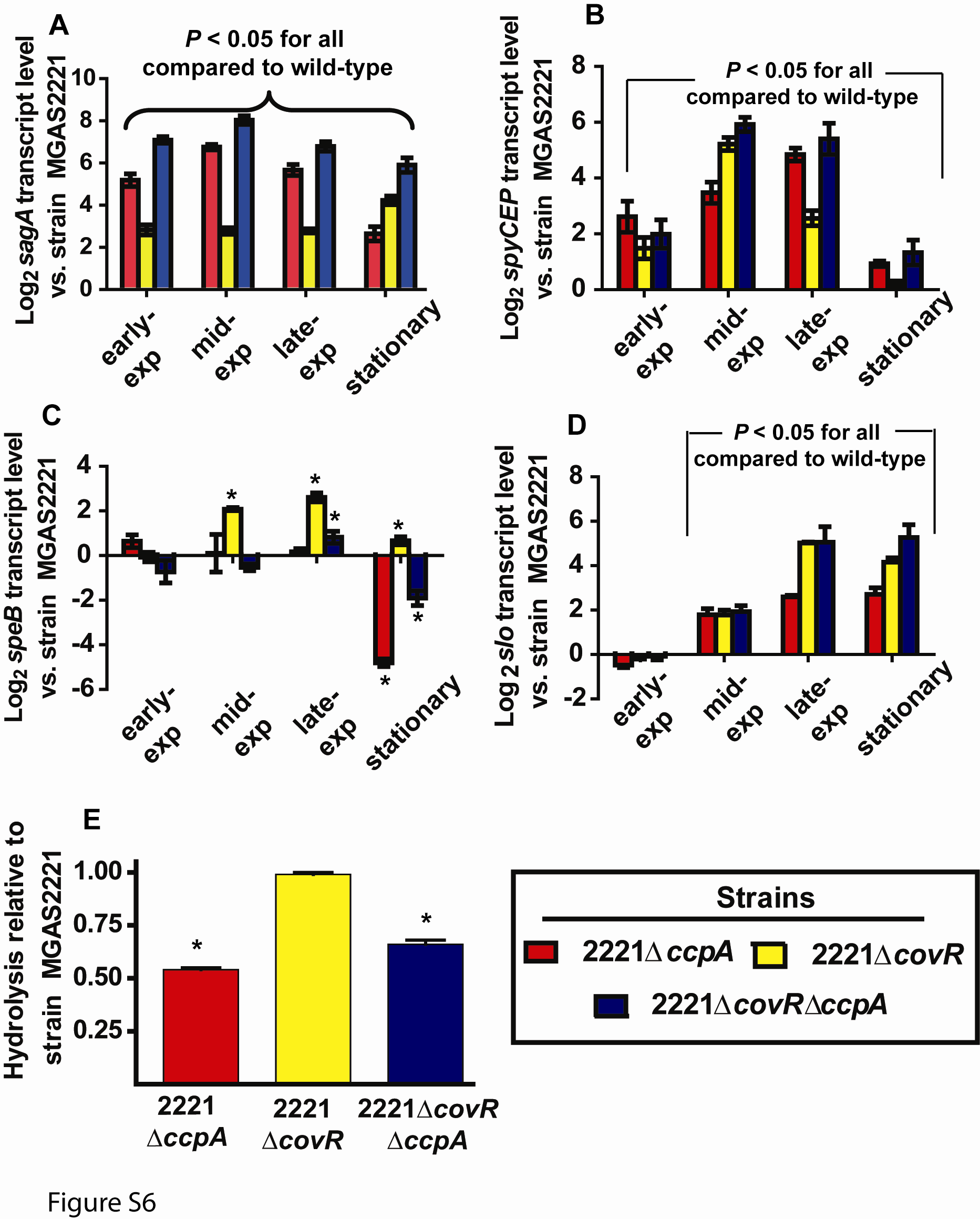


**Figure S6. Confirmatory QRT-PCR of expression microarray data and functional SpeB assay**. (A to D) QRT-PCR analyzing transcript level of indicated genes encoding GAS virulence factors found to have significantly different transcript levels in isogenic mutant strains compared to wild-type by expression microarray analysis. Indicated strains were grown to labeled growth phases as detailed in Figure S2B. (E) Casein hydrolysis assays as marker of SpeB activity. For all panels, data graphed are mean +/- standard deviation of four biological replicates done on two separate occasions (i.e. total of 8 samples).. * indicates *P* < 0.05 compared to parental strain as determined by ANOVA followed by Tukey’s post-hoc test. For all panels the relationship of bar color to GAS strain is indicated in legend.
