# Supplementary material for: A Combination of Independent Transcriptional Regulators Shapes Bacterial Virulence Gene Expression during Infection
Source: PLoS Pathog. 2010 Mar 19;6(3):e1000817. doi: 10.1371/journal.ppat.1000817 (PMC2841617; doi:10.1371/journal.ppat.1000817)
Supplement: Table S1 — Growth characteristics of various strains in a laboratory medium (THY) (0.04 MB DOC) [file ppat.1000817.s007.doc]

**Table S1 Growth characteristics of various strains in a laboratory medium (THY)**.

| Strain | Doubling time (min)^1^ | Final concentration^2^ | Organism density per colony (CFU) |
| --- | --- | --- | --- |
| MGAS2221 | 43.3 ± 5.1 | 1.54 ± 0.19 | 1.15 x 10^9^ ± 0.21 x 10^9^ |
| 2221∆*ccpA* | 45.6 ± 6.2 | 1.61 ± 0.21 | 1.22 x 10^9^ ± 0.20 x 10^9^ |
| comp2221∆*ccpA* | 47.3 ± 7.1 | 1.64 ± 0.18 | 1.19 x 10^9^ ± 0.14 x 10^9^ |
| MGAS5005 | 45.7 ± 5.7 | 1.59 ± 0.12 | 1.25 x 10^9^ ± 0.27 x 10^9^ |
| 5005∆*ccpA* | 47.8 ± 6.3 | 1.65 ± 0.23 | 1.22 x 10^9^ ± 0.20 x 10^9^ |
| comp5005∆*ccpA* | 48.8 ± 5.7 | 1.67 ± 0.15 | 1.09 x 10^9^ ± 0.31 x 10^9^ |
| 2221∆*covR* | 44.5 ± 6.2 | 1.63 ± 0.20 | 1.27 x 10^9^ ± 0.19 x 10^9^ |
| 2221∆*covR*∆*ccpA* | 47.8 ± 8.1 | 1.60 ± 0.23 | 1.07 x 10^9^ ± 0.34 x 10^9^ |

^1^Doubling-time determined by non-linear regression during exponential growth phase.

^2^OD_600_ reading.
