# Supplementary material for: A Combination of Independent Transcriptional Regulators Shapes Bacterial Virulence Gene Expression during Infection
Source: PLoS Pathog. 2010 Mar 19;6(3):e1000817. doi: 10.1371/journal.ppat.1000817 (PMC2841617; doi:10.1371/journal.ppat.1000817)
Supplement: Table S2 — Genes regulated by CcpA in strain MGAS2221 during growth in standard laboratory medium (0.39 MB DOC) [file ppat.1000817.s008.doc]

**Table S2 Genes regulated by CcpA in strain MGAS2221 during growth in standard laboratory medium**.

| **M5005 ORF** | **Gene name** | **Putative function of encoded protein** | **Fold-change^1^** | **Time-point** | **Fold-change in strain 2221∆*covR*^2^** | **Fold-change in strain 2221∆*covR* ∆*ccpA*** |
| --- | --- | --- | --- | --- | --- | --- |
| **Amino acid transport and metabolism** | | |  |  |  |  |
| *M5005_spy0834* |  | Zn-dependent alcohol dehydrogenase and related dehydrogenase | 2.44 | Mid | NS | 15.0/3.22 |
| *M5005_spy0982* |  | Histidine-binding protein | 4.70 | Stat | NS | NS |
| *M5005_spy0983* |  | Histidine transport ATP-binding protein | 3.10 | Stat | NS | NS |
| *M5005_spy0984* |  | Histidine transport permease protein | 4.60 | Stat | NS | NS |
| *M5005_spy1181* |  | Major tail protein | 3.14/2.08 | Mid/Stat | NS | 4.30/3.31 |
| *M5005_spy1237* | *artP* | Arginine transport ATP-binding protein | 8.80 | Stat | NS | 5.40 (S) |
| *M5005_spy1238* | *artQ* | Arginine transport system permease protein | 12.2 | Stat | NS | 4.10 (S) |
| *M5005_spy1269* | *asnA* | Aspartate--ammonia ligase/asparagine synthetase | 3.17 | Stat | 2.35 (M) | 2.20 (S) |
| *M5005_spy1270* | *arcC* | Carbamate kinase | 372/2.95 | Mid/Stat | 4.31/2.35 | 611/4.60 |
| *M5005_spy1271* |  | Xaa-His dipeptidase | 54.2/2.87 | Mid/Stat | 3.3/2.27 | 111/5.36 |
| *M5005_spy1272* |  | Arginine/ornithine antiporter | 26.4/2.58 | Mid/Stat | 4.15/3.12 | 53.9/4.25 |
| *M5005_spy1273* | *arcB* | Ornithine carbamoyltransferase | 19.0 | Mid | 3.52 (M) | 31.7/2.43 |
| *M5005_spy1274* |  | Acetyltransferase | 20.3 | Mid | 2.91(M) | 25.6 (M) |
| *M5005_spy1275* | *arcA* | Arginine deiminase | 22.1 | Mid | 2.27 (M) | 28.8 (M) |
| *M5005_spy1758* |  | Dipeptidase B | 3.08 | Mid | NS | 12.7/2.31 |
| *M5005_spy1770* | *hutI* | Imidazolonepropionase | 17.5/3.84 | Mid/Stat | 2.70 (S) | 60.4/13.1 |
| *M5005_spy1771* | *hutU* | Urocanate hydratase | 3.40 | Stat | 2.30 (S) | 4.62/4.07 |
| *M5005_spy1773* |  | Formiminotetrahydrofolate cyclodeaminase | 3.10 | Stat | 2.40 (S) | 2.26/3.22 |
| *M5005_spy1774* | *fhs.2* | Formate--tetrahydrofolate ligase | 2.70 | Stat | 2.60 (S) | 2.74/2.29 |
| *M5005_spy1775* |  | Hypothetical cytosolic protein | 6.37 | Stat | 2.90 (S) | 16.4/6.64 |
| *M5005_spy1776* |  | Amino acid permease | 4.10 | Stat | 2.30 (S) | 2.50/3.62 |
| *M5005_spy1777* | *hutH* | Histidine ammonia-lyase | 2.30 | Stat | 2.10 (S) | 4.60/4.71 |
| *M5005_spy1778* | *hutG* | Formiminoglutamase | 3.10 | Stat | 2.30 (S) | 2.29 (S) |
| **Carbohydrate transport and metabolism** | | |  |  |  |  |
| *M5005_spy0151* | *ulaD* | 3-keto-L-gulonate-6-phosphate decarboxylase | 2.09 | Mid | 3.37 (M) | 3.41 (M) |
| *M5005_spy0212* |  | N-acetylmannosamine-6-phosphate 2-epimerase | 15.17/5.79 | Mid/Stat | 5.57 (S) | 28.0/22.2 |
| *M5005_spy0213* |  | N-acetylneuraminate-binding protein | 12.4/7.04 | Mid/Stat | 5.36 (S) | 42.6/23.1 |
| *M5005_spy0214* |  | N-acetylneuraminate transport system permease protein | 9.92/11.4 | Mid/Stat | 6.28 (S) | 34.2/36.5 |
| *M5005_spy0215* |  | N-acetylneuraminate transport system permease protein | 28.5/4.73 | Mid/Stat | 7.16/2.51 | 142/18.8 |
| *M5005_spy0216* |  | Hypothetical protein | 15.2/6.13 | Mid/Stat | 3.63/4.84 | 136/55.3 |
| *M5005_spy0217* | *nanH* | N-acetylneuraminate lyase | 5.59/5.46 | Mid/Stat | 2.59/3.05 | 19.9/20.7 |
| *M5005_spy0218* |  | N-acetylmannosamine kinase | 5.95/6.51 | Mid/Stat | 2.21 (S) | 24.6/24.6 |
| *M5005_spy0361* |  | Phosphoglycerate transporter protein | 7.01/2.65 | Mid/Stat | NS | 12.8/4.08 |
| *M5005_spy0475* |  | PTS system, beta-glucoside-specific IIABC component | 5.90/8.41 | Mid/Stat | 2.17 (S) | 17.9/21.1 |
| *M5005_spy0476* | *bglA* | 6-phospho-beta-glucosidase | 5.24/9.58 | Mid/Stat | 2.06/9.58 | 21.0/20.7 |
| *M5005_spy0519* | *agaD* | PTS system, N-acetylgalactosamine-specific IID component | 3.72 | Mid | 4.22/2.42 | 12.4/2.35 |
| *M5005_spy0780* |  | PTS system, mannose/fructose family IIA component | 2.60 | Mid | NS | 2.72 (M) |
| *M5005_spy0781* | *ptsB* | PTS system, mannose/fructose family IIB component | 3.45 | Mid | NS | 2.11 (M) |
| *M5005_spy0782* | *ptsC* | PTS system, mannose/fructose family IIC component | 3.49 | Mid | NS | 2.38 (M) |
| *M5005_spy0783* | *ptsD* | PTS system, mannose/fructose family IID component | 3.43 | Mid | NS | 2.77 (M) |
| *M5005_spy1062* | *malA* | Maltodextrose utilization protein | 6.91/9.51 | Mid/Stat | 2.63 (S) | 26.9/26.7 |
| *M5005_spy1063* | *malD* | Maltodextrin transport system permease protein malD | 4.70/2.74 | Mid/Stat | 2.55 (S) | 8.81/4.55 |
| *M5005_spy1064* | *malC* | Maltodextrin transport system permease protein malC | 5.86/2.34 | Mid/Stat | 2.40 (S) | 6.08/3.90 |
| *M5005_spy1065* | *amyA* | Alpha-amylase | 8.14/2.32 | Mid/Stat | 2.62 (S) | 6.39/3.67 |
| *M5005_spy1066* | *amyB* | Neopullulanase/cyclomaltodextrinase/maltogenic alpha-amylase | 5.38/3.13 | Mid/Stat | 3.99 (S) | 10.0/5.91 |
| *M5005_spy1067* | *malX* | Maltose/maltodextrin-binding protein | 6.80/2.14 | Mid/Stat | 3.17 (S) | 9.14/4.09 |
| *M5005_spy1079* |  | PTS system, cellobiose-specific IIC component | 8.87/3.11 | Mid/Stat | 2.16 (S) | 17.2/11.4 |
| *M5005_spy1081* |  | PTS system, cellobiose-specific IIA component | 7.61/1.84 | Mid/Stat | 2.22 (S) | 8.97/5.64 |
| *M5005_spy1082* |  | PTS system, cellobiose-specific IIB component | 8.88 | Mid | 2.08 (S) | 13.1/6.71 |
| *M5005_spy1083* |  | PTS system, mannitol (cryptic)-specific IIA component/Transcription antiterminator, BglG family | 7.67/2.49 | Mid/Stat | 3.36/2.11 | 28.6/5.10 |
| *M5005_spy1308* |  | Sugar-binding protein | 2.06 | Mid | 3.54 (S) | 3.82/3.01 |
| *M5005_spy1309* |  | Sugar transport system permease protein | 2.92 | Mid | 5.85 (S) | 9.12/4.55 |
| *M5005_spy1375* | *tkt* | Transketolase | 2.34 | Mid | NS | 2.86/2.15 |
| *M5005_spy1376* |  | Transaldolase | 4.20/2.77 | Mid/Stat | 2.28 (S) | 6.13/5.61 |
| *M5005_spy1379* | *glpF* | Glycerol uptake facilitator protein | 11.2/2.84 | Mid/Stat | 2.02 (S) | 21.2/9.56 |
| *M5005_spy1395* | *lacD.1* | Tagatose-bisphosphate aldolase | 5.39 | Mid | 3.88 (S) | 17.1/7.83 |
| *M5005_spy1396* | *nadE* | Tagatose-6-phosphate kinase | 6.23 | Mid | 6.23 (M) | 9.02/6.69 |
| *M5005_spy1397* | *lacB.1* | Galactose-6-phosphate isomerase lacB subunit | 9.12 | Mid | 6.87 (S) | 20.8/10.2 |
| *M5005_spy1398* | *lacA.1* | Galactose-6-phosphate isomerase lacA subunit | 7.21/2.80 | Mid/Stat | 2.27 (S) | 56.8/22.5 |
| *M5005_spy1399* |  | PTS system, galactose-specific IIC component | 5.96 | Mid | 4.56 (S) | 16.5/3.35 |
| *M5005_spy1400* |  | PTS system, galactose-specific IIB component | 8.25 | Mid | 3.59 (S) | 10.1/2.64 |
| *M5005_spy1401* |  | PTS system, galactose-specific IIA component | 7.13 | Mid | 3.44 (S) | 20.2/2.18 |
| *M5005_spy1538* | *pmi* | Mannonse-6-phosphate isomerase | 9.02 | Stat | 3.03 (S) | 24.0 (S) |
| *M5005_spy1539* | *scrK* | Fructokinase | 25.0 | Stat | NS | 35.6 (S) |
| *M5005_spy1542* | *scrA* | PTS system, sucrose-specific IIABC component | 24.2 | Stat | 3.23 (S) | 30.3 (S) |
| *M5005_spy1543* | *scrB* | Sucrose-6-phosphate hydrolase | 9.02 | Stat | NS | 8.9 (S) |
| *M5005_spy1632* | *lacG* | 6-phospho-beta-galactosidase | -3.21/8.91 | Mid/Stat | 2.33 (S) | 28.8 (S) |
| *M5005_spy1633* | *lacE* | PTS system, lactose-specific IIBC component | -2.52/7.52 | Mid/Stat | 2.71 (S) | 16.8 (S) |
| *M5005_spy1634* | *lacF* | PTS system, lactose-specific IIA component | -2.71/6.39 | Mid/Stat | 2.47 (S) | 13.8 (S) |
| *M5005_spy1635* | *lacD.2* | Tagatose 1,6-diphosphate aldolase | -1.91/5.73 | Mid/Stat | NS | 10.6 (S) |
| *M5005_spy1636* | *lacC.2* | Tagatose-6-phosphate kinase | -2.03/8.21 | Mid/Stat | 2.51 (S) | 14.3 (S) |
| *M5005_spy1637* | *lacB.2* | Galactose-6-phosphate isomerase LacB subunit | -3.22/7.56 | Mid/Stat | 2.37 (S) | 13.9 (S) |
| *M5005_spy1638* | *lacA.2* | Galactose-6-phosphate isomerase LacA subunit | -2.97/6.39 | Mid/Stat | 3.21 (S) | 10.3 (S) |
| *M5005_spy1661* |  | Transaldolase | 3.35 | Mid | 2.48 (S) | 8.94/6.55 |
| *M5005_spy1662* | *ulaA* | Ascorbate-specific PTS system enzyme IIC | 5.78/2.91 | Mid/Stat | 2.01 (M) | 12.4/5.71 |
| *M5005_spy1663* |  | PTS system, IIB component | 5.89/4.86 | Mid/Stat | 2.37/5.47 | 23.8/18.4 |
| *M5005_spy1664* |  | PTS system, mannitol (cryptic)-specific IIA component/Transcription antiterminator, BglG family | 3.10 | Mid | NS | 5.09/3.09 |
| *M5005_spy1693* | *ptsG* | PTS system, glucose-specific IIABC component | 8.72 | Stat | 4.16 (S) | 2.35 (M) |
| *M5005_spy1744* |  | PTS system, cellobiose-specific IIC component | 2.07/3.31 | Mid/Stat | 3.06 (S) | 6.22/5.46 |
| *M5005_spy1745* |  | PTS system, cellobiose-specific IIB component | 4.31/3.56 | Mid/Stat | 2.09/4.28 | 15.9/6.45 |
| *M5005_spy1746* |  | PTS system, cellobiose-specific IIA component | 3.03/2.00 | Mid/Stat | 3.23 (S) | 10.7/4.69 |
| **Cellular processing** | |  |  |  |  |  |
| *M5005_spy0452* |  | Chromosome segregation ATPase | 3.24 | Mid | NS | NS |
| *M5005_spy0453* |  | Chromosome segregation ATPase | 2.14 | Mid | NS | NS |
| *M5005_spy0835* |  | Class B acid phosphatase | 2.74 | Mid | NS | 5.49/4.78 |
| *M5005_spy0836* |  | Acid phosphatase/phosphotransferase | 3.10 | Mid | NS | 7.01/4.11 |
| **Cell wall/membrane biogenesis** | | |  |  |  |  |
| *M5005_spy1448* |  | Hypothetical protein | 2.22 | Mid | NS | NS |
| *M5005_spy1843* |  | Transglycosylase | 5.54 | Stat | NS | 5.28/6.88 |
| **Coenzyme transport and metabolism** | | |  |  |  |  |
| *M5005_spy0908* | *citX* | 2’-(5’’-triphosphoribosyl)-3’-dephospho-CoA:apo-citrate lyase | 5.90 | Mid | NS | 5.96/3.40 |
| *M5005_spy1086* |  | Nicotinamide mononucleotide transporter | 2.59 | Stat | 2.08 (S) | NS |
| **Defence mechanisms/virulence** | | |  |  |  |  |
| *M5005_spy0139* | *nga* | NAD glycohydrolase | 3.56 | Stat | 12.1/21.3 | 31.2/28.6 |
| *M5005_spy0141* | *slo* | Streptolysin O | 2.73 | Stat | 12.3/25.2 | 25.3/32.5 |
| *M5005_spy0341* | *spyCEP* | IL-8-degrading proteinase | 8.33/7.43 | Mid/Stat | 17.4/10.1 | 25.5/34.2 |
| *M5005_spy0562* | *sagA* | Streptolysin S precursor | 2.68 | Mid | 2.31/5.34 | 8.35/8.36 |
| *M5005_spy0563* | *sagB* | Streptolysin S biosynthesis protein sagB | 3.65/5.28 | Mid/Stat | 3.21/15.5 | 10.5/19.2 |
| *M5005_spy0564* | *sagC* | Streptolysin S biosynthesis protein sagC | 3.12/4.12 | Mid/Stat | 2.81/8.71 | 8.84/15.3 |
| *M5005_spy0565* | *sagD* | Streptolysin S biosynthesis protein sagD | 3.82/4.60 | Mid/Stat | 4.02/10.0 | 9.79/12.5 |
| *M5005_spy0566* | *sagE* | Streptolysin S putative self-immunity protein sagE | 3.73/5.84 | Mid/Stat | 3.01/14.9 | 10.4/19.3 |
| *M5005_spy0567* | *sagF* | Streptolysin S biosynthesis protein sagF | 3.14/4.65 | Mid/Stat | 2.56/11.2 | 6.15/9.57 |
| *M5005_spy0568* | *sagG* | Streptolysin S export ATP-binding protein sagG | 2.57/3.45 | Mid/Stat | 2.07/6.57 | 5.32/8.30 |
| *M5005_spy0569* | *sagH* | Streptolysin S export transmembrane protein sagH | 3.03/3.93 | Mid/Stat | 2.38/8.12 | 6.50/10.1 |
| *M5005_spy0570* | *sagI* | Streptolysin S export transmembrane protein sagI | 2.23/3.01 | Mid/Stat | 2.15/5.51 | 5.22/6.94 |
| *M5005_spy1415* | *sdaD2* | Streptodornase | 2.31 | Stat | 2.69/7.86 | 2.20/10.1 |
| *M5005_spy1540* | *endoS* | Endo-beta-N-acetylglucosaminidase F2 precursor | 21.3 | Stat | 2.61 (S) | 26.6 (S) |
| *M5005_spy1688* |  | Immunoglobulin receptor precursor | 2.29 | Mid | 2.29 (M) | 98.2/35.3 |
| *M5005_spy1689* |  | Collagen-like surface protein | -2.05 | Mid | 21.4/196 | 19.2/453 |
| *M5005_spy1691* |  | Endonuclease/exonuclease/phosphatase family protein | 3.15/2.01 | Mid/Stat | 2.97/4.24 | 7.65 (M) |
| *M5005_spy1714* | *fba* | Fibronectin-binding protein | 3.23 | Stat | 4.12 (S) | 5.01 (S) |
| *M5005_spy1715* | *scpA* | C5a peptidase precursor | 2.23 | Stat | 5.13 (S) | 5.23 (S) |
| *M5005_spy1735* | *speB* | Cysteine Protease | -5.21 | Mid | 2.23 (M) | -5.23 (M) |
| **Energy production and conversion** | | |  |  |  |  |
| *M5005_spy0040* | *adhA* | Alcohol dehydrogenase | 5.91/2.52 | Mid/Stat | NS | NS |
| *M5005_spy0094* | *ackA* | Acetate kinase | 2.13 | Stat | NS | NS |
| *M5005_spy0126* | *ntpI* | V-type sodium ATP synthase subunit I | 12.3/0.18 | Mid/Stat | 5.08 (S) | 4.32/29.2 |
| *M5005_spy0127* | *ntpK* | V-type sodium ATP synthase subunit K | 11.3/3.11 | Mid/Stat | 2.88 (S) | 3.58/20.9 |
| *M5005_spy0128* | *ntpE* | V-type sodium ATP synthase subunit E | 11.0/3.61 | Mid/Stat | 2.37 (S) | 4.21/17.0 |
| *M5005_spy0129* | *ntpC* | V-type sodium ATP synthase subunit C | 18.9/4.09 | Mid/Stat | 2.45/2.50 | 7.92/26.5 |
| *M5005_spy0130* | *ntpF* | V-type sodium ATP synthase subunit F | 11.8/3.11 | Mid/Stat | 2.31 (S) | 3.92/13.6 |
| *M5005_spy0131* | *ntpA* | V-type sodium ATP synthase subunit A | 9.47/2.85 | Mid/Stat | NS | 3.46/12.1 |
| *M5005_spy0132* | *ntpB* | V-type sodium ATP synthase subunit B | 12.6/3.61 | Mid/Stat | NS | 4.87/19.4 |
| *M5005_spy0133* | *ntpD* | V-type sodium ATP synthase subunit D | 14.4/3.32 | Mid/Stat | NS | 4.83/18.5 |
| *M5005_spy0340* | *lctO* | L-lactate oxidase | 13.8/2.19 | Mid/Stat | NS | 39.4/6.16 |
| *M5005_spy0900* |  | Mg2+/citrate complex secondary transporter | 3.00 | Stat | 2.49 (S) | 5.76/7.97 |
| *M5005_spy1380* | *glpO* | Alpha-glycerophosphate oxidase | 5.07/3.23 | Mid/Stat | NS | 11.2/9.97 |
| *M5005_spy1381* | *glpK* | Glycerol kinase | 2.63 | Mid | NS | 8.27/7.06 |
| **Inorganic ion transport and metabolism** | | |  |  |  |  |
| *M5005_spy0321* | *fhuG* | Ferrichrome transport system permease protein | 2.83 | Stat | NS | NS |
| *M5005_spy0322* | *fhuB* | Ferrichrome transport system permease protein | 2.72 | Stat | NS | NS |
| *M5005_spy0323* | *fhuD* | Ferrichrome-binding protein | 3.16 | Stat | NS | NS |
| *M5005_spy0324* | *fhuA* | Ferrichrome transport ATP-binding protein | 4.44 | Stat | NS | NS |
| *M5005_spy1161* |  | Formate transporter | 2.04 | Stat | 2.79 (S) | 3.16/2.71 |
| **Lipid transport and metabolism** | | |  |  |  |  |
| *M5005_spy0120* | *atoD.2* | Acetate Co-A-transferase alpha subunit | 2.74 | Stat | 3.82 (S) | 2.87/6.96 |
| *M5005_spy0535* |  | Acetoin dehydrogenase | 2.30 | Mid | 2.02 (S) | 5.12/1.68 |
| **Nucleotide transport and metabolism** | | |  |  |  |  |
| *M5005_spy0080* |  | Bis(5’-nucleosyl)-tetraphosphatase | 5.82 | Stat | -4.90 (S) | 3.22 (S) |
| *M5005_spy0347* | *nrdF* | Ribonucleoside-diphosphate reductase beta chain | 2.10/3.41 | Mid/Stat | NS | NS |
| *M5005_spy0348* | *nrdI* | Nucleotide reductase stimulatory protein NrdI | 2.03/2.04 | Mid/Stat | NS | NS |
| *M5005_spy0349* | *nrdE.1* | Ribonucleoside-diphosphate reductase alpha chain | 2.17 | Mid | NS | NS |
| *M5005_spy0678* |  | 5 –nucleotidase | 2.25/4.59 | Mid/Stat | NS | 4.89/8.65 |
| **Phage** | |  |  |  |  |  |
| *M5005_spy0459* |  | Portal protein | 5.01 | Mid | 2.26 (M) | 5.99 (M) |
| *M5005_spy0995* |  | Phage protein | 2.73/4.82 | Mid/Stat | 2.51 (M) | 8.54/11.9 |
| *M5005_spy0999* |  | Phage protein | 2.09 | Mid | NS | 8.82/2.20 |
| *M5005_spy1021* |  | Phage protein | 3.21 | Stat | 4.53 (M) | 13.2/9.11 |
| *M5005_spy1172* |  | Holin | 2.35 | Mid | NS | 2.90 (M) |
| *M5005_spy1173* |  | Phage protein | 2.88 | Mid | 5.35 (M) | 5.08 (M) |
| *M5005_spy1174* |  | Phage protein | -2.10 | Mid | NS | NS |
| *M5005_spy1176* |  | Phage infection protein | 4.49/3.29 | Mid/Stat | NS | 2.08/5.87 |
| *M5005_spy1185* |  | Phage protein | 2.74 | Mid | NS | NS |
| *M5005_spy1197* |  | Phage protein | -3.21 | Mid | NS | NS |
| *M5005_spy1201* |  | Phage protein | 3.81 | Mid | NS | 2.75 (M) |
| **Post-translational modification, protein turnover and chaperones** | | |  |  |  |  |
| *M5005_spy1080* |  | Hypothetical protein | 2.39/2.45 | Mid/Stat | NS | 8.76/7.30 |
| *M5005_spy1282* | *msrA* | Bifunctional methionine sulfoxide reductase A/B peptide | 3.44 | Mid | NS | 3.44 (M) |
| *M5005_spy1283* | *tlpA* | Thiol:disulfide interchange protein tlpA | 4.20 | Mid | NS | NS |
| **Replication, recombination and repair** | | |  |  |  |  |
| *M5005_spy0254* |  | Transposase | 4.70/2.32 | Mid/Stat | 4.50 (M) | 6.48/11.7 |
| *M5005_spy0651* | *bspA* | Cell surface protein | 2.50 | Stat | NS | NS |
| *M5005_spy0712* | *parC* | DNA topoisomerase IV subunit A | 2.04 | Mid | NS | NS |
| *M5005_spy1643* |  | DNA integration/recombination/inversion protein | 2.42 | Mid | NS | 2.48 (M) |
| **Stress** | |  |  |  |  |  |
| *M5005_spy1378* |  | NADH peroxidase | 6.47/3.08 | Mid/Stat | NS | 13.2/8.21 |
| **Transcription** | | |  |  |  |  |
| *M5005_spy0117* |  | Transcriptional regulator, LysR family | 4.91/4.27 | Mid/Stat | NS | 14.1/16.5 |
| *M5005_spy0118* |  | Transcriptional regulator, LysR family | 4.73/4.64 | Mid/Stat | NS | 15.9/16.4 |
| *M5005_spy0474* | *licT* | Transcription antiterminator, BglG family | 6.41/12.2 | Mid/Stat | NS | 17.0/30.6 |
| *M5005_spy1377* |  | Trans-acting positive regulator | 4.78/2.49 | Mid/Stat | NS | 10.5/6.52 |
| *M5005_spy1760* |  | Transcriptional regulator, MutR family | 8.39 | Mid | 4.63 (M) | 39.6/4.59 |
| *M5005_spy1779* |  | Transcriptional regulator, LuxR family | 11.79/7.66 | Mid/Stat | NS | 23.1/16.8 |
| **Translation** | |  |  |  |  |  |
| *M5005_spy0081* | *tyrS* | Tyrosyl tRNA synthetase | 5.01 | Stat | -4.88 (S) | NS |
| *M5005_spy0798* |  | IFN-response binding factor 1 | 3.38 | Mid | 5.92 (M) | 4.30 (M) |
| **Unknown** | |  |  |  |  |  |
| *M5005_spy0017* |  | Secreted protein of unknown function | 2.73 | Stat | -2.25 (S) | NS |
| *M5005_spy0125* |  | Hypothetical protein | 12.5/4.29 | Mid/Stat | NS | 4.47/30.7 |
| *M5005_spy0346* |  | Hypothetical protein | 2.01/2.96 | Mid/Stat | NS | NS |
| *M5005_spy0401* |  | Hypothetical cytosolic protein | 4.23 | Mid | 3.59 (M) | NS |
| *M5005_spy0454* |  | Hypothetical protein | 2.43 | Mid | 2.78 (M) | NS |
| *M5005_spy0481* |  | Hypothetical protein | 2.32 | Mid | NS | NS |
| *M5005_spy0518* |  | Oligohyaluronate lyase | 2.21/2.12 | Mid/Stat | 2.41/6.17 | 5.71/11.3 |
| *M5005_spy0773* |  | Hypothetical protein | 5.63 | Mid | 7.48/3.49 | 14.8/4.99 |
| *M5005_spy0812* |  | Hypothetical protein | 4.29 | Mid | 3.35 (M) | 5.09 (M) |
| *M5005_spy0861* |  | Hypothetical protein | 5.12 | Stat | -3.93 (S) | NS |
| *M5005_spy1078* |  | Hypothetical protein | 3.66/2.17 | Mid/Stat | NS | 6.58/3.66 |
| *M5005_spy1084* |  | Outer surface protein | 4.35/3.31 | Mid/Stat | NS | 53.9/41.8 |
| *M5005_spy1093* |  | Hypothetical protein | 7.49 | Mid | NS | 29.1/6.00 |
| *M5005_spy1541* |  | Hypothetical protein | 27.3 | Stat | 3.05 (S) | 33.2 (S) |
| *M5005_spy1644* |  | Hypothetical protein | 4.95/3.56 | Mid/Stat | NS | NS |
| *M5005_spy1667* |  | Hypothetical protein | 3.50 | Mid | 3.22 (M) | 4.07 (M) |
| *M5005_spy1703* |  | Hypothetical cytosolic protein | 4.41 | Mid | 6,81 (M) | 8.10 (M) |
| *M5005_spy1860* |  | Hypothetical protein | 2.23 | Stat | -2.80 (S) | NS |

^1^positive numbers indicated increased transcript levels in mutant strain, whereas negative numbers indicate higher transcript levels in wild-type strain

^2^NS = no significant difference in transcript level between wild-type and isogenic mutant strain. If 2 numbers are listed then transcript levels were significantly different in both the mid-exponential and stationary growth phases. If 1 number is listed then the transcript level was only significantly different at one growth phase which is indicated by (M) for mid-exponential and (S) for stationary.
