# Supplementary material for: A Combination of Independent Transcriptional Regulators Shapes Bacterial Virulence Gene Expression during Infection
Source: PLoS Pathog. 2010 Mar 19;6(3):e1000817. doi: 10.1371/journal.ppat.1000817 (PMC2841617; doi:10.1371/journal.ppat.1000817)
Supplement: Table S3 — Genes regulated by CovR in strain MGAS2221 during growth in standard laboratory medium (0.85 MB DOC) [file ppat.1000817.s009.doc]

**Table S3 Genes regulated by CovR in strain MGAS2221 during growth in standard laboratory medium.**

| **M5005 ORF** | **Gene name** | **Putative function of encoded protein** | **Fold-change^1^** | **Time-point** | **Fold-change in strain 2221∆*ccpA*^2^** | **Fold-change in strain 2221∆*covR* ∆*ccpA*** |
| --- | --- | --- | --- | --- | --- | --- |
| **Amino acid transport and metabolism** | | |  |  |  |  |
| *M5005_spy0146* | *metB* | Cystathionine beta-lyase | 2.45 | Mid | NS | 2.90 (M) |
| *M5005_spy1269* | *asnA* | Aspartate—ammonia ligase/asparagine synthetase | 2.35 | Mid | 3.17 (S) | 2.20 (S) |
| *M5005_spy1270* | *arcC* | Carbamate kinase | 4.31/2.35 | Mid/Stat | 372/2.95 | 611/4.60 |
| *M5005_spy1271* |  | Xaa-His dipeptidase | 3.93/2.27 | Mid/Stat | 54.2/2.87 | 111/5.36 |
| *M5005_spy1272* |  | Arginine/ornithine antiporter | 4.15/3.12 | Mid/Stat | 26.4/2.58 | 53.6/4.25 |
| *M5005_spy1273* | *arcB* | Ornithine carbamoyltransferase | 3.52 | Mid | 19.0 (M) | 31.7/2.43 |
| *M5005_spy1274* |  | Acetyltransferase | 2.91 | Mid | 20.3 (M) | 25.6 (M) |
| *M5005_spy1275* | *arcA* | Arginine deiminase | 2.27 | Mid | 22.1 (M) | 28.8 (M) |
| *M5005_spy1704* | *dppA* | Dipeptide transport | -3.13 | Mid | NS | -5.13 (M) |
| *M5005_spy1705* | *dppB* | Dipeptide transport | -2.97 | Mid | NS | -5.83 (M) |
| *M5005_spy1706* | *dppC* | Dipeptide transport | -2.80 | Mid | NS | -5.29 (M) |
| *M5005_spy1707* | *dppD* | Dipeptide transport | -2.71 | Mid | NS | -4.91 (M) |
| *M5005_spy1708* | *dppE* | Dipeptide transport | -2.56 | Mid | NS | -5.26 (M) |
| *M5005_spy1770* | *hutI* | Imidazolonepropionase | 2.70 | Stat | 17.5/3.84 | 60.4/13.1 |
| *M5005_spy1771* | *hutU* | Urocanate hydratase | 2.30 | Stat | 3.40 (S) | 4.62/4.07 |
| *M5005_spy1773* |  | Formiminotetrahydrofolate cyclodeaminase | 2.40 | Stat | 3.10 (S) | 2.26/3.22 |
| *M5005_spy1774* | *fhs.2* | Formate—tetrahydrofolate ligase | 2.60 | Stat | 2.70 (S) | 2.74/2.29 |
| *M5005_spy1775* |  | Hypothetical cytosolic protein | 2.90 | Stat | 6.37 (S) | 16.4/6.64 |
| *M5005_spy1776* |  | Amino acid permease | 2.30 | Stat | 4.10 (S) | 2.50/3.62 |
| *M5005_spy1777* | *hutH* | Histidine ammonia-lyase | 2.10 | Stat | 2.30 (S) | 4.60/4.74 |
| *M5005_spy1778* | *hutG* | Formiminoglutamase | 2.30 | Stat | 3.10 (S) | 2.29 (S) |
| **Carbohydrate transport and metabolism** | | |  |  |  |  |
| *M5005_spy0151* | *ulaD* | 3-keto-L-gulonate-6-phosphate decarboxylase | 3.37 | Mid | 2.09 (M) | 3.41 (M) |
| *M5005_spy0212* |  | N-acetylmannosamine-6-phosphate 2-epimerase | 5.57 | Stat | 15.17/5.79 | 28.0/22.2 |
| *M5005_spy0213* |  | N-acetylneuraminate-binding protein | 5.36 | Stat | 12.4/7.04 | 42.6/23.1 |
| *M5005_spy0214* |  | N-acetylneuraminate transport system permease protein | 6.28 | Stat | 9.92/11.4 | 34.2/36.5 |
| *M5005_spy0215* |  | N-acetylneuraminate transport system permease protein | 7.16/2.51 | Mid/Stat | 28.5/4.73 | 142/18.8 |
| *M5005_spy0216* |  | Hypothetical protein | 3.63/4.84 | Mid/Stat | 15.2/6.13 | 136/55.3 |
| *M5005_spy0217* | *nanH* | N-acetylneuraminate lyase | 2.59/3.05 | Mid/Stat | 5.59/5,46 | 19.8/20.7 |
| *M5005_spy0218* |  | N-acetylmannosamine kinase | 2.21 | Stat | 5.95/6.51 | 24.6/24.6 |
| *M5005_spy0475* |  | PTS system, beta-glucoside-specific IIABC component | 2.17 | Stat | 5.90/8.41 | 17.9/21.1 |
| *M5005_spy0476* | *bglA* | 6-phospho-beta-glucosidase | 2.06/9.58 | Mid/Stat | 5.24/9.58 | 21.0/20.7 |
| *M5005_spy0519* | *agaD* | PTS system, N-acetylgalactosamine-specific IID component | 4.22/2.42 | Mid/Stat | 3.72 (M) | 12.4/2.35 |
| *M5005_spy0520* |  | PTS system, N-acetylgalactosamine-specific IIC component | 6.24 | Stat | NS | 5.5.1/4.22 |
| *M5005_spy0521* | *agaV* | PTS system, N-acetylgalactosamine-specific IIB component | 5.24 | Stat | NS | 3.76/2.90 |
| *M5005_spy0662* | *fruA* | PTS system, fructose-specific IIABC component | 2.50 | Mid | NS | 3.85/0.89 |
| *M5005_spy1056* | *malM* | 4-alpha-glucanotransferase | 2.10 | Stat | NS | NS |
| *M5005_spy1059* | *malF* | Maltose transport system permease protein | 2.43 | Stat | NS | 0.21 (S) |
| *M5005_spy1060* | *malG* | Maltose transport system permease protein | 2.10 | Stat | NS | 0.41 (S) |
| *M5005_spy1062* | *malA* | Maltodextrose utilization protein | 2.63 | Stat | 6.91/9.51 | 26.9/26.7 |
| *M5005_spy1063* | *malD* | Maltodextrin transport system permease protein | 2.55 | Stat | 4.70/2.74 | 8.81/4.55 |
| *M5005_spy1064* | *malC* | Maltose transport system permease protein | 2.40 | Stat | 5.86/2.34 | 6.08/3.90 |
| *M5005_spy1065* | *amyA* | Alpha-amylase | 2.62 | Stat | 8.14/2.32 | 6.39/3.67 |
| *M5005_spy1066* | *amyB* | Neopullulanase/cyclomaltodextrinase/maltogenic alpha-amylase | 3.99 | Stat | 5.38/3.13 | 10.0/5.91 |
| *M5005_spy1067* | *malX* | Maltose/maltodextrin-binding protein | 3.17 | Stat | 6.80/2.14 | 9.14/4.09 |
| *M5005_spy1079* |  | PTS system, cellobiose-specific IIC component | 2.16 | Stat | 8.87/3.11 | 17.2/11.4 |
| *M5005_spy1081* |  | PTS system, cellobiose-specific IIA component | 2.22 | Stat | 7.61/1.84 | 8.97/5.64 |
| *M5005_spy1082* |  | PTS system, cellobiose-specific IIB component | 2.08 | Stat | 8.88 (M) | 13.1/6.71 |
| *M5005_spy1083* |  | PTS system, mannitol (cryptic)-specific IIA component/Transcription antiterminator, BglG family | 3.36/2.11 | Mid/Stat | 7.67/2.49 | 28.6/5.10 |
| *M5005_spy1085* | *bglA.2* | Beta-glucosidase | 2.12 | Stat | NS | 5.20/4.05 |
| *M5005_spy1139* | *nagB* | Glucosamine-6-phosphate isomerase | 2.09 | Stat | NS | 2.04 (M) |
| *M5005_spy1304* | *lacZ* | Beta-galactosidase | 2.69 | Stat | NS | 3.25/2.70 |
| *M5005_spy1308* |  | Sugar-binding protein | 3.54 | Stat | 2.06 (M) | 3.82/3.01 |
| *M5005_spy1309* |  | Sugar transport system permease protein | 5.85 | Stat | 2.92 (S) | 9.12/4.55 |
| *M5005_spy1310* |  | Sugar transport system permease protein | 4.09 | Stat | NS | 3.15/2.39 |
| *M5005_spy1632* | *lacG* | 6-phospho-beta-galactosidase | 2.33 (S) | Mid/Stat | -3.21/8.91 | 28.8 (S) |
| *M5005_spy1633* | *lacE* | PTS system, lactose-specific IIBC component | 2.71 (S) | Mid/Stat | -2.52/7.52 | 16.8 (S) |
| *M5005_spy1634* | *lacF* | PTS system, lactose-specific IIA component | 2.47 (S) | Mid/Stat | -2.71/6.39 | 13.8 (S) |
| *M5005_spy1636* | *lacC.2* | Tagatose-6-phosphate kinase | 2.51 (S) | Mid/Stat | -2.03/8.21 | 14.3 (S) |
| *M5005_spy1637* | *lacB.2* | Galactose-6-phosphate isomerase LacB subunit | 2.37 (S) | Mid/Stat | -3.22/7.56 | 13.9 (S) |
| *M5005_spy1638* | *lacA.2* | Galactose-6-phosphate isomerase LacA subunit | 3.21 (S) | Mid/Stat | -2.97/6.39 | 10.3 (S) |
| *M5005_spy1376* |  | Transaldolase | 2.28 | Stat | 4.20/2.77 | 6.13/5.61 |
| *M5005_spy1379* | *glpF* | Glycerol uptake facilitator protein | 2.02 | Stat | 11.2/2.84 | 21.2/9.56 |
| *M5005_spy1395* | *lacD.1* | Tagatose 1,6-bisphosphate aldolase | 3.88 | Stat | 5.39 (M) | 17.1/7.83 |
| *M5005_spy1396* | *nadE* | Tagatose-6-phosphate kinase | 6.23 | Mid | 6.23 (M) | 9.02/6.69 |
| *M5005_spy1397* | *lacB.1* | Galactose-6-phosphate isomerase subunit lacB | 6.87 | Stat | 9.12 (M) | 20.8/10.2 |
| *M5005_spy1398* | *lacA.1* | Galactose-6-phosphate isomerase subunit lacA | 2.27 | Stat | 7.21/2.80 | 56.8/22.5 |
| *M5005_spy1399* |  | PTS system, galactose-specific IIC component | 4.56 | Stat | 5.96 (M) | 16.5/3.35 |
| *M5005_spy1400* |  | PTS system, galactose-specific IIB component | 3.59 | Stat | 8.25 (M) | 10.1/2.64 |
| *M5005_spy1401* |  | PTS system, galactose-specific IIA component | 3.44 | Stat | 7.13 (M) | 20.2/2.18 |
| *M5005_spy1538* | *pmi* | Mannonse-6-phosphate isomerase | 3.03 | Stat | 9.02 (S) | 24.0 (S) |
| *M5005_spy1542* | *scrA* | PTS system, sucrose-specific IIABC component | 3.23 | Stat | 24.2 (S) | 30.3 (S) |
| *M5005_spy1661* |  | Transaldolase | 2.48 | Stat | 3.35 (M) | 8.94/6.55 |
| *M5005_spy1662* | *ulaA* | Ascorbate-specific PTS system enzyme IIC | 2.01 | Mid | 5.78/2.91 | 12.4/5.71 |
| *M5005_spy1663* |  | PTS system, IIB component | 2.37/5.47 | Mid/Stat | 5.89/4.86 | 23.8/18.4 |
| *M5005_spy1693* | *ptsG* | PTS system, glucose-specific IIABC component | 4.16 | Stat | 8.72 (S) | 2.35 (M) |
| *M5005_spy1744* |  | PTS system, cellobiose-specific IIC component | 3.06 | Stat | 2.07/3.31 | 6.22/5.46 |
| *M5005_spy1745* |  | PTS system, cellobiose-specific IIB component | 2.09/4.28 | Mid/Stat | 4.31/3.56 | 15.9/6.45 |
| *M5005_Spy1746* |  | PTS system, cellobiose-specific IIA component | 3.23 | Stat | 3.03/2.00 | 10.7/4.69 |
| *M5005_spy1783* | *dexS* | Trehalose-6-phosphate hydrolase | 3.21/2.84 | Mid/Stat | NS | 2.93/1.27 |
| *M5005_spy1784* |  | PTS system, trehalose-specific IIBC component | 2.29/4.25 | Mid/Stat | NS | 3.42 (M) |
| **Cell wall/membrane biogenesis** | | |  |  |  |  |
| *M5005_spy0500* |  | N-acetylmuramoyl-L-alanine amidase | 2.98/4.29 | Mid/Stat | NS | 2.33/3.30 |
| *M5005_spy1851* | *hasA* | Hyaluronan synthase | 41.3/49.6 | Mid/Stat | NS | 25.2/73.0 |
| *M5005_spy1852* | *hasB* | UDP-glucose 6-dehydrogenase | 32.8/36.6 | Mid/Stat | NS | 17.7/47.9 |
| *M5005_spy1853* | *hasC* | UTP-glucose-1-phosphate uridylyltransferase | 29.0/24.5 | Mid/Stat | NS | 16.3/30.7 |
| **Cellular processing** | | |  |  |  |  |
| *M5005_spy0241* | *rgpG* | Hypothetical cytosolic protein | 2.02 | Mid | NS | NS |
| **Coenzyme transport and metabolism** | | |  |  |  |  |
| *M5005_spy0860* | *apbE* | Thiamine biosynthesis lipoprotein | 7.31 | Stat | NS | NS |
| *M5005_spy1086* |  | Nicotinamide mononucleotide transporter | 2.08 | Stat | 2.59 (S) | NS |
| **Defense mechanisms/virulence** | | |  |  |  |  |
| *M5005_spy0139* | *nga* | NAD glycohydrolase | 12.1/21.3 | Mid/Stat | 3.56 (S) | 31.2/28.6 |
| *M5005_spy0141* | *slo* | Streptolysin O | 12.3/25.2 | Mid/Stat | 2.73 (S) | 25.3/32.5 |
| *M5005_spy0341* | *spyCEP* | IL-8-degrading proteinase | 17.4/10.1 | Mid/Stat | 8.33/7.43 | 25.5/34.2 |
| *M5005_spy0351* | *spyA* | ADP-ribosyltransferase, C3 family | 3.78/14.7 | Mid/Stat | NS | 5.20/10.3 |
| *M5005_spy0356* | *speJ* | Exotoxin type J precursor | 2.77/3.14 | Mid/Stat | NS | 2.20/5.10 |
| *M5005_spy0561* | *epf* | Putative extracellular matrix binding protein | 14.7/15.3 | Mid/Stat | NS | 17.0/11.1 |
| *M5005_spy0562* | *sagA* | Streptolysin S precursor | 2.31/5.34 | Mid/Stat | 2.68 (M) | 8.35/8.36 |
| *M5005_spy0563* | *sagB* | Streptolysin S biosynthesis protein sagB | 3.21/15.5 | Mid/Stat | 3.65/5.28 | 10.5/19.2 |
| *M5005_spy0564* | *sagC* | Streptolysin S biosynthesis protein sagC | 2.81/8.71 | Mid/Stat | 3.12/4.12 | 8.84/15.3 |
| *M5005_spy0565* | *sagD* | Streptolysin S biosynthesis protein sagD | 4.02/10.0 | Mid/Stat | 3.82/4.60 | 9.79/12.5 |
| *M5005_spy0566* | *sagE* | Streptolysin S putative self-immunity protein sagE | 3.01/14.9 | Mid/Stat | 3.73/5.84 | 10.4/19.3 |
| *M5005_spy0567* | *sagF* | Streptolysin S biosynthesis protein sagF | 2.56/11.2 | Mid/Stat | 3.14/4.65 | 6.15/9.57 |
| *M5005_spy0568* | *sagG* | Streptolysin S export ATP-binding protein sagG | 2.07/6.57 | Mid/Stat | 2.57/3.45 | 5.32/8.30 |
| *M5005_spy0569* | *sagH* | Streptolysin S export transmembrane protein sagH | 2.38/8.12 | Mid/Stat | 3.03/3.93 | 6.50/10.1 |
| *M5005_spy0570* | *sagI* | Streptolysin S export transmembrane protein sagI | 2.15/5.51 | Mid/Stat | 2.23/3.01 | 5.22/6.94 |
| *M5005_spy0571* |  | Endonuclease/exonuclease/phosphatase family protein | 2.73 | Stat | NS | 3.89/3.87 |
| *M5005_spy0667* |  | Exotoxin type C precursor | 41.6/75.9 | Mid/Stat | NS | 40.1/83.4 |
| *M5005_spy0668* | *mac* | IgG-degrading protease of GAS | 38.8/50.2 | Mid/Stat | NS | 30.8/46.4 |
| *M5005_spy0803* | *srtI* | Protein involved in lantibiotic (srt) production | 2.71/2.11 | Mid/Stat | NS | 3.16 (M) |
| *M5005_spy0996* | *speA2* | Exotoxin type A precursor, A2 allele | 7.27/19.5 | Mid/Stat | NS | 4.14/8.65 |
| *M5005_spy1106* | *grab* | Protein G-related alpha 2M-binding protein | 3.57 | Mid | NS | 2.32 (M) |
| *M5005_spy1415* | *sdaD2* | Streptodornase | 2.69/7.86 | Mid/Stat | 2.31 (S) | 2.20/10.1 |
| *M5005_spy1540* | *endoS* | Endo-beta-N-acetylglucosaminidase F2 precursor | 2.61 | Stat | 21.3 (S) | 26.6 (S) |
| *M5005_spy1684* | *ska* | Streptokinase | 3.24/13.3 | Mid/Stat | NS | 2.34/17.6 |
| *M5005_spy1687* | *sclA* | Collagen-like surface protein A | 37.4/17.5 | Mid/Stat | NS | 33.9/41.1 |
| *M5005_spy1688* |  | Immunoglobulin receptor precursor | 2.29 | Mid | 2.29 (M) | 98.3/35.3 |
| *M5005_spy1689* |  | Collagen-like surface protein | 21.4/196 | Mid/Stat | -2.05 (M) | 19.2/453 |
| *M5005_spy1691* |  | Endonuclease/exonuclease/phosphatase family protein | 2.97/4.24 | Mid/Stat | 3.15/2.01 | 7.65 (M) |
| *M5005_spy1714* | *fba* | Fibronectin-binding protein | 4.12 | Stat | 3.23 (S) | 5.01 (S) |
| *M5005_spy1715* | *scpA* | C5a peptidase precursor | 5.13 | Stat | 2.23 (S) | 5.23 (S) |
| *M5005_spy1718* | *sic* | Streptococcal inhibitor of complement | 2.39 | Stat | NS | 4.23 (S) |
| *M5005_spy1735* | *speB* | Cysteine protease | 2.23 | Mid | -5.21 (M) | -5.23 (M) |
| *M5005_spy1738* | *spd* | Streptodornase | 2.24 | Mid | NS | 3.02 (M) |
| **Energy production and conversion** | | |  |  |  |  |
| *M5005_spy0039* | *adh2* | Alcohol/acetaldehyde-CoA dehydrogenase | 2.60/2.23 | Mid/Stat | NS | 11.0 (M) |
| *M5005_spy0126* | *ntpI* | V-type sodium ATP synthase subunit I | 5.08 | Stat | 12.3/0.18 | 4.32/29.2 |
| *M5005_spy0127* | *ntpK* | V-type sodium ATP synthase subunit K | 2.88 | Stat | 11.3/3.11 | 3.58/20.9 |
| *M5005_spy0128* | *ntpE* | V-type sodium ATP synthase subunit E | 2.37 | Stat | 11.0/3.61 | 4.21/17.0 |
| *M5005_spy0129* | *ntpC* | V-type sodium ATP synthase subunit C | 2.45/2.50 | Mid/Stat | 18.9/4.09 | 7.92/26.5 |
| *M5005_spy0130* | *ntpF* | V-type sodium ATP synthase subunit F | 2.31 | Stat | 11.8/3.11 | 3.92/13.6 |
| *M5005_spy0900* |  | Mg2+/citrate complex secondary transporter | 2.49 | Stat | 3.00 (S) | 5.76/7.97 |
| *M5005_spy0903* | *oadB* | Oxaloacetate decarboxylase beta chain | 2.70 | Stat | NS | 2.06 (M) |
| *M5005_spy0906* | *citE* | Citrate lyase beta chain/citryl-CoA lyase subunit | 2.13 | Stat | NS | 2.29 (M) |
| *M5005_spy0907* | *citF* | Citrate lyase alpha chain/citrate CoA-transferase | 2.16 | Stat | NS | 2.52 (M) |
| *M5005_spy0909* | *oadA1* | Oxaloacetate decarboxylase alpha chain | 5.88 | Stat | NS | 2.26/0.40 |
| **Inorganic ion transport and metabolism** | | |  |  |  |  |
| *M5005_spy0543* | *adcA* | High-affinity zinc uptake system protein znuA precursor | 2.28 | Mid | NS | 2.40 (M) |
| *M5005_spy1152* |  | Kup system potassium uptake protein | 2.12 | Mid | NS | NS |
| *M5005_spy1153* |  | Kup system potassium uptake protein | 2.68 | Mid | NS | NS |
| *M5005_spy1161* |  | Formate transporter | 2.79 | Stat | 2.04 (S) | 3.16/2.71 |
| *M5005_spy1403* |  | Copper chaperone | 3.43/3.48 | Mid/Stat | NS | 3.24 (M) |
| *M5005_spy1527* |  | Ferrichrome transport system permease protein | 2.00 | Stat | NS | NS |
| *M5005_spy1711* | *lmb* | Laminin binding protein | 2.00 | Mid | NS | 2.92 (M) |
| **Lipid transport and metabolism** | | |  |  |  |  |
| *M5005_spy0120* | *atoD.2* | Acetate CoA-transferase alpha subunit | 3.82 | Stat | 2.74 (S) | 2.87/6.96 |
| *M5005_spy0359* | *fabG* | 3-ketoacyl-acyl carrier protein reductase | -2.56 | Stat | NS | -2,16 (S) |
| *M5005_spy0535* |  | Acetoin dehydrogenase | 2.02 | Stat | 2.30 (M) | 5.12/1.68 |
| *M5005_spy0687* | *mvaS.1* | Hydroxymethylglutaryl coA synthase | -2.87 | Stat | NS | NS |
| *M5005_spy0902* |  | Acetyl-CoA carboxylase biotin carboxyl carrier protein subunit | 2.40 | Stat | NS | 2.48/0.08 |
| **Nucleotide transport and metabolism** | | |  |  |  |  |
| *M5005_spy0080* |  | Bis(5’-nucleosyl)-tetraphosphatase | -4.90 | Stat | 5.82 (S) | 3.22 (S) |
| *M5005_spy0347* | *nrdF* | Ribonucleotide-diphosphate reductase | -4.03 | Stat | 2.10/3.41 | NS |
| *M5005_spy0639* | *pyrR* | Pyramidine regulatory protein | -6.88 | Stat | NS | -5.82 (S) |
| *M5005_spy0640* | *pyrP* | Uracil permease | -8.72 | Stat | NS | -6.23 (S) |
| *M5005_spy0641* | *pyrB* | Aspartate carbamoyl transferase | -5.51 | Stat | NS | -6.71 (S) |
| *M5005_spy0642* | *carA* | Carbamoyl phosphate synthase | -5.69 | Stat | NS | -5.85 (S) |
| *M5005_spy0643* | *carB* | Carbamoyl phosphate synthase | -5.71 | Stat | NS | -4.82 (S) |
| *M5005_spy0704* | *pyrE* | Orotate phosphoribosyltransferase | -2.82 | Stat | NS | NS |
| *M5005_spy0775* |  | Nucleoside diphosphate kinase | 2.02 | Mid | NS | 2.70 (M) |
| **Phage** | |  |  |  |  |  |
| *M5005_spy0459* |  | Portal protein | 2.26 | Mid | 5.01 (M) | 5.99 (M) |
| *M5005_spy0669* |  | Phage protein | 3.67/2.38 | Mid/Stat | NS | NS |
| *M5005_spy0995* |  | Phage protein | 2.51 | Mid | 2.73/4.82 | 8.54/11.9 |
| *M5005_spy1021* |  | Phage protein | 4.53 | Mid | 3.21 (S) | 13.2/9.11 |
| *M5005_spy1022* |  | Portal protein | 2.53 | Mid | NS | 2.93/2.42 |
| *M5005_spy1173* |  | Phage protein | 5.35 | Mid | 2.88 (M) | 5.08 (M) |
| *M5005_spy1175* |  | Phage protein | 3.40 | Mid | NS | 12.1 (M) |
| *M5005_spy1429* |  | Phage protein | -2.28/-4.08 | Mid/Stat | NS | -2.19 (M) |
| *M5005_spy1430* |  | Phage protein | -2.38/-3.58 | Mid/Stat | NS | NS |
| **Replication, recombination and repair** | | |  |  |  |  |
| *M5005_spy0112* |  | Transposase | 2.11 | Mid | NS | NS |
| *M5005_spy0113* |  | Transposase | 97.5/11.5 | Mid/Stat | NS | 18.0/11.7 |
| *M5005_spy0254* |  | Transposase | 4.50 | Mid | 4.70/2.32 | 6.48/4.07 |
| *M5005_spy1236* | *phr* | Deoxyribodipyrimidine photolyase | -3.83 | Stat | NS | NS |
| *M5005_spy1285* |  | Hypothetical cytosolic protein | 2.04/5.73 | Mid/Stat | NS | 4.40/6.31 |
| *M5005_spy1287* |  | Hypothetical protein | 2.08/7.95 | Mid/Stat | NS | 4.22/6.93 |
| **Transcription** | | |  |  |  |  |
| *M5005_spy0186* |  | Transcriptional regulator | 2.48/2.89 | Mid/Stat | NS | NS |
| *M5005_spy1760* |  | Transcriptional regulator, MutR family | 4.63 | Mid | 8.39 (M) | 39.6/4.59 |
| **Translation** | |  |  |  |  |  |
| *M5005_spy0081* | *tyrS* | Tyrosyl tRNA synthetase | -4.88 | Stat | 5.01 (S) | NS |
| *M5005_spy0705* | *amiC* | Amidase | -2.75 | Stat | NS | NS |
| *M5005_spy0798* |  | IFN-response binding factor 1 | 5.92 | Mid | 3.38 (M) | 4.30 (M) |
| **Unknown** | |  |  |  |  |  |
| *M5005_spy0015* |  | Hypothetical protein | 5.28 | Mid | NS | 14.2/2.18 |
| *M5005_spy0017* | *sibA* | Secreted protein of unknown function | -2.25 | Stat | 2.73 (S) | NS |
| *M5005_spy0115* |  | Hypothetical protein | 63.0/300 | Mid/Stat | NS | 48.3/265 |
| *M5005_spy0140* |  | Hypothetical protein | 2.10/23.1 | Mid/Stat | NS | NS |
| *M5005_spy0142* |  | Hypothetical protein | 313/165 | Mid/Stat | NS | 3.22/9.28 |
| *M5005_spy0143* |  | Hypothetical protein | 171/94.5 | Mid/Stat | NS | 122/112 |
| *M5005_spy0234* |  | Hypothetical protein | 2.28 | Mid | NS | NS |
| *M5005_spy0281* |  | Hypothetical cytosolic protein | 2.78/3.77 | Mid/Stat | NS | 2.33/4.36 |
| *M5005_spy0352* |  | Hypothetical protein | 30.4/45.0 | Mid/Stat | NS | 31.7/69.0 |
| *M5005_spy0353* |  | Hypothetical protein | 2.68/8.17 | Mid/Stat | NS | 4.24/16.8 |
| *M5005_spy0354* |  | Hypothetical protein | 11.2/7.54 | Mid/Stat | NS | 6.30/10.6 |
| *M5005_spy0355* |  | Hypothetical protein | 38.4/81.8 | Mid/Stat | NS | 16.8/128 |
| *M5005_spy0357* |  | Hypothetical protein | 5.12/2.69 | Mid/Stat | NS | 3.14/5.05 |
| *M5005_spy0360* |  | NAD dependent oxidoreductase | -2.56 | Stat | NS | -2.28 (S) |
| *M5005_spy0394* |  | Hypothetical protein | 2.48 | Mid | NS | NS |
| *M5005_spy0401* |  | Hypothetical cytosolic protein | 3.59 | Mid | 4.23 (M) | NS |
| *M5005_spy0404* |  | Hypothetical protein | 2.11 | Mid | NS | 2.35 (M) |
| *M5005_spy0454* |  | Hypothetical protein | 2.78 | Mid | 2.43 (M) | NS |
| *M5005_spy0518* |  | Oligohyaluronate lyase | 2.41/6.17 | Mid/Stat | 2.21/2.12 | 5.71/11.3 |
| *M5005_spy0666* |  | Hypothetical protein | 49.3/36.3 | Mid/Stat | NS | 47.3/33.5 |
| *M5005_spy0773* |  | Hypothetical protein | 7.48/3.49 | Mid/Stat | 5.63 (M) | 14.8/4.99 |
| *M5005_spy0812* |  | Hypothetical protein | 3.35 | Mid | 4.29 (M) | 5.09 (M) |
| *M5005_spy0861* |  | Hypothetical protein | -3.93 | Stat | 5.12 (S) | NS |
| *M5005_spy1114* |  | Hypothetical protein | 2.22 | Mid | NS | NS |
| *M5005_spy1142* |  | Hypothetical protein | 16.1/3.97 | Mid/Stat | NS | 18.4/5.21 |
| *M5005_spy1143* |  | Hypothetical protein | 6.56/2.40 | Mid/Stat | NS | 6.57/4.15 |
| *M5005_spy1144* |  | Hypothetical protein | 3.97/2.44 | Mid/Stat | NS | 3.53/3.09 |
| *M5005_spy1290* |  | Hypothetical protein | 2.11/15.7 | Mid/Stat | NS | 4.96/9.93 |
| *M5005_spy1541* |  | Hypothetical protein | 3.05 | Stat | 27.3 (S) | 33.2 (S) |
| *M5005_spy1556* |  | Hypothetical protein | 5.12/8.80 | Mid/Stat | NS | 4.41/7.24 |
| *M5005_spy1665* |  | Hypothetical protein | 2.36 | Mid | NS | NS |
| *M5005_spy1667* |  | Hypothetical protein | 3.22 | Mid | 3.50 (M) | 4.07 (M) |
| *M5005_spy1703* |  | Hypothetical cytosolic protein | 6.81 | Mid | 4.41 (M) | 8.10 (M) |
| *M5005_spy1731* |  | Hypothetical cytosolic protein | 3.72/4.18 | Mid/Stat | NS | 4.16/38.8 |
| *M5005_spy1750* |  | Hypothetical protein | 3.98/4.50 | Mid/Stat | NS | 3.23/2.18 |
| *M5005_spy1860* |  | Hypothetical protein | -2.80 | Stat | 2.23 (S) | NS |
|  | | |  |  |  |  |

| **M5005 ORF** | **Gene name** | **Putative function of encoded protein** | **Fold-change^1^** | **Time-point** | **Fold-change in strain 2221∆*ccpA*^2^** | **Fold-change in strain 2221∆*covR* ∆*ccpA*** |
| --- | --- | --- | --- | --- | --- | --- |
| **Amino acid transport and metabolism** | | |  |  |  |  |
| *M5005_spy0146* | *metB* | Cystathionine beta-lyase | 2.45 | Mid | NS | 2.90 (M) |
| *M5005_spy1269* | *asnA* | Aspartate—ammonia ligase/asparagine synthetase | 2.35 | Mid | 3.17 (S) | 2.20 (S) |
| *M5005_spy1270* | *arcC* | Carbamate kinase | 4.31/2.35 | Mid/Stat | 372/2.95 | 611/4.60 |
| *M5005_spy1271* |  | Xaa-His dipeptidase | 3.93/2.27 | Mid/Stat | 54.2/2.87 | 111/5.36 |
| *M5005_spy1272* |  | Arginine/ornithine antiporter | 4.15/3.12 | Mid/Stat | 26.4/2.58 | 53.6/4.25 |
| *M5005_spy1273* | *arcB* | Ornithine carbamoyltransferase | 3.52 | Mid | 19.0 (M) | 31.7/2.43 |
| *M5005_spy1274* |  | Acetyltransferase | 2.91 | Mid | 20.3 (M) | 25.6 (M) |
| *M5005_spy1275* | *arcA* | Arginine deiminase | 2.27 | Mid | 22.1 (M) | 28.8 (M) |
| *M5005_spy1704* | *dppA* | Dipeptide transport | -3.13 | Mid | NS | -5.13 (M) |
| *M5005_spy1705* | *dppB* | Dipeptide transport | -2.97 | Mid | NS | -5.83 (M) |
| *M5005_spy1706* | *dppC* | Dipeptide transport | -2.80 | Mid | NS | -5.29 (M) |
| *M5005_spy1707* | *dppD* | Dipeptide transport | -2.71 | Mid | NS | -4.91 (M) |
| *M5005_spy1708* | *dppE* | Dipeptide transport | -2.56 | Mid | NS | -5.26 (M) |
| *M5005_spy1770* | *hutI* | Imidazolonepropionase | 2.70 | Stat | 17.5/3.84 | 60.4/13.1 |
| *M5005_spy1771* | *hutU* | Urocanate hydratase | 2.30 | Stat | 3.40 (S) | 4.62/4.07 |
| *M5005_spy1773* |  | Formiminotetrahydrofolate cyclodeaminase | 2.40 | Stat | 3.10 (S) | 2.26/3.22 |
| *M5005_spy1774* | *fhs.2* | Formate—tetrahydrofolate ligase | 2.60 | Stat | 2.70 (S) | 2.74/2.29 |
| *M5005_spy1775* |  | Hypothetical cytosolic protein | 2.90 | Stat | 6.37 (S) | 16.4/6.64 |
| *M5005_spy1776* |  | Amino acid permease | 2.30 | Stat | 4.10 (S) | 2.50/3.62 |
| *M5005_spy1777* | *hutH* | Histidine ammonia-lyase | 2.10 | Stat | 2.30 (S) | 4.60/4.74 |
| *M5005_spy1778* | *hutG* | Formiminoglutamase | 2.30 | Stat | 3.10 (S) | 2.29 (S) |
| **Carbohydrate transport and metabolism** | | |  |  |  |  |
| *M5005_spy0151* | *ulaD* | 3-keto-L-gulonate-6-phosphate decarboxylase | 3.37 | Mid | 2.09 (M) | 3.41 (M) |
| *M5005_spy0212* |  | N-acetylmannosamine-6-phosphate 2-epimerase | 5.57 | Stat | 15.17/5.79 | 28.0/22.2 |
| *M5005_spy0213* |  | N-acetylneuraminate-binding protein | 5.36 | Stat | 12.4/7.04 | 42.6/23.1 |
| *M5005_spy0214* |  | N-acetylneuraminate transport system permease protein | 6.28 | Stat | 9.92/11.4 | 34.2/36.5 |
| *M5005_spy0215* |  | N-acetylneuraminate transport system permease protein | 7.16/2.51 | Mid/Stat | 28.5/4.73 | 142/18.8 |
| *M5005_spy0216* |  | Hypothetical protein | 3.63/4.84 | Mid/Stat | 15.2/6.13 | 136/55.3 |
| *M5005_spy0217* | *nanH* | N-acetylneuraminate lyase | 2.59/3.05 | Mid/Stat | 5.59/5,46 | 19.8/20.7 |
| *M5005_spy0218* |  | N-acetylmannosamine kinase | 2.21 | Stat | 5.95/6.51 | 24.6/24.6 |
| *M5005_spy0475* |  | PTS system, beta-glucoside-specific IIABC component | 2.17 | Stat | 5.90/8.41 | 17.9/21.1 |
| *M5005_spy0476* | *bglA* | 6-phospho-beta-glucosidase | 2.06/9.58 | Mid/Stat | 5.24/9.58 | 21.0/20.7 |
| *M5005_spy0519* | *agaD* | PTS system, N-acetylgalactosamine-specific IID component | 4.22/2.42 | Mid/Stat | 3.72 (M) | 12.4/2.35 |
| *M5005_spy0520* |  | PTS system, N-acetylgalactosamine-specific IIC component | 6.24 | Stat | NS | 5.5.1/4.22 |
| *M5005_spy0521* | *agaV* | PTS system, N-acetylgalactosamine-specific IIB component | 5.24 | Stat | NS | 3.76/2.90 |
| *M5005_spy0662* | *fruA* | PTS system, fructose-specific IIABC component | 2.50 | Mid | NS | 3.85/0.89 |
| *M5005_spy1056* | *malM* | 4-alpha-glucanotransferase | 2.10 | Stat | NS | NS |
| *M5005_spy1059* | *malF* | Maltose transport system permease protein | 2.43 | Stat | NS | 0.21 (S) |
| *M5005_spy1060* | *malG* | Maltose transport system permease protein | 2.10 | Stat | NS | 0.41 (S) |
| *M5005_spy1062* | *malA* | Maltodextrose utilization protein | 2.63 | Stat | 6.91/9.51 | 26.9/26.7 |
| *M5005_spy1063* | *malD* | Maltodextrin transport system permease protein | 2.55 | Stat | 4.70/2.74 | 8.81/4.55 |
| *M5005_spy1064* | *malC* | Maltose transport system permease protein | 2.40 | Stat | 5.86/2.34 | 6.08/3.90 |
| *M5005_spy1065* | *amyA* | Alpha-amylase | 2.62 | Stat | 8.14/2.32 | 6.39/3.67 |
| *M5005_spy1066* | *amyB* | Neopullulanase/cyclomaltodextrinase/maltogenic alpha-amylase | 3.99 | Stat | 5.38/3.13 | 10.0/5.91 |
| *M5005_spy1067* | *malX* | Maltose/maltodextrin-binding protein | 3.17 | Stat | 6.80/2.14 | 9.14/4.09 |
| *M5005_spy1079* |  | PTS system, cellobiose-specific IIC component | 2.16 | Stat | 8.87/3.11 | 17.2/11.4 |
| *M5005_spy1081* |  | PTS system, cellobiose-specific IIA component | 2.22 | Stat | 7.61/1.84 | 8.97/5.64 |
| *M5005_spy1082* |  | PTS system, cellobiose-specific IIB component | 2.08 | Stat | 8.88 (M) | 13.1/6.71 |
| *M5005_spy1083* |  | PTS system, mannitol (cryptic)-specific IIA component/Transcription antiterminator, BglG family | 3.36/2.11 | Mid/Stat | 7.67/2.49 | 28.6/5.10 |
| *M5005_spy1085* | *bglA.2* | Beta-glucosidase | 2.12 | Stat | NS | 5.20/4.05 |
| *M5005_spy1139* | *nagB* | Glucosamine-6-phosphate isomerase | 2.09 | Stat | NS | 2.04 (M) |
| *M5005_spy1304* | *lacZ* | Beta-galactosidase | 2.69 | Stat | NS | 3.25/2.70 |
| *M5005_spy1308* |  | Sugar-binding protein | 3.54 | Stat | 2.06 (M) | 3.82/3.01 |
| *M5005_spy1309* |  | Sugar transport system permease protein | 5.85 | Stat | 2.92 (S) | 9.12/4.55 |
| *M5005_spy1310* |  | Sugar transport system permease protein | 4.09 | Stat | NS | 3.15/2.39 |
| *M5005_spy1376* |  | Transaldolase | 2.28 | Stat | 4.20/2.77 | 6.13/5.61 |
| *M5005_spy1379* | *glpF* | Glycerol uptake facilitator protein | 2.02 | Stat | 11.2/2.84 | 21.2/9.56 |
| *M5005_spy1395* | *lacD.1* | Tagatose 1,6-bisphosphate aldolase | 3.88 | Stat | 5.39 (M) | 17.1/7.83 |
| *M5005_spy1396* | *nadE* | Tagatose-6-phosphate kinase | 6.23 | Mid | 6.23 (M) | 9.02/6.69 |
| *M5005_spy1397* | *lacB.1* | Galactose-6-phosphate isomerase subunit lacB | 6.87 | Stat | 9.12 (M) | 20.8/10.2 |
| *M5005_spy1398* | *lacA.1* | Galactose-6-phosphate isomerase subunit lacA | 2.27 | Stat | 7.21/2.80 | 56.8/22.5 |
| *M5005_spy1399* |  | PTS system, galactose-specific IIC component | 4.56 | Stat | 5.96 (M) | 16.5/3.35 |
| *M5005_spy1400* |  | PTS system, galactose-specific IIB component | 3.59 | Stat | 8.25 (M) | 10.1/2.64 |
| *M5005_spy1401* |  | PTS system, galactose-specific IIA component | 3.44 | Stat | 7.13 (M) | 20.2/2.18 |
| *M5005_spy1538* | *pmi* | Mannonse-6-phosphate isomerase | 3.03 | Stat | 9.02 (S) | 24.0 (S) |
| *M5005_spy1542* | *scrA* | PTS system, sucrose-specific IIABC component | 3.23 | Stat | 24.2 (S) | 30.3 (S) |
| *M5005_spy1661* |  | Transaldolase | 2.48 | Stat | 3.35 (M) | 8.94/6.55 |
| *M5005_spy1662* | *ulaA* | Ascorbate-specific PTS system enzyme IIC | 2.01 | Mid | 5.78/2.91 | 12.4/5.71 |
| *M5005_spy1663* |  | PTS system, IIB component | 2.37/5.47 | Mid/Stat | 5.89/4.86 | 23.8/18.4 |
| *M5005_spy1693* | *ptsG* | PTS system, glucose-specific IIABC component | 4.16 | Stat | 8.72 (S) | 2.35 (M) |
| *M5005_spy1744* |  | PTS system, cellobiose-specific IIC component | 3.06 | Stat | 2.07/3.31 | 6.22/5.46 |
| *M5005_spy1745* |  | PTS system, cellobiose-specific IIB component | 2.09/4.28 | Mid/Stat | 4.31/3.56 | 15.9/6.45 |
| *M5005_Spy1746* |  | PTS system, cellobiose-specific IIA component | 3.23 | Stat | 3.03/2.00 | 10.7/4.69 |
| *M5005_spy1783* | *dexS* | Trehalose-6-phosphate hydrolase | 3.21/2.84 | Mid/Stat | NS | 2.93/1.27 |
| *M5005_spy1784* |  | PTS system, trehalose-specific IIBC component | 2.29/4.25 | Mid/Stat | NS | 3.42 (M) |
| **Cell wall/membrane biogenesis** | | |  |  |  |  |
| *M5005_spy0500* |  | N-acetylmuramoyl-L-alanine amidase | 2.98/4.29 | Mid/Stat | NS | 2.33/3.30 |
| *M5005_spy1851* | *hasA* | Hyaluronan synthase | 41.3/49.6 | Mid/Stat | NS | 25.2/73.0 |
| *M5005_spy1852* | *hasB* | UDP-glucose 6-dehydrogenase | 32.8/36.6 | Mid/Stat | NS | 17.7/47.9 |
| *M5005_spy1853* | *hasC* | UTP-glucose-1-phosphate uridylyltransferase | 29.0/24.5 | Mid/Stat | NS | 16.3/30.7 |
| **Cellular processing** | | |  |  |  |  |
| *M5005_spy0241* | *rgpG* | Hypothetical cytosolic protein | 2.02 | Mid | NS | NS |
| **Coenzyme transport and metabolism** | | |  |  |  |  |
| *M5005_spy0860* | *apbE* | Thiamine biosynthesis lipoprotein | 7.31 | Stat | NS | NS |
| *M5005_spy1086* |  | Nicotinamide mononucleotide transporter | 2.08 | Stat | 2.59 (S) | NS |
| **Defense mechanisms/virulence** | | |  |  |  |  |
| *M5005_spy0139* | *nga* | NAD glycohydrolase | 12.1/21.3 | Mid/Stat | 3.56 (S) | 31.2/28.6 |
| *M5005_spy0141* | *slo* | Streptolysin O | 12.3/25.2 | Mid/Stat | 2.73 (S) | 25.3/32.5 |
| *M5005_spy0341* | *spyCEP* | IL-8-degrading proteinase | 17.4/10.1 | Mid/Stat | 8.33/7.43 | 25.5/34.2 |
| *M5005_spy0351* | *spyA* | ADP-ribosyltransferase, C3 family | 3.78/14.7 | Mid/Stat | NS | 5.20/10.3 |
| *M5005_spy0356* | *speJ* | Exotoxin type J precursor | 2.77/3.14 | Mid/Stat | NS | 2.20/5.10 |
| *M5005_spy0561* | *epf* | Putative extracellular matrix binding protein | 14.7/15.3 | Mid/Stat | NS | 17.0/11.1 |
| *M5005_spy0562* | *sagA* | Streptolysin S precursor | 2.31/5.34 | Mid/Stat | 2.68 (M) | 8.35/8.36 |
| *M5005_spy0563* | *sagB* | Streptolysin S biosynthesis protein sagB | 3.21/15.5 | Mid/Stat | 3.65/5.28 | 10.5/19.2 |
| *M5005_spy0564* | *sagC* | Streptolysin S biosynthesis protein sagC | 2.81/8.71 | Mid/Stat | 3.12/4.12 | 8.84/15.3 |
| *M5005_spy0565* | *sagD* | Streptolysin S biosynthesis protein sagD | 4.02/10.0 | Mid/Stat | 3.82/4.60 | 9.79/12.5 |
| *M5005_spy0566* | *sagE* | Streptolysin S putative self-immunity protein sagE | 3.01/14.9 | Mid/Stat | 3.73/5.84 | 10.4/19.3 |
| *M5005_spy0567* | *sagF* | Streptolysin S biosynthesis protein sagF | 2.56/11.2 | Mid/Stat | 3.14/4.65 | 6.15/9.57 |
| *M5005_spy0568* | *sagG* | Streptolysin S export ATP-binding protein sagG | 2.07/6.57 | Mid/Stat | 2.57/3.45 | 5.32/8.30 |
| *M5005_spy0569* | *sagH* | Streptolysin S export transmembrane protein sagH | 2.38/8.12 | Mid/Stat | 3.03/3.93 | 6.50/10.1 |
| *M5005_spy0570* | *sagI* | Streptolysin S export transmembrane protein sagI | 2.15/5.51 | Mid/Stat | 2.23/3.01 | 5.22/6.94 |
| *M5005_spy0571* |  | Endonuclease/exonuclease/phosphatase family protein | 2.73 | Stat | NS | 3.89/3.87 |
| *M5005_spy0667* |  | Exotoxin type C precursor | 41.6/75.9 | Mid/Stat | NS | 40.1/83.4 |
| *M5005_spy0668* | *mac* | IgG-degrading protease of GAS | 38.8/50.2 | Mid/Stat | NS | 30.8/46.4 |
| *M5005_spy0803* | *srtI* | Protein involved in lantibiotic (srt) production | 2.71/2.11 | Mid/Stat | NS | 3.16 (M) |
| *M5005_spy0996* | *speA2* | Exotoxin type A precursor, A2 allele | 7.27/19.5 | Mid/Stat | NS | 4.14/8.65 |
| *M5005_spy1106* | *grab* | Protein G-related alpha 2M-binding protein | 3.57 | Mid | NS | 2.32 (M) |
| *M5005_spy1415* | *sdaD2* | Streptodornase | 2.69/7.86 | Mid/Stat | NS | 2.20/10.1 |
| *M5005_spy1540* | *endoS* | Endo-beta-N-acetylglucosaminidase F2 precursor | 2.61 | Stat | 21.3 (S) | 26.6 (S) |
| *M5005_spy1684* | *ska* | Streptokinase | 3.24/13.3 | Mid/Stat | NS | 2.34/17.6 |
| *M5005_spy1687* | *sclA* | Collagen-like surface protein A | 37.4/17.5 | Mid/Stat | NS | 33.9/41.1 |
| *M5005_spy1688* |  | Immunoglobulin receptor precursor | 2.29 | Mid | 2.29 (M) | 98.3/35.3 |
| *M5005_spy1689* |  | Collagen-like surface protein | 21.4/196 | Mid/Stat | -2.05 (M) | 19.2/453 |
| *M5005_spy1691* |  | Endonuclease/exonuclease/phosphatase family protein | 2.97/4.24 | Mid/Stat | 3.15/2.01 | 7.65 (M) |
| *M5005_spy1714* | *fba* | Fibronectin-binding protein | 4.12 | Stat | 3.23 (S) | 5.01 (S) |
| *M5005_spy1715* | *scpA* | C5a peptidase precursor | 5.13 | Stat | 2.23 (S) | 5.23 (S) |
| *M5005_spy1718* | *sic* | Streptococcal inhibitor of complement | 2.39 | Stat | NS | 4.23 (S) |
| *M5005_spy1735* | *speB* | Cysteine protease | 2.23 | Mid | -5.21 (M) | -5.23 (M) |
| *M5005_spy1738* | *spd* | Streptodornase | 2.24 | Mid | NS | 3.02 (M) |
| **Energy production and conversion** | | |  |  |  |  |
| *M5005_spy0039* | *adh2* | Alcohol/acetaldehyde-CoA dehydrogenase | 2.60/2.23 | Mid/Stat | NS | 11.0 (M) |
| *M5005_spy0126* | *ntpI* | V-type sodium ATP synthase subunit I | 5.08 | Stat | 12.3/0.18 | 4.32/29.2 |
| *M5005_spy0127* | *ntpK* | V-type sodium ATP synthase subunit K | 2.88 | Stat | 11.3/3.11 | 3.58/20.9 |
| *M5005_spy0128* | *ntpE* | V-type sodium ATP synthase subunit E | 2.37 | Stat | 11.0/3.61 | 4.21/17.0 |
| *M5005_spy0129* | *ntpC* | V-type sodium ATP synthase subunit C | 2.45/2.50 | Mid/Stat | 18.9/4.09 | 7.92/26.5 |
| *M5005_spy0130* | *ntpF* | V-type sodium ATP synthase subunit F | 2.31 | Stat | 11.8/3.11 | 3.92/13.6 |
| *M5005_spy0900* |  | Mg2+/citrate complex secondary transporter | 2.49 | Stat | 3.00 (S) | 5.76/7.97 |
| *M5005_spy0903* | *oadB* | Oxaloacetate decarboxylase beta chain | 2.70 | Stat | NS | 2.06 (M) |
| *M5005_spy0906* | *citE* | Citrate lyase beta chain/citryl-CoA lyase subunit | 2.13 | Stat | NS | 2.29 (M) |
| *M5005_spy0907* | *citF* | Citrate lyase alpha chain/citrate CoA-transferase | 2.16 | Stat | NS | 2.52 (M) |
| *M5005_spy0909* | *oadA1* | Oxaloacetate decarboxylase alpha chain | 5.88 | Stat | NS | 2.26/0.40 |
| **Inorganic ion transport and metabolism** | | |  |  |  |  |
| *M5005_spy0543* | *adcA* | High-affinity zinc uptake system protein znuA precursor | 2.28 | Mid | NS | 2.40 (M) |
| *M5005_spy1152* |  | Kup system potassium uptake protein | 2.12 | Mid | NS | NS |
| *M5005_spy1153* |  | Kup system potassium uptake protein | 2.68 | Mid | NS | NS |
| *M5005_spy1161* |  | Formate transporter | 2.79 | Stat | 2.04 (S) | 3.16/2.71 |
| *M5005_spy1403* |  | Copper chaperone | 3.43/3.48 | Mid/Stat | NS | 3.24 (M) |
| *M5005_spy1527* |  | Ferrichrome transport system permease protein | 2.00 | Stat | NS | NS |
| *M5005_spy1711* | *lmb* | Laminin binding protein | 2.00 | Mid | NS | 2.92 (M) |
| **Lipid transport and metabolism** | | |  |  |  |  |
| *M5005_spy0120* | *atoD.2* | Acetate CoA-transferase alpha subunit | 3.82 | Stat | 2.74 (S) | 2.87/6.96 |
| *M5005_spy0359* | *fabG* | 3-ketoacyl-acyl carrier protein reductase | -2.56 | Stat | NS | -2,16 (S) |
| *M5005_spy0535* |  | Acetoin dehydrogenase | 2.02 | Stat | 2.30 (M) | 5.12/1.68 |
| *M5005_spy0687* | *mvaS.1* | Hydroxymethylglutaryl coA synthase | -2.87 | Stat | NS | NS |
| *M5005_spy0902* |  | Acetyl-CoA carboxylase biotin carboxyl carrier protein subunit | 2.40 | Stat | NS | 2.48/0.08 |
| **Nucleotide transport and metabolism** | | |  |  |  |  |
| *M5005_spy0080* |  | Bis(5’-nucleosyl)-tetraphosphatase | -4.90 | Stat | 5.82 (S) | 3.22 (S) |
| *M5005_spy0347* | *nrdF* | Ribonucleotide-diphosphate reductase | -4.03 | Stat | 2.10/3.41 | NS |
| *M5005_spy0639* | *pyrR* | Pyramidine regulatory protein | -6.88 | Stat | NS | -5.82 (S) |
| *M5005_spy0640* | *pyrP* | Uracil permease | -8.72 | Stat | NS | -6.23 (S) |
| *M5005_spy0641* | *pyrB* | Aspartate carbamoyl transferase | -5.51 | Stat | NS | -6.71 (S) |
| *M5005_spy0642* | *carA* | Carbamoyl phosphate synthase | -5.69 | Stat | NS | -5.85 (S) |
| *M5005_spy0643* | *carB* | Carbamoyl phosphate synthase | -5.71 | Stat | NS | -4.82 (S) |
| *M5005_spy0704* | *pyrE* | Orotate phosphoribosyltransferase | -2.82 | Stat | NS | NS |
| *M5005_spy0775* |  | Nucleoside diphosphate kinase | 2.02 | Mid | NS | 2.70 (M) |
| **Phage** | |  |  |  |  |  |
| *M5005_spy0459* |  | Portal protein | 2.26 | Mid | 5.01 (M) | 5.99 (M) |
| *M5005_spy0669* |  | Phage protein | 3.67/2.38 | Mid/Stat | NS | NS |
| *M5005_spy0995* |  | Phage protein | 2.51 | Mid | 2.73/4.82 | 8.54/11.9 |
| *M5005_spy1021* |  | Phage protein | 4.53 | Mid | 3.21 (S) | 13.2/9.11 |
| *M5005_spy1022* |  | Portal protein | 2.53 | Mid | NS | 2.93/2.42 |
| *M5005_spy1173* |  | Phage protein | 5.35 | Mid | 2.88 (M) | 5.08 (M) |
| *M5005_spy1175* |  | Phage protein | 3.40 | Mid | NS | 12.1 (M) |
| *M5005_spy1429* |  | Phage protein | -2.28/-4.08 | Mid/Stat | NS | -2.19 (M) |
| *M5005_spy1430* |  | Phage protein | -2.38/-3.58 | Mid/Stat | NS | NS |
| **Replication, recombination and repair** | | |  |  |  |  |
| *M5005_spy0112* |  | Transposase | 2.11 | Mid | NS | NS |
| *M5005_spy0113* |  | Transposase | 97.5/11.5 | Mid/Stat | NS | 18.0/11.7 |
| *M5005_spy0254* |  | Transposase | 4.50 | Mid | 4.70/2.32 | 6.48/4.07 |
| *M5005_spy1236* | *phr* | Deoxyribodipyrimidine photolyase | -3.83 | Stat | NS | NS |
| *M5005_spy1285* |  | Hypothetical cytosolic protein | 2.04/5.73 | Mid/Stat | NS | 4.40/6.31 |
| *M5005_spy1287* |  | Hypothetical protein | 2.08/7.95 | Mid/Stat | NS | 4.22/6.93 |
| **Transcription** | | |  |  |  |  |
| *M5005_spy0186* |  | Transcriptional regulator | 2.48/2.89 | Mid/Stat | NS | NS |
| *M5005_spy1760* |  | Transcriptional regulator, MutR family | 4.63 | Mid | 8.39 (M) | 39.6/4.59 |
| **Translation** | |  |  |  |  |  |
| *M5005_spy0081* | *tyrS* | Tyrosyl tRNA synthetase | -4.88 | Stat | 5.01 (S) | NS |
| *M5005_spy0705* | *amiC* | Amidase | -2.75 | Stat | NS | NS |
| *M5005_spy0798* |  | IFN-response binding factor 1 | 5.92 | Mid | 3.38 (M) | 4.30 (M) |
| **Unknown** | |  |  |  |  |  |
| *M5005_spy0015* |  | Hypothetical protein | 5.28 | Mid | NS | 14.2/2.18 |
| *M5005_spy0017* | *sibA* | Secreted protein of unknown function | -2.25 | Stat | 2.73 (S) | NS |
| *M5005_spy0115* |  | Hypothetical protein | 63.0/300 | Mid/Stat | NS | 48.3/265 |
| *M5005_spy0140* |  | Hypothetical protein | 2.10/23.1 | Mid/Stat | NS | NS |
| *M5005_spy0142* |  | Hypothetical protein | 313/165 | Mid/Stat | NS | 3.22/9.28 |
| *M5005_spy0143* |  | Hypothetical protein | 171/94.5 | Mid/Stat | NS | 122/112 |
| *M5005_spy0234* |  | Hypothetical protein | 2.28 | Mid | NS | NS |
| *M5005_spy0281* |  | Hypothetical cytosolic protein | 2.78/3.77 | Mid/Stat | NS | 2.33/4.36 |
| *M5005_spy0352* |  | Hypothetical protein | 30.4/45.0 | Mid/Stat | NS | 31.7/69.0 |
| *M5005_spy0353* |  | Hypothetical protein | 2.68/8.17 | Mid/Stat | NS | 4.24/16.8 |
| *M5005_spy0354* |  | Hypothetical protein | 11.2/7.54 | Mid/Stat | NS | 6.30/10.6 |
| *M5005_spy0355* |  | Hypothetical protein | 38.4/81.8 | Mid/Stat | NS | 16.8/128 |
| *M5005_spy0357* |  | Hypothetical protein | 5.12/2.69 | Mid/Stat | NS | 3.14/5.05 |
| *M5005_spy0360* |  | NAD dependent oxidoreductase | -2.56 | Stat | NS | -2.28 (S) |
| *M5005_spy0394* |  | Hypothetical protein | 2.48 | Mid | NS | NS |
| *M5005_spy0401* |  | Hypothetical cytosolic protein | 3.59 | Mid | 4.23 (M) | NS |
| *M5005_spy0404* |  | Hypothetical protein | 2.11 | Mid | NS | 2.35 (M) |
| *M5005_spy0454* |  | Hypothetical protein | 2.78 | Mid | 2.43 (M) | NS |
| *M5005_spy0518* |  | Oligohyaluronate lyase | 2.41/6.17 | Mid/Stat | 2.21/2.12 | 5.71/11.3 |
| *M5005_spy0666* |  | Hypothetical protein | 49.3/36.3 | Mid/Stat | NS | 47.3/33.5 |
| *M5005_spy0773* |  | Hypothetical protein | 7.48/3.49 | Mid/Stat | 5.63 (M) | 14.8/4.99 |
| *M5005_spy0812* |  | Hypothetical protein | 3.35 | Mid | 4.29 (M) | 5.09 (M) |
| *M5005_spy0861* |  | Hypothetical protein | -3.93 | Stat | 5.12 (S) | NS |
| *M5005_spy1114* |  | Hypothetical protein | 2.22 | Mid | NS | NS |
| *M5005_spy1142* |  | Hypothetical protein | 16.1/3.97 | Mid/Stat | NS | 18.4/5.21 |
| *M5005_spy1143* |  | Hypothetical protein | 6.56/2.40 | Mid/Stat | NS | 6.57/4.15 |
| *M5005_spy1144* |  | Hypothetical protein | 3.97/2.44 | Mid/Stat | NS | 3.53/3.09 |
| *M5005_spy1290* |  | Hypothetical protein | 2.11/15.7 | Mid/Stat | NS | 4.96/9.93 |
| *M5005_spy1541* |  | Hypothetical protein | 3.05 | Stat | 27.3 (S) | 33.2 (S) |
| *M5005_spy1556* |  | Hypothetical protein | 5.12/8.80 | Mid/Stat | NS | 4.41/7.24 |
| *M5005_spy1665* |  | Hypothetical protein | 2.36 | Mid | NS | NS |
| *M5005_spy1667* |  | Hypothetical protein | 3.22 | Mid | 3.50 (M) | 4.07 (M) |
| *M5005_spy1703* |  | Hypothetical cytosolic protein | 6.81 | Mid | 4.41 (M) | 8.10 (M) |
| *M5005_spy1731* |  | Hypothetical cytosolic protein | 3.72/4.18 | Mid/Stat | NS | 4.16/38.8 |
| *M5005_spy1750* |  | Hypothetical protein | 3.98/4.50 | Mid/Stat | NS | 3.23/2.18 |
| *M5005_spy1860* |  | Hypothetical protein | -2.80 | Stat | 2.23 (S) | NS |

^1^positive numbers indicated increased transcript levels in mutant strain, whereas negative numbers indicate higher transcript levels in wild-type strain

^2^NS = no significant difference in transcript level between wild-type and isogenic mutant strain. If 2 numbers are listed then transcript levels were significantly different in both the mid-exponential and stationary growth phases. If 1 number is listed then the transcript level was only significantly different at one growth phase which is indicated by (M) for mid-exponential and (S) for stationary.
