# Supplementary material for: A Combination of Independent Transcriptional Regulators Shapes Bacterial Virulence Gene Expression during Infection
Source: PLoS Pathog. 2010 Mar 19;6(3):e1000817. doi: 10.1371/journal.ppat.1000817 (PMC2841617; doi:10.1371/journal.ppat.1000817)
Supplement: Table S4 — Genes with altered transcript levels in strain 2221ΔcovRΔccpA vs. wild-type (1.19 MB DOC) [file ppat.1000817.s010.doc]

**Table S4 Genes with altered transcript levels in strain 2221*ΔcovRΔccpA* vs. wild-type.**

| **M5005 ORF** | **Gene name** | **Putative function of encoded protein** | **Fold-change^1^** | **Time-point** | **Fold-change in strain 2221∆*ccpA*^2^** | **Fold-change in strain 2221∆*covR*** |
| --- | --- | --- | --- | --- | --- | --- |
| **Amino acid transport and metabolism** | | |  |  |  |  |
| *M5005_spy0146* | *metB* | Cystathionine beta-lyase | 2.90 | Mid | NS | 2.45 (M) |
| *M5005_spy0157* | *opuAA* | Glycine betaine transport ATP binding protein | -4.91 | Mid | NS | NS |
| *M5005_spy0158* | *opuABC* | Glycine betaine binding protein | -5.21 | Mid | NS | NS |
| *M5005_spy0217* | *nanH* | N-acetylneuraminate lyase | 19.8/20.7 | Mid/Stat | 5.59/5.46 | 2.59/3.05 |
| *M5005_spy0834* |  | Zn-dependent alcohol dehydrogenases and related dehydrogenases | 15.0/3.22 | Mid/Stat | 2.44 (M) | NS |
| *M5005_spy0855* | *proV* | Glycine betaine transport ATP-binding protein | 2.32 | Mid | NS | NS |
| *M5005_spy0856* |  | Glycine betaine transport system permease protein | 2.08 | Mid | NS | NS |
| *M5005_spy0982* |  | Histidine binding protein | -2.91 | Mid | NS | NS |
| *M5005_spy0983* |  | Histidine transport ATP binding protein | -4.51 | Mid | NS | NS |
| *M5005_spy0984* |  | Histidine transport system permease | -3.82 | Mid | NS | NS |
| *M5005_spy1094* |  | Transporter, MFS superfamily | 2.53 | Mid | NS | NS |
| *M5005_spy1181* |  | Major tail protein | 4.30/3.31 | Mid/Stat | 3.14/2.08 | NS |
| *M5005_spy1237* | *artP* | Arginine transport ATP-binding protein | 5.40 | Stat | 8.80 (S) | NS |
| *M5005_spy1238* | *artQ* | Arginine transport system permease protein | 4.10 | Stat | 12.2 (S) | NS |
| *M5005_spy1269* | *asnA* | Aspartate—ammonia ligase/asparagine synthetase | 2.20 | Stat | 3.17 (S) | 2.35 (M) |
| *M5005_spy1270* | *arcC* | Carbamate kinase | 611/4.60 | Mid/Stat | 372/2.95 | 4.31/2.35 |
| *M5005_spy1271* |  | Xaa-His dipeptidase | 111/5.36 | Mid/Stat | 54.2/2.87 | 3.93/2.27 |
| *M5005_spy1272* |  | Arginine/ornithine antiporter | 53.9/4.25 | Mid/Stat | 26.4/2.58 | NS |
| *M5005_spy1273* | *arcB* | Ornithine carbamoyltransferase | 31.7/2.43 | Mid/Stat | 19.0 (M) | 3.52 (M) |
| *M5005_spy1274* |  | Acetyltransferase | 25.6 | Mid | 20.3 (M) | 2.91 (M) |
| *M5005_spy1275* | *arcA* | Arginine deiminase | 28.8 | Mid | 22.1 (M) | 2.27 (M) |
| *M5005_spy1704* | *dppA* | Dipeptide transport | -5.13 | Mid | NS | -3.13 (M) |
| *M5005_spy1705* | *dppB* | Dipeptide transport | -5.83 | Mid | NS | -2.97 (M) |
| *M5005_spy1706* | *dppC* | Dipeptide transport | -5.29 | Mid | NS | -2.80 (M) |
| *M5005_spy1707* | *dppD* | Dipeptide transport | -4.91 | Mid | NS | -2.71 (M) |
| *M5005_spy1708* | *dppE* | Dipeptide transport | -5.26 | Mid | NS | -2.56 (M) |
| *M5005_spy1710* |  | Streptococcal histidine triad protein | 2.28 | Mid | NS | NS |
| *M5005_spy1758* |  | Dipeptidase B | 12.7/2.31 | Mid/Stat | 3.08 (M) | NS |
| *M5005_spy1770* | *hutI* | Imidazolonepropionase | 60.4/13.1 | Mid/Stat | 17.5/3.84 | NS |
| *M5005_spy1771* | *hutU* | Urocanate hydratase | 4.62/4.07 | Mid/Stat | NS | NS |
| *M5005_spy1772* |  | Glutamate formiminotransferase | 9.25/10.9 | Mid/Stat | NS | NS |
| *M5005_spy1773* |  | Formiminotetrahydrofolate cyclodeaminase | 2.26/3.22 | Mid/Stat | NS | 2.40 (S) |
| *M5005_spy1774* |  | Formate—tetrahydrofolate ligase | 2.74/2.29 | Mid/Stat | 2.70 (S) | 2.60 (S) |
| *M5005-_spy1775* |  | Hypothetical cytosolic protein | 16.4/6.64 | Mid/Stat | 6.37 (S) | 2.90 (S) |
| *M5005_spy1776* |  | Amino acid permease | 2.50/3.62 | Mid/Stat | NS | NS |
| *M5005_spy1777* | *hutH* | Histidine ammonia-lyase | 4.60/4.71 | Mid/Stat | 2.30 (S) | 2.10 (S) |
| *M5005_spy1778* | *hutG* | Formiminoglutamase | 2.29 | Stat | 3.10 (S) | 2.30 (S) |
| *M5005_spy1841* | *sdhB* | L-serine dehydratase | -7.65 | Mid | NS | NS |
| **Carbohydrate transport and metabolism** | | |  |  |  |  |
| *M5005_spy0151* |  | 3-keto-L-gulonate-6-phosphate decarboxylase | 3.41 | Mid | 2.09 (M) | 3.37 (M) |
| *M5005_spy0152* |  | L-xylulose 5-phosphate 3-epimerase | 10.4/4.36 | Mid/Stat | NS | NS |
| *M5005_spy0153* | *araD* | L-ribulose-5-phosphate 4-epimerase | 2.83 | Mid | NS | NS |
| *M5005_spy0212* |  | N-acetylmannosamine-6-phosphate 2-epimerase | 28.0/22.2 | Mid/Stat | 15.2/5.79 | NS |
| *M5005_spy0213* |  | N-acetylneuraminate-binding protein | 42.6/23.1 | Mid/Stat | 12.4/7.04 | NS |
| *M5005_spy0214* |  | N-acetylneuraminate transport system permease protein | 34.2/36.5 | Mid/Stat | 9.92/11.4 | NS |
| *M5005_spy0215* |  | N-acetylneuraminate transport system permease protein | 142/18.8 | Mid/Stat | 28.5/4.73 | 7.16/2.51 |
| *M5005_spy0216* |  | Hypothetical membrane spanning protein | 136./55.3 | Mid/Stat | 15.2/6.13 | 3.63/4.84 |
| *M5005_spy0218* |  | N-acetylmannosamine kinase | 24.6/24.6 | Mid/Stat | 5.95/6.51 | NS |
| *M5005_spy0361* |  | Phosphoglycerate transporter protein | 12.8/4.08 | Mid/Stat | 7.01/2.65 | NS |
| *M5005_spy0475* |  | PTS system, beta-glucoside-specific IIABC component | 17.9/21.1 | Mid/Stat | 5.90/8.41 | NS |
| *M5005_spy0476* | *bglA* | 6-phospho-beta-glucosidase | 21.0/20.7 | Mid/Stat | 5.24/9.58 | NS |
| *M5005_spy0519* | *agaD* | PTS system, N-acetylgalactosamine-specific IID component | 12.4/2.35 | Mid/Stat | 3.72 (M) | 4.22/2.42 |
| *M5005_spy0520* |  | PTS system, N-acetylgalactosamine-specific IIC component | 5.51/4.22 | Mid/Stat | NS | NS |
| *M5005_spy0521* | *agaV* | PTS system, N-acetylgalactosamine-specific IIB component | 3.76/2.90 | Mid/Stat | NS | NS |
| *M5005_spy0662* | *fruA* | PTS system, fructose-specific IIABC component | 3.85/0.89 | Mid/Stat | NS | 2.50 (M) |
| *M5005_spy0780* |  | PTS system, mannose/fructose family IIA component | 2.72 | Mid | 2.60 (M) | NS |
| *M5005_spy0781* | *ptsB* | PTS system, mannose/fructose family IIB component | 2.11 | Mid | 3.45 (M) | NS |
| *M5005_spy0782* | *ptsC* | PTS system, mannose/fructose family IIC component | 2.38 | Mid | 3.49 (M) | NS |
| *M5005_spy0783* | *ptsD* | PTS system, mannose/fructose family IID component | 2.77 | Mid | 3.43 (M) | NS |
| *M5005_spy0989* | *pfkA* | Non-allosteric 6-phosphofructokinase | 0.43 | Mid | NS | NS |
| *M5005_spy1055* | *malP* | Maltodextrin phosphorylase | 0.32 | Mid | NS | NS |
| *M5005_spy1058* | *malE* | Maltose/maltodextrin-binding protein malE | 0.25 | Stat | NS | NS |
| *M5005_spy1059* | *malF* | Maltose transport system permease protein malF | 0.21 | Stat | NS | 2.43 (S) |
| *M5005_spy1060* | *malG* | Maltose transport system permease protein malG | 0.41 | Stat | NS | 2.10 (S) |
| *M5005_spy1062* | *malA* | Maltodextrose utilization protein malA | 26.9/26.7 | Mid/Stat | 6.91/9.51 | 2.63 (S) |
| *M5005_spy1063* | *malD* | Maltodextrin transport system permease protein malD | 8.81/4.55 | Mid/Stat | 4.70/2.74 | NS |
| *M5005_spy1064* | *malC* | Maltose transport system permease protein malC | 6.08/3.90 | Mid/Stat | 5.86/2.34 | NS |
| *M5005_spy1065* | *amyA* | Alpha-amylase | 6.39/3.67 | Mid/Stat | 8.14/2.32 | NS |
| *M5005_spy1066* | *amyB* | Neopullulanase/cyclomaltodextrinase/maltogenic alpha-amylase | 10.0/5.91 | Mid/Stat | 5.38/3.13 | NS |
| *M5005_spy1067* | *malX* | Maltose/maltodextrin-binding protein malX | 9.14/4.09 | Mid/Stat | 6.80/2.14 | NS |
| *M5005_spy1079* |  | PTS system, cellobiose-specific IIC component | 17.2/11.4 | Mid/Stat | 8.87/3.11 | NS |
| *M5005_spy1081* |  | PTS system, cellobiose-specific IIA component | 8.97/5.64 | Mid/Stat | 7.61/1.84 | NS |
| *M5005_spy1082* |  | PTS system, cellobiose-specific IIB component | 13.1/6.71 | Mid/Stat | 8.88 (M) | NS |
| *M5005_spy1083* |  | Transcription antiterminator, BglG family/PTS system, mannitol (cryptic)-specific IIA component | 28.6/5.10 | Mid/Stat | 7.67/2.49 | 3.36/2.11 |
| *M5005_spy1085* | *bglA.2* | Beta-glucosidase | 5.20/4.05 | Mid/Stat | NS | NS |
| *M5005_spy1139* | *nagB* | Glucosamine-6-phosphate isomerase | 2.04 | Mid | NS | NS |
| *M5005_spy1235* | *N/A* | Phosphoglucomutase/phosphomannomutase | 2.77 | Mid | NS | NS |
| *M5005_spy1257* | *glcK* | Glucokinase/xylose represssor | 2.74 | Stat | NS | NS |
| *M5005_spy1304* | *lacZ* | Beta-galactosidase/Beta-glucosidase | 3.25/2.70 | Mid/Stat | NS | NS |
| *M5005_spy1308* |  | Sugar-binding protein | 3.82/3.01 | Mid/Stat | 2.06 (M) | NS |
| *M5005_spy1309* |  | Sugar transport system permease protein | 9.12/4.55 | Mid/Stat | 2.92 (S) | 5.85 (S) |
| *M5005_spy1310* |  | Sugar transport system permease protein | 3.15/2.39 | Mid/Stat | NS | NS |
| *M5005_spy1375* | *tkt* | Transketolase | 2.86/2.15 | Mid | 2.34 (M) | NS |
| *M5005_spy1376* |  | Transaldolase | 6.13/5.61 | Mid/Stat | 4.20/2.77 | NS |
| *M5005_spy1379* | *glpF* | Glycerol uptake facilitator protein | 21.2/9.56 | Mid/Stat | 11.2/2.84 | NS |
| *M5005_spy1395* | *lacD.1* | Tagatose-bisphosphate aldolase | 17.1/7.83 | Mid/Stat | 5.39 (M) | NS |
| *M5005_spy1396* | *nadE* | Tagatose-6-phosphate kinase | 9.02/6.69 | Mid/Stat | 6.23 (M) | NS |
| *M5005_spy1397* | *lacB.1* | Galactose-6-phosphate isomerase lacB subunit | 20.8/10.2 | Mid/Stat | 9.12 (M) | NS |
| *M5005_spy1398* | *lacA.1* | Galactose-6-phosphate isomerase lacA subunit | 56.8/22.5 | Mid/Stat | 7.21/2.80 | 2.27 (S) |
| *M5005_spy1399* |  | PTS system, galactose-specific IIC component | 16.5/3.35 | Mid/Stat | 5.96 (M) | NS |
| *M5005_spy1400* |  | PTS system, galactose-specific IIB component | 10.1/2.64 | Mid/Stat | 8.25 (M) | NS |
| *M5005_spy1401* |  | PTS system, galactose-specific IIA component | 20.2/2.18 | Mid/Stat | 7.13 (M) | NS |
| *M5005_spy1479* | *manL* | PTS system, mannose-specific IIAB component | 2.01 | Stat | NS | NS |
| *M5005_spy1480* | *manM* | PTS system, mannose-specific IIC component | 2.16 | Stat | NS | NS |
| *M5005_spy1481* | *manN* | PTS system, mannose-specific IID component | 2.36/2.13 | Mid/Stat | NS | NS |
| *M5005_spy1538* | *pmi* | Mannonse-6-phosphate isomerase | 24.0 | Stat | 9.02 (S) | 3.03 (S) |
| *M5005_spy1539* | *scrK* | Fructokinase | 35.6 (S) | Stat | 25.0 (S) | NS |
| *M5005_spy1542* | *scrA* | PTS system, sucrose-specific IIABC component | 30.3 | Stat | 24.2 (S) | 3.23 (S) |
| *M5005_spy1543* | *scrB* | Sucrose-6-phosphate hydrolase | 8.9 | Stat | 9.02 | NS |
| *M5005_spy1632* | *lacG* | 6-phospho-beta-glucosidase | 28.8 | Stat | -3.21/8.91 | 2.33 (S) |
| *M5005_spy1633* | *lacE* | PTS system, lactose-specific IIBC component | 16.8 | Stat | -2.52/7.52 | 2.71 (S) |
| *M5005_spy1634* | *lacF* | PTS system, lactose-specific IIA component | 13.8 | Stat | -2.71/6.39 | 2.47 (S) |
| *M5005_spy1635* | *lacD.2* | Tagatose-bisphosphate aldolase | 10.6 | Stat | -1.91/5.73 | NS |
| *M5005_spy1636* | *lacC.2* | Tagatose-6-phosphate kinase | 14.3 | Stat | -2.03/8.21 | 2.37 (S) |
| *M5005_spy1637* | *lacB.2* | Galactose-6-phosphate isomerase lacB subunit | 13.9 | Stat | -3.22/7.56 | 3.21 (S) |
| *M5005_spy1638* | *lacA.2* | Galactose-6-phosphate isomerase lacA subunit | 10.3 | Stat | -2.97/6.39 | 2.33 (S) |
| *M5005_spy1661* |  | Transaldolase | 8.94/6.55 | Mid/Stat | 3.35 (M) | NS |
| *M5005_spy1662* | *ulaA* | Ascorbate-specific PTS system enzyme IIC | 12.4/5.71 | Mid/Stat | 5.78/2.91 | 2.01 (M) |
| *M5005_spy1663* |  | PTS system, IIB component | 23.8/18.4 | Mid/Stat | 5.89/4.86 | NS |
| *M5005_spy1664* |  | PTS system, mannitol (cryptic)-specific IIA component | 5.09/3.09 | Mid/Stat | 3.10 (M) | NS |
| *M5005_spy1682* | *msmK* | Multiple sugar transport ATP-binding protein | 2.10 | Mid | NS | NS |
| *M5005_spy1692* |  | PTS system, glucose-specific IIABC component | 2.32 | Mid | NS | NS |
| *M5005_spy1693* |  | PTS system, glucose-specific IIABC component | 2.35 | Mid | NS | NS |
| *M5005_spy1744* |  | PTS system, cellobiose-specific IIC component | 6.22/5.46 | Mid/Stat | 2.07/3.31 | NS |
| *M5005_spy1745* |  | PTS system, cellobiose-specific IIB component | 15.9/6.45 | Mid/Stat | 4.31/3.56 | NS |
| *M5005_spy1746* |  | PTS system, cellobiose-specific IIA component | 10.7/4.69 | Mid/Stat | 3.03/2.00 | NS |
| *M5005_spy1783* | *dexS* | Trehalose-6-phosphate hydrolase | 2.93/1.27 | Mid/Stat | NS | 3.21/2.84 |
| *M5005_spy1784* |  | PTS system, trehalose-specific IIBC component | 3.42 | Mid | NS | 2.29/4.25 |
| **Cell motility** | | |  |  |  |  |
| *M5005_spy1007* |  | Phage protein | 2.55/2.55 | Mid/Stat | NS | NS |
| **Cellular processing** | | |  |  |  |  |
| *M5005_spy0835* |  | Class B acid phosphatase | 5.49/4.78 | Mid/Stat | 2.74 (M) | NS |
| *M5005_spy0836* |  | Acid phosphatase/phosphotransferase | 7.01/4.11 | Mid/Stat | 3.10 (M) | NS |
| **Cell wall/membrane biogenesis** | | |  |  |  |  |
| *M5005_spy0500* |  | N-acetylmuramoyl-L-alanine amidase | 2.33/3.30 | Mid/Stat | NS | NS |
| *M5005_spy0598* | *mscL* | Large-conductance mechanosensitive channel | 2.44 | Mid | NS | NS |
| *M5005_spy1843* |  | Transglycosylase SLT domain-containing protein | 5.28/6.88 | Mid/Stat | 5.54 (S) | NS |
| *M5005_spy1851* | *hasA* | Hyaluronan synthase | 25.2/73.0 | Mid/Stat | NS | 41.3/49.6 |
| *M5005_spy1852* | *hasB* | UDP-glucose 6-dehydrogenase | 17.7/47.9 | Mid/Stat | NS | 32.8/36.6 |
| *M5005_spy1853* | *hasC* | UTP-glucose-1-phosphate uridylyltransferase | 16.3/30.7 | Mid/Stat | NS | 29.0/24.5 |
| **Coenzyme and cofactor metabolism** | | |  |  |  |  |
| M5005_spy0898 |  | 2-(5’’-triphosphoribosyl)-3’-dephosphocoenzyme-A synthase | 0.47 | Stat | NS | NS |
| *M5005_spy0908* | *citX* | Apo-citrate lyase phosphoribosyl-dephospho-CoA transferase | 5.96/3.40 | Mid/Stat | 5.90 (M) | NS |
| *M5005_spy0945* | *coaA* | Pantothenate kinase | 2.36/8.01 | Mid/Stat | NS | NS |
| *M5005_spy1357* | *nadE* | NAD synthatase | -5.41 | Mid | NS | NS |
| **Defense mechanisms/virulence** | | |  |  |  |  |
| *M5005_spy0041* |  | Na+ driven multidrug efflux pump | 2.12 | Mid | NS | NS |
| *M5005_spy0042* |  | Na+ driven multidrug efflux pump | 2.09 | Mid | NS | NS |
| *M5005_spy0139* | *nga* | NAD glycohydrolase | 31.2/28.6 | Mid/Stat | 3.56 (S) | 12.1/21.3 |
| *M5005_spy0141* | *slo* | Streptolysin O | 25.3/32.5 | Mid/Stat | 2.73 (S) | 12.3/25.2 |
| *M5005_spy0341* | *spyCEP* | IL-8-degrading proteinase | 25.5/34.2 | Mid/Stat | 8.33/7.43 | 17.4/10.1 |
| *M5005_spy0351* | *spyA* | ADP-ribosyltransferase, C3 family | 5.2/10.3 | Mid/Stat | NS | 3.78/14.7 |
| *M5005_spy0356* | *speJ* | Exotoxin type J precursor | 2.2/5.1 | Mid/Stat | NS | 2.77/3.14 |
| *M5005_spy0561* | *epf* | Putative extracellular matrix binding protein | 17.0/11.1 | Mid/Stat | NS | 14.7/15.3 |
| *M5005_spy0562* | *sagA* | Streptolysin S precursor | 8.35/8.36 | Mid/Stat | 2.68 (M) | 2.31/5.34 |
| *M5005_spy0563* | *sagB* | Streptolysin S biosynthesis protein sagB | 10.5/19.2 | Mid/Stat | 3.65/5.28 | 3.21/15.5 |
| *M5005_spy0564* | *sagC* | Streptolysin S biosynthesis protein sagC | 8.84/15.3 | Mid/Stat | 3.12/4.12 | 2.81/8.71 |
| *M5005_spy0565* | *sagD* | Streptolysin S biosynthesis protein sagD | 9.79/12.5 | Mid/Stat | 3.82/4.60 | 4.02/10.0 |
| *M5005_spy0566* | *sagE* | Streptolysin S putative self-immunity protein sagE | 10.4/19.3 | Mid/Stat | 3.73/5.84 | 3.01/14.9 |
| *M5005_spy0567* | *sagF* | Streptolysin S biosynthesis protein sagF | 6.15/9.57 | Mid/Stat | 3.14/4.65 | 2.56/11.2 |
| *M5005_spy0568* | *sagG* | Streptolysin S export ATP-binding protein sagG | 5.32/8.30 | Mid/Stat | NS | 2.07/6.57 |
| *M5005_spy0569* | *sagH* | Streptolysin S export transmembrane protein sagH | 6.50/10.1 | Mid/Stat | 3.03/3.93 | 2.38/8.12 |
| *M5005_spy0570* | *sagI* | Streptolysin S export transmembrane protein sagI | 5.22/6.94 | Mid/Stat | 2.23/3.01 | 2.15/5.51 |
| *M5005_spy0571* |  | Endonuclease/exonuclease/phosphatase family protein | 3.89/3.87 | Mid/Stat | NS | NS |
| *M5005_spy0667* |  | Exotoxin type C precursor | 40.2/83.4 | Mid/Stat | NS | 41.6/75.9 |
| *M5005_spy0668* | *mac* | IgG-degrading protease of GAS | 30.8/46.4 | Mid/Stat | NS | 38.8/50.2 |
| *M5005_spy0803* | *srtI* | Protein involved in lantibiotic (srt) production | 3.16 | Mid | NS | 2.71/2.11 |
| *M5005_spy0996* | *speA2* | Exotoxin type A precursor, A2 allele | 4.14/8.65 | Mid/Stat | NS | 7.27/19.5 |
| *M5005_spy1012* |  | Antigen A | 2.52/2.10 | Mid/Stat | NS | NS |
| *M5005_spy1013* |  | Antigen B | 2.57/3.13 | Mid/Stat | NS | NS |
| *M5005_spy1106* | *grab* | Protein G related α-2M binding protein | 2.32 | Mid | NS | 3.57 (M) |
| *M5005_spy1415* | *sdaD2* | Streptodornase | 2.2/10.1 | Mid/Stat | 2.31 (S) | 2.69/7.86 |
| *M5005_spy1540* | *endoS* | Endo-beta-N-acetylglucosaminidase F2 precursor | 26.6 (S) | Stat | 21.3 (S) | 2.61 (S) |
| *M5005_spy1684* | *ska* | Streptokinase | 2.34/17.6 | Mid/Stat | NS | 3.24/13.3 |
| *M5005_spy1687* | *sclA* | Collagen-like surface protein A | 33.9/41.1 | Mid/Stat | NS | 37.4/17.5 |
| *M5005_spy1688* |  | Immunoglobulin receptor precursor | 98.2/35.3 | Mid/Stat | NS | 2.29 (M) |
| *M5005_spy1689* |  | Collagen-like surface protein | 19.2/453 | Mid/Stat | NS | 21.4/196 |
| *M5005_spy1691* |  | Endonuclease/exonuclease/phosphatase family protein | 7.65 | Mid | 3.15/2.01 | 2.97/4.24 |
| *M5005_spy1714* | *fba* | Fibronectin binding protein | 5.01 | Stat | 3.23 (S) | 4.12 (S) |
| *M5005_spy1715* | *scpA* | C5a peptidase precursor protein | 5.23 | Stat | 2.23 (S) | 5.13 (S) |
| *M5005_spy1718* | *sic* | Streptococcal inhibitor of complement | 4.23 | Stat | NS | 2.39 (S) |
| *M5005_spy1735* | *speB* | Cysteine protease | -5.23 | Mid | -5.21 (M) | 2.23 (M) |
| *M5005_spy1738* | *sda* | Streptodornase | 3.02 | Mid | NS | 2.24 (M) |
| **Energy production and conversion** | | |  |  |  |  |
| *M5005_spy0039* | *adh2* | Alcohol/acetaldehyde-CoA dehydrogenase | 11.0 | Mid | NS | 2.60/2.23 |
| *M5005_spy0040* | *adhA* | Alcohol dehydrogenase | 12.1/4.14 | Mid/Stat | 5.91/2.52 | NS |
| *M5005_spy0126* | *ntpI* | V-type sodium ATP synthase subunit I | 4.32/29.2 | Mid/Stat | 12.3/0.18 | NS |
| *M5005_spy0127* | *ntpK* | V-type sodium ATP synthase subunit K | 3.58/20.9 | Mid/Stat | 11.3/3.11 | NS |
| *M5005_spy0128* | *ntpE* | V-type sodium ATP synthase subunit E | 4.21/17.0 | Mid/Stat | 11.0/3.61 | NS |
| *M5005_spy0129* | *ntpC* | V-type ATP synthase subunit C | 7.92/26.5 | Mid/Stat | 18.9/4.09 | NS |
| *M5005_spy0130* | *ntpF* | V-type ATP synthase subunit F | 3.92/13.6 | Mid/Stat | 11.8/3.11 | NS |
| *M5005_spy0131* | *ntpA* | V-type sodium ATP synthase subunit A | 3.46/12.1 | Mid/Stat | 9.47/2.85 | NS |
| *M5005_spy0132* | *ntpB* | V-type sodium ATP synthase subunit B | 4.87/19.4 | Mid/Stat | 12.6/3.61 | NS |
| *M5005_spy0133* | *ntpD* | V-type sodium ATP synthase subunit D | 4.83/18.5 | Mid/Stat | 14.4/3.32 | NS |
| *M5005_spy0340* | *lctO* | L-lactate oxidase | 39.4/6.16 | Mid/Stat | 13.8/2.19 | NS |
| *M5005_spy0790* | *gabD* | Succinate-semialdehyde dehydrogenase [NADP+] | 2.96/3.88 | Mid/Stat | NS | NS |
| *M5005_spy0900* |  | Mg2+/citrate complex secondary transporter | 5.76/7.97 | Mid/Stat | NS | NS |
| *M5005_spy0903* | *oadB* | Oxaloacetate decarboxylase beta chain | 2.06 | Mid | NS | 2.70 (S) |
| *M5005_spy0905* | *citD* | Citrate lyase subunit gamma/acyl carrier protein | 2.44 | Mid | NS | NS |
| *M5005_spy0906* | *citE* | Citrate lyase beta chain/citryl-CoA lyase subunit | 2.29 | Mid | NS | 2.13 (S) |
| *M5005_spy0907* | *citF* | Citrate lyase alpha chain/citrate CoA-transferase | 2.52 | Mid | NS | 2.16 (S) |
| *M5005_spy0909* | *oadA* | Oxaloacetate decarboxylase alpha chain | 2.26/0.40 | Mid/Stat | NS | NS |
| *M5005_spy1380* | *glpO* | Alpha-glycerophosphate oxidase | 11.2/9.97 | Mid/Stat | 5.07/3.23 | NS |
| *M5005_spy1381* | *glpK* | Glycerol kinase | 8.27/7.06 | Mid/Stat | 2.63 (M) | NS |
| **Inorganic ion transport and metabolism** | | |  |  |  |  |
| *M5005_spy0543* | *adcA* | High-affinity zinc uptake system protein znuA precursor | 2.40 | Mid | NS | 2.28 (M) |
| *M5005_spy0786* |  | Iron(III)-binding protein | 2.33 | Stat | NS | NS |
| *M5005_spy0985* |  | PhnA protein | -3.52 | Mid | NS | NS |
| *M5005_spy1161* |  | Formate transporter | 3.16/2.71 | Mid/Stat | NS | NS |
| *M5005_spy1167* |  | Lead, cadmium, zinc and mercury transporting ATPase | 2.30 | Mid | NS | NS |
| *M5005_spy1403* |  | Copper chaperone | 3.24 | Mid | NS | 3.43/3.48 |
| *M5005_spy1711* | *lmb* | Laminin binding protein | 2.92 | Mid | NS | 2.00 (M) |
| **Intracellular trafficking and secretion** | | |  |  |  |  |
| *M5005_spy0664* | *mur1.2* | Autolysin | 2.37/2.38 | Mid/Stat | NS | NS |
| *M5005_spy1751* | *secE* | Protein translocase subunit secE | 2.19 | Mid | NS | NS |
| **Lipid transport and metabolism** | | |  |  |  |  |
| *M5005_spy0116* | *atoE* | Short-chain fatty acids transporter | 11.9 | Mid | NS | NS |
| *M5005_spy0119* |  | Acetyl-CoA acetyltransferase | 3.95/3.42 | Mid/Stat | NS | NS |
| *M5005_spy0120* | *atoD.2* | Acetate CoA-transferase alpha subunit | 2.87/6.96 | Mid/Stat | NS | NS |
| *M5005_spy0121* |  | Acetyl-CoA:acetoacetyl-CoA transferase beta subunit | 3.22/5.72 | Mid/Stat | NS | NS |
| *M5005_spy0359* | *fabG* | 3-ketoacyl-acyl carrier protein reductase | -2.16 | Stat | NS | -2.56 (S) |
| *M5005_spy0534* | *bsaA* | Acetoin (diacetyl) reductase | 3.31 | Mid | NS | NS |
| *M5005_spy0535* |  | Acetoin dehydrogenase | 5.12/1.68 | Mid/Stat | 2.30 (M) | NS |
| *M5005_spy0902* |  | Acetyl-CoA carboxylase biotin carboxyl carrier protein subunit | 2.48/0.08 | Mid/Stat | NS | 2.40 (S) |
| **Nucleotide transport and metabolism** | | |  |  |  |  |
| *M5005_spy0080* |  | Bis(5’-nucleosyl)-tetraphosphatase | 3.22 | Stat | 5.82 (S) | -4.90 (S) |
| *M5005_spy0639* | *pyrR* | Pyramidine regulatory protein | -5.82 | Stat | NS | -6.88 (S) |
| *M5005_spy0640* | *pyrP* | Uracil permease | -6.23 | Stat | NS | -8.72 (S) |
| *M5005_spy0641* | *pyrB* | Aspartate carbamoyl transferase | -6.71 | Stat | NS | -5.51 (S) |
| *M5005_spy0642* | *carA* | Carbamoyl phosphate synthase | -5.85 | Stat | NS | -5.69 (S) |
| *M5005_spy0643* | *carB* | Carbamoyl phosphate synthase | -4.82 | Stat | NS | -5.71 (S) |
| *M5005_spy0678* |  | 5’-nucleotidase | 4.89/8.65 | Mid/Stat | 2.25/4.59 | NS |
| *M5005_spy0775* |  | Nucleoside diphosphate kinase | 2.70 | Mid | NS | 2.02 (M) |
| *M5005_spy0857* | *guaC* | GMP reductase | 2.16 | Mid | NS | NS |
| *M5005_spy0858* | *xpt* | Xanthine phosphoribosyl transferase | -10.3 | Mid | NS | NS |
| *M5005_spy0859* |  | Xanthine permease | -12.2 | Mid | NS | NS |
| *M5005_spy0939* |  | Nucleoside transport system permease protein | 2.16/2.98 | Mid/Stat | NS | NS |
| *M5005_spy1477* |  | Guanine/hypoxanthine permease | -8.7 | Mid | NS | NS |
| *M5005_spy1585* | *deoC* | Deoxyribose-phosphate aldolase | 2.00 | Mid | NS | NS |
| *M5005_spy1587* | *udp* | Uridine phosphorylase | 2.75 | Mid | NS | NS |
| **Phage** | |  |  |  |  |  |
| *M5005_spy0459* |  | Portal protein | 5.99 | Mid | 5.01 (M) | 2.26 (M) |
| *M5005_spy0995* |  | Phage protein | 8.54/11.9 | Mid/Stat | 2.73/4.82 | 2.51 (M) |
| *M5005_spy0997* |  | Phage protein | 3.78/2.20 | Mid/Stat | NS | NS |
| *M5005_spy0999* |  | Phage protein | 8.82/2.20 | Mid/Stat | 2.09 (M) | NS |
| *M5005_spy1000* |  | Phage protein | 2.92/4.64 | Mid/Stat | NS | NS |
| *M5005_spy1004* |  | Phage protein | 3.30/5.00 | Mid/Stat | NS | NS |
| *M5005_spy1005* |  | Phage protein | 2.12/2.08 | Mid/Stat | NS | NS |
| *M5005_spy1006* |  | Phage structural protein | 10.3/3.50 | Mid/Stat | NS | NS |
| *M5005_spy1009* |  | Phage protein | 4.50/2.04 | Mid/Stat | NS | NS |
| *M5005_spy1010* |  | Phage protein | 9.10/6.17 | Mid/Stat | NS | NS |
| *M5005_spy1011* |  | Phage protein | 4.82/6.78 | Mid/Stat | NS | NS |
| *M5005_spy1017* |  | Phage protein | 2.56/3.21 | Mid/Stat | NS | NS |
| *M5005_spy1018* |  | Phage protein | 2.32 | Mid | NS | NS |
| *M5005_spy1019* |  | Phage scaffold protein | 8.21/11.6 | Mid/Stat | NS | NS |
| *M5005_spy1020* |  | Phage protein | 2.84 | Mid | NS | NS |
| *M5005_spy1021* |  | Phage protein | 13.2/9.11 | Mid/Stat | 3.21 (S) | 4.53 (M) |
| *M5005_spy1022* |  | Portal protein | 2.93/2.42 | Mid/Stat | NS | 2.53 (M) |
| *M5005_spy1029* |  | Phage protein | 2.43/2.99 | Mid/Stat | NS | NS |
| *M5005_spy1038* |  | Phage protein | 2.12 | Mid | NS | NS |
| *M5005_spy1047* |  | Phage protein | 2.12/2.17 | Mid/Stat | NS | NS |
| *M5005_spy1049* |  | Phage protein | 10.8 | Mid | NS | NS |
| *M5005_spy1172* |  | Holin | 2.90 | Mid | 2.35 (M) | NS |
| *M5005_spy1173* |  | Phage protein | 5.08 | Mid | 2.88 (M) | 5.35 (M) |
| *M5005_spy1175* |  | Phage protein | 12.1 | Mid | NS | 3.40 (M) |
| *M5005_spy1176* |  | Phage infection protein | 2.08/5.87 | Mid/Stat | 4.49/3.29 | NS |
| *M5005_spy1201* |  | Phage protein | 2.75 | Mid | 3.81 (M) | NS |
| *M5005_spy1203* |  | Phage protein | 5.12/5.33 | Mid/Stat | NS | NS |
| *M5005_spy1429* |  | Phage protein | -2.19 | Mid | NS | -2.28/-4.08 |
| **Post-translational modification, protein turnover, chaperones** | | |  |  |  |  |
| *M5005_spy1080* |  | Hypothetical protein | 8.76/7.30 | Mid/Stat | 2.39/2.45 | NS |
| *M5005_spy1282* | *msrA* | Bifunctional methionine sulfoxide reductase A/B peptide | 3.44 | Mid | 3.44 (M) | NS |
| **Replication, recombination and repair** | | |  |  |  |  |
| *M5005_spy0113* |  | Transposase | 18.0/11.7 | Mid/Stat | NS | 97.5/11.5 |
| *M5005_spy0254* |  | Transposase | 6.48/4.07 | Mid/Stat | 4.70/2.32 | 4.50 (M) |
| *M5005_spy0800* |  | DNA-cytosine methyltransferase | 4.59/4.85 | Mid/Stat | NS | NS |
| *M5005_spy0840* | *radC* | DNA repair protein radC | 2.97 | Mid | NS | NS |
| *M5005_spy1043* |  | Phage protein | 2.76 | Mid | NS | NS |
| *M5005_spy1285* |  | Hypothetical protein | 4.40/6.31 | Mid/Stat | NS | 2.04/5.73 |
| *M5005_spy1286* |  | DNA polymerase | 3.43/4.84 | Mid/Stat | NS | NS |
| *M5005_spy1287* |  | Hypothetical protein | 4.22/6.93 | Mid/Stat | NS | 2.08/7.95 |
| *M5005_spy1643* |  | DNA integration/recombination/invertion protein | 2.48 | Mid | 2.42 (M) | NS |
| **Signal transduction mechanisms** | | |  |  |  |  |
| *M5005_spy1276* |  | Transcription regulator, Crp family | 2.80/2.35 | Mid/Stat | NS | NS |
| *M5005_spy1305* | *lytR* | Two-component response regulator, yesN | 3.11/2.12 | Mid/Stat | NS | NS |
| *M5005_spy1306* | *lytS* | Two-component sensor kinase, yesM | 3.19/2.07 | Mid/Stat | NS | NS |
| *M5005_spy1574* |  | Universal stress protein | -3.32 | Stat | NS | NS |
| **Stress** | |  |  |  |  |  |
| *M5005_spy1378* |  | NADH peroxidase | 13.2/8.21 | Mid/Stat | 6.47/3.08 | NS |
| **Transcription** | | |  |  |  |  |
| *M5005_spy0117* |  | Transcriptional regulators, LysR family | 14.1/16.5 | Mid/Stat | 4.91/4.27 | NS |
| *M5005_spy0118* |  | Transcriptional regulator, LysR family | 15.9/16.4 | Mid/Stat | 4.73/4.64 | NS |
| *M5005_spy0124* | *sloR* | Transcriptional regulator | -4.25 | Mid | NS | NS |
| *M5005_spy0474* | *licT* | Transcription antiterminator, BglG family | 17.0/30.6 | Mid/Stat | 6.41/12.2 | NS |
| *M5005_spy1045* |  | Transcriptional regulator | 2.02 | Mid | NS | NS |
| *M5005_spy1061* |  | Transcriptional regulator, LacI family | 0.37/0.27 | Mid/Stat | NS | NS |
| *M5005_spy1277* | *ahrC.2* | Arginine repressor, ArgR family | 3.01/3.13 | Mid/Stat | NS | NS |
| *M5005_spy1315* |  | Transcriptional regulator, GntR family | 2.04 | Mid | NS | NS |
| *M5005_spy1377* |  | Trans-acting positive regulator | 10.5/6.52 | Mid/Stat | 4.78/2.49 | NS |
| *M5005_spy1392* |  | Transcriptional regulator, TetR family | 2.16/2.21 | Mid/Stat | NS | NS |
| *M5005_spy1578* |  | Transcriptional regulator, Cro/CI family | 2.86 | Mid | NS | NS |
| *M5005_spy1668* |  | Putative transcriptional regulator | 3.60/4.00 | Mid/Stat | NS | NS |
| *M5005_spy1760* |  | Transcriptional regulator, MutR family | 39.6/4.59 | Mid/Stat | 8.39 (M) | 4.63 (M) |
| *M5005_spy1779* |  | Transcriptional regulator, LuxR family | 23.1/16.8 | Mid/Stat | 11.8/7.66 | NS |
| *M5005_spy1825* |  | Transcriptional regulator, PhdR family | -8.76 | Stat | NS | NS |
| **Translation** | |  |  |  |  |  |
| *M5005_spy0123* |  | Translation initiation inhibitor | -4.25 | Mid | NS | NS |
| *M5005_spy0798* |  | IFN-response binding factor 1 | 4.30 | Mid | 3.38 (M) | 5.92 (M) |
| **Unknown** | |  |  |  |  |  |
| *M5005_spy0015* |  | Hypothetical protein | 14.2/2.18 | Mid/Stat | NS | 5.28 (M) |
| *M5005_spy0098* |  | Hypothetical protein | 2.14 | Mid | NS | NS |
| *M5005_spy0115* |  | Hypothetical protein | 48.3/265 | Mid/Stat | NS | 63.0/300 |
| *M5005_spy0125* |  | Hypothetical protein | 4.47/30.7 | Mid/Stat | 12.5/4.29 | NS |
| *M5005_spy0142* |  | Hypothetical protein | 3.22/9.28 | Mid/Stat | NS | 313/165 |
| *M5005_spy0143* |  | Hypothetical protein | 122/112 | Mid/Stat | NS | 171.94.5 |
| *M5005_spy0144* |  | Hypothetical protein | 382/351 | Mid/Stat | NS | NS |
| *M5005_spy0177* |  | BioY protein | 2.19/2.79 | Mid/Stat | NS | NS |
| *M5005_spy0281* |  | Hypothetical cytosolic protein | 2.33/4.36 | Mid/Stat | NS | 2.78/3.77 |
| *M5005_spy0352* |  | Hypothetical membrane associated protein | 31.7/69.0 | Mid/Stat | NS | 30.4/45.0 |
| *M5005_spy0353* |  | Hypothetical membrane spanning protein | 4.24/16.8 | Mid/Stat | NS | 2.68/8.17 |
| *M5005_spy0354* |  | Hypothetical protein | 6.30/10.6 | Mid/Stat | NS | 11.2/7.54 |
| *M5005_spy0355* |  | Hypothetical protein | 16.8/128 | Mid/Stat | NS | 38.4/81.8 |
| *M5005_spy0357* |  | Hypothetical protein | 3.14/5.05 | Mid/Stat | NS | 5.12/2.69 |
| *M5005_spy0360* |  | NAD-dependent oxidoreductase | -2.28 | Stat | NS | -2.56 (S) |
| *M5005_spy0404* |  | Hypothetical protein | 2.35 | Mid | NS | NS |
| *M5005_spy0518* |  | Oligohyaluronate lyase | 5.71/11.3 | Mid/Stat | 2.21/2.12 | 2.41/6.17 |
| *M5005_spy0666* |  | Hypothetical protein | 47.3/33.5 | Mid/Stat | NS | 49.3/36.3 |
| *M5005_spy0742* |  | Hypothetical protein | 2.64/2.19 | Mid/Stat | NS | NS |
| *M5005_spy0773* |  | Hypothetical protein | 14.8/4.99 | Mid/Stat | 5.63 (M) | 7.48/3.49 |
| *M5005_spy0812* |  | Hypothetical protein | 5.09 | Mid | 4.29 (M) | 3.35 (M) |
| *M5005_spy0852* |  | Short chain dehydrogenase | 2.71 | Mid | NS | NS |
| *M5005_spy0853* |  | Short chain dehydrogenase | 2.75 | Mid | NS | NS |
| *M5005_spy0880* |  | Hypothetical protein | -3.56 | Stat | NS | NS |
| *M5005_spy0979* |  | Hypothetical protein | 2.36 | Mid | NS | NS |
| *M5005_spy0981* | *cfa* | cAMP factor | 4.33 | Mid | NS | NS |
| *M5005_spy1001* |  | Phage-associated cell wall hydrolase | 2.36/2.60 | Mid/Stat | NS | NS |
| *M5005_spy1008* |  | Hypothetical protein | 4.21/3.27 | Mid/Stat | NS | NS |
| *M5005_spy1023* |  | Terminase large subunit | 4.38 | Mid | NS | NS |
| *M5005_spy1078* |  | Hypothetical protein | 6.58/3.66 | Mid/Stat | 3.66/2.17 | NS |
| *M5005_spy1084* |  | Outer surface protein | 53.9/41.8 | Mid/Stat | 4.35/3.31 | NS |
| *M5005_spy1093* |  | Hypothetical protein | 29.1/6.00 | Mid/Stat | 7.49 (M) | NS |
| *M5005_spy1142* |  | Hypothetical protein | 18.4/5.21 | Mid/Stat | NS | 16.1/3.97 |
| *M5005_spy1143* |  | Hypothetical protein | 6.57/4.15 | Mid/Stat | NS | 6.56/2.40 |
| *M5005_spy1144* |  | Hypothetical protein | 3.53/3.09 | Mid/Stat | NS | 3.97/2.44 |
| *M5005_spy1289* |  | Hypothetical protein | 3.64/6.20 | Mid/Stat | NS | NS |
| *M5005_spy1290* |  | Hypothetical protein | 4.96/9.93 | Mid/Stat | NS | 2.11/15.7 |
| *M5005_spy1307* |  | Hypothetical protein | 4.29/2.61 | Mid/Stat | NS | NS |
| *M5005_spy1541* |  | Hypothetical protein | 33.2 | Stat | 27.3 (S) | 3.05 (S) |
| *M5005_spy1556* |  | Hypothetical protein | 4.41/7.24 | Mid/Stat | NS | 5.12/8.80 |
| *M5005_spy1667* |  | Hypothetical protein | 4.07 | Mid | 3.50 (M) | 3.22 (M) |
| *M5005_spy1703* |  | Hypothetical cytosolic protein | 8.10 | Mid | 4.41 (M) | 6.81 (M) |
| *M5005_spy1731* |  | Hypothetical cytosolic protein | 4.16/38.8 | Mid/Stat | NS | 3.72/4.18 |

| **M5005 ORF** | **Gene name** | **Putative function of encoded protein** | **Fold-change^1^** | **Time-point** | **Regulated in strain 2221∆*ccpA***^2^ | **Regulated in strain 2221∆*covR*** |
| --- | --- | --- | --- | --- | --- | --- |
| **Amino acid transport and metabolism** | | |  |  |  |  |
| *M5005_spy0146* | *metB* | Cystathionine beta-lyase | 2.90 | Mid | NS | 2.45 (M) |
| *M5005_spy0157* | *opuAA* | Glycine betaine transport ATP binding protein | -4.91 | Mid | NS | NS |
| *M5005_spy0158* | *opuABC* | Glycine betaine binding protein | -5.21 | Mid | NS | NS |
| *M5005_spy0217* | *nanH* | N-acetylneuraminate lyase | 19.8/20.7 | Mid/Stat | 5.59/5.46 | 2.59/3.05 |
| *M5005_spy0834* |  | Zn-dependent alcohol dehydrogenases and related dehydrogenases | 15.0/3.22 | Mid/Stat | 2.44 (M) | NS |
| *M5005_spy0855* | *proV* | Glycine betaine transport ATP-binding protein | 2.32 | Mid | NS | NS |
| *M5005_spy0856* |  | Glycine betaine transport system permease protein | 2.08 | Mid | NS | NS |
| *M5005_spy0982* |  | Histidine binding protein | -2.91 | Mid | NS | NS |
| *M5005_spy0983* |  | Histidine transport ATP binding protein | -4.51 | Mid | NS | NS |
| *M5005_spy0984* |  | Histidine transport system permease | -3.82 | Mid | NS | NS |
| *M5005_spy1094* |  | Transporter, MFS superfamily | 2.53 | Mid | NS | NS |
| *M5005_spy1181* |  | Major tail protein | 4.30/3.31 | Mid/Stat | 3.14/2.08 | NS |
| *M5005_spy1237* | *artP* | Arginine transport ATP-binding protein | 5.40 | Stat | 8.80 (S) | NS |
| *M5005_spy1238* | *artQ* | Arginine transport system permease protein | 4.10 | Stat | 12.2 (S) | NS |
| *M5005_spy1269* | *asnA* | Aspartate—ammonia ligase/asparagine synthetase | 2.20 | Stat | 3.17 (S) | 2.35 (M) |
| *M5005_spy1270* | *arcC* | Carbamate kinase | 611/4.60 | Mid/Stat | 372/2.95 | 4.31/2.35 |
| *M5005_spy1271* |  | Xaa-His dipeptidase | 111/5.36 | Mid/Stat | 54.2/2.87 | 3.93/2.27 |
| *M5005_spy1272* |  | Arginine/ornithine antiporter | 53.9/4.25 | Mid/Stat | 26.4/2.58 | NS |
| *M5005_spy1273* | *arcB* | Ornithine carbamoyltransferase | 31.7/2.43 | Mid/Stat | 19.0 (M) | 3.52 (M) |
| *M5005_spy1274* |  | Acetyltransferase | 25.6 | Mid | 20.3 (M) | 2.91 (M) |
| *M5005_spy1275* | *arcA* | Arginine deiminase | 28.8 | Mid | 22.1 (M) | 2.27 (M) |
| *M5005_spy1704* | *dppA* | Dipeptide transport | -5.13 | Mid | NS | -3.13 (M) |
| *M5005_spy1705* | *dppB* | Dipeptide transport | -5.83 | Mid | NS | -2.97 (M) |
| *M5005_spy1706* | *dppC* | Dipeptide transport | -5.29 | Mid | NS | -2.80 (M) |
| *M5005_spy1707* | *dppD* | Dipeptide transport | -4.91 | Mid | NS | -2.71 (M) |
| *M5005_spy1708* | *dppE* | Dipeptide transport | -5.26 | Mid | NS | -2.56 (M) |
| *M5005_spy1710* |  | Streptococcal histidine triad protein | 2.28 | Mid | NS | NS |
| *M5005_spy1758* |  | Dipeptidase B | 12.7/2.31 | Mid/Stat | 3.08 (M) | NS |
| *M5005_spy1770* | *hutI* | Imidazolonepropionase | 60.4/13.1 | Mid/Stat | 17.5/3.84 | NS |
| *M5005_spy1771* | *hutU* | Urocanate hydratase | 4.62/4.07 | Mid/Stat | NS | NS |
| *M5005_spy1772* |  | Glutamate formiminotransferase | 9.25/10.9 | Mid/Stat | NS | NS |
| *M5005_spy1773* |  | Formiminotetrahydrofolate cyclodeaminase | 2.26/3.22 | Mid/Stat | NS | 2.40 (S) |
| *M5005_spy1774* |  | Formate—tetrahydrofolate ligase | 2.74/2.29 | Mid/Stat | 2.70 (S) | 2.60 (S) |
| *M5005-_spy1775* |  | Hypothetical cytosolic protein | 16.4/6.64 | Mid/Stat | 6.37 (S) | 2.90 (S) |
| *M5005_spy1776* |  | Amino acid permease | 2.50/3.62 | Mid/Stat | NS | NS |
| *M5005_spy1777* | *hutH* | Histidine ammonia-lyase | 4.60/4.71 | Mid/Stat | 2.30 (S) | 2.10 (S) |
| *M5005_spy1778* | *hutG* | Formiminoglutamase | 2.29 | Stat | 3.10 (S) | 2.30 (S) |
| *M5005_spy1841* | *sdhB* | L-serine dehydratase | -7.65 | Mid | NS | NS |
| **Carbohydrate transport and metabolism** | | |  |  |  |  |
| *M5005_spy0151* |  | 3-keto-L-gulonate-6-phosphate decarboxylase | 3.41 | Mid | 2.09 (M) | 3.37 (M) |
| *M5005_spy0152* |  | L-xylulose 5-phosphate 3-epimerase | 10.4/4.36 | Mid/Stat | NS | NS |
| *M5005_spy0153* | *araD* | L-ribulose-5-phosphate 4-epimerase | 2.83 | Mid | NS | NS |
| *M5005_spy0212* |  | N-acetylmannosamine-6-phosphate 2-epimerase | 28.0/22.2 | Mid/Stat | 15.2/5.79 | NS |
| *M5005_spy0213* |  | N-acetylneuraminate-binding protein | 42.6/23.1 | Mid/Stat | 12.4/7.04 | NS |
| *M5005_spy0214* |  | N-acetylneuraminate transport system permease protein | 34.2/36.5 | Mid/Stat | 9.92/11.4 | NS |
| *M5005_spy0215* |  | N-acetylneuraminate transport system permease protein | 142/18.8 | Mid/Stat | 28.5/4.73 | 7.16/2.51 |
| *M5005_spy0216* |  | Hypothetical membrane spanning protein | 136./55.3 | Mid/Stat | 15.2/6.13 | 3.63/4.84 |
| *M5005_spy0218* |  | N-acetylmannosamine kinase | 24.6/24.6 | Mid/Stat | 5.95/6.51 | NS |
| *M5005_spy0361* |  | Phosphoglycerate transporter protein | 12.8/4.08 | Mid/Stat | 7.01/2.65 | NS |
| *M5005_spy0475* |  | PTS system, beta-glucoside-specific IIABC component | 17.9/21.1 | Mid/Stat | 5.90/8.41 | NS |
| *M5005_spy0476* | *bglA* | 6-phospho-beta-glucosidase | 21.0/20.7 | Mid/Stat | 5.24/9.58 | NS |
| *M5005_spy0519* | *agaD* | PTS system, N-acetylgalactosamine-specific IID component | 12.4/2.35 | Mid/Stat | 3.72 (M) | 4.22/2.42 |
| *M5005_spy0520* |  | PTS system, N-acetylgalactosamine-specific IIC component | 5.51/4.22 | Mid/Stat | NS | NS |
| *M5005_spy0521* | *agaV* | PTS system, N-acetylgalactosamine-specific IIB component | 3.76/2.90 | Mid/Stat | NS | NS |
| *M5005_spy0662* | *fruA* | PTS system, fructose-specific IIABC component | 3.85/0.89 | Mid/Stat | NS | 2.50 (M) |
| *M5005_spy0780* |  | PTS system, mannose/fructose family IIA component | 2.72 | Mid | 2.60 (M) | NS |
| *M5005_spy0781* | *ptsB* | PTS system, mannose/fructose family IIB component | 2.11 | Mid | 3.45 (M) | NS |
| *M5005_spy0782* | *ptsC* | PTS system, mannose/fructose family IIC component | 2.38 | Mid | 3.49 (M) | NS |
| *M5005_spy0783* | *ptsD* | PTS system, mannose/fructose family IID component | 2.77 | Mid | 3.43 (M) | NS |
| *M5005_spy0989* | *pfkA* | Non-allosteric 6-phosphofructokinase | 0.43 | Mid | NS | NS |
| *M5005_spy1055* | *malP* | Maltodextrin phosphorylase | 0.32 | Mid | NS | NS |
| *M5005_spy1058* | *malE* | Maltose/maltodextrin-binding protein malE | 0.25 | Stat | NS | NS |
| *M5005_spy1059* | *malF* | Maltose transport system permease protein malF | 0.21 | Stat | NS | 2.43 (S) |
| *M5005_spy1060* | *malG* | Maltose transport system permease protein malG | 0.41 | Stat | NS | 2.10 (S) |
| *M5005_spy1062* | *malA* | Maltodextrose utilization protein malA | 26.9/26.7 | Mid/Stat | 6.91/9.51 | 2.63 (S) |
| *M5005_spy1063* | *malD* | Maltodextrin transport system permease protein malD | 8.81/4.55 | Mid/Stat | 4.70/2.74 | NS |
| *M5005_spy1064* | *malC* | Maltose transport system permease protein malC | 6.08/3.90 | Mid/Stat | 5.86/2.34 | NS |
| *M5005_spy1065* | *amyA* | Alpha-amylase | 6.39/3.67 | Mid/Stat | 8.14/2.32 | NS |
| *M5005_spy1066* | *amyB* | Neopullulanase/cyclomaltodextrinase/maltogenic alpha-amylase | 10.0/5.91 | Mid/Stat | 5.38/3.13 | NS |
| *M5005_spy1067* | *malX* | Maltose/maltodextrin-binding protein malX | 9.14/4.09 | Mid/Stat | 6.80/2.14 | NS |
| *M5005_spy1079* |  | PTS system, cellobiose-specific IIC component | 17.2/11.4 | Mid/Stat | 8.87/3.11 | NS |
| *M5005_spy1081* |  | PTS system, cellobiose-specific IIA component | 8.97/5.64 | Mid/Stat | 7.61/1.84 | NS |
| *M5005_spy1082* |  | PTS system, cellobiose-specific IIB component | 13.1/6.71 | Mid/Stat | 8.88 (M) | NS |
| *M5005_spy1083* |  | Transcription antiterminator, BglG family/PTS system, mannitol (cryptic)-specific IIA component | 28.6/5.10 | Mid/Stat | 7.67/2.49 | 3.36/2.11 |
| *M5005_spy1085* | *bglA.2* | Beta-glucosidase | 5.20/4.05 | Mid/Stat | NS | NS |
| *M5005_spy1139* | *nagB* | Glucosamine-6-phosphate isomerase | 2.04 | Mid | NS | NS |
| *M5005_spy1235* | *N/A* | Phosphoglucomutase/phosphomannomutase | 2.77 | Mid | NS | NS |
| *M5005_spy1257* | *glcK* | Glucokinase/xylose represssor | 2.74 | Stat | NS | NS |
| *M5005_spy1304* | *lacZ* | Beta-galactosidase/Beta-glucosidase | 3.25/2.70 | Mid/Stat | NS | NS |
| *M5005_spy1308* |  | Sugar-binding protein | 3.82/3.01 | Mid/Stat | 2.06 (M) | NS |
| *M5005_spy1309* |  | Sugar transport system permease protein | 9.12/4.55 | Mid/Stat | 2.92 (S) | 5.85 (S) |
| *M5005_spy1310* |  | Sugar transport system permease protein | 3.15/2.39 | Mid/Stat | NS | NS |
| *M5005_spy1375* | *tkt* | Transketolase | 2.86/2.15 | Mid | 2.34 (M) | NS |
| *M5005_spy1376* |  | Transaldolase | 6.13/5.61 | Mid/Stat | 4.20/2.77 | NS |
| *M5005_spy1379* | *glpF* | Glycerol uptake facilitator protein | 21.2/9.56 | Mid/Stat | 11.2/2.84 | NS |
| *M5005_spy1395* | *lacD.1* | Tagatose-bisphosphate aldolase | 17.1/7.83 | Mid/Stat | 5.39 (M) | NS |
| *M5005_spy1396* | *nadE* | Tagatose-6-phosphate kinase | 9.02/6.69 | Mid/Stat | 6.23 (M) | NS |
| *M5005_spy1397* | *lacB.1* | Galactose-6-phosphate isomerase lacB subunit | 20.8/10.2 | Mid/Stat | 9.12 (M) | NS |
| *M5005_spy1398* | *lacA.1* | Galactose-6-phosphate isomerase lacA subunit | 56.8/22.5 | Mid/Stat | 7.21/2.80 | 2.27 (S) |
| *M5005_spy1399* |  | PTS system, galactose-specific IIC component | 16.5/3.35 | Mid/Stat | 5.96 (M) | NS |
| *M5005_spy1400* |  | PTS system, galactose-specific IIB component | 10.1/2.64 | Mid/Stat | 8.25 (M) | NS |
| *M5005_spy1401* |  | PTS system, galactose-specific IIA component | 20.2/2.18 | Mid/Stat | 7.13 (M) | NS |
| *M5005_spy1479* | *manL* | PTS system, mannose-specific IIAB component | 2.01 | Stat | NS | NS |
| *M5005_spy1480* | *manM* | PTS system, mannose-specific IIC component | 2.16 | Stat | NS | NS |
| *M5005_spy1481* | *manN* | PTS system, mannose-specific IID component | 2.36/2.13 | Mid/Stat | NS | NS |
| *M5005_spy1538* | *pmi* | Mannonse-6-phosphate isomerase | 24.0 | Stat | 9.02 (S) | 3.03 (S) |
| *M5005_spy1539* | *scrK* | Fructokinase | 35.6 (S) | Stat | 25.0 (S) | NS |
| *M5005_spy1542* | *scrA* | PTS system, sucrose-specific IIABC component | 30.3 | Stat | 24.2 (S) | 3.23 (S) |
| *M5005_spy1543* | *scrB* | Sucrose-6-phosphate hydrolase | 8.9 | Stat | 9.02 | NS |
| *M5005_spy1632* | *lacG* | 6-phospho-beta-glucosidase | 28.8 | Stat | -3.21/8.91 | NS |
| *M5005_spy1633* | *lacE* | PTS system, lactose-specific IIBC component | 16.8 | Stat | -2.52/7.52 | NS |
| *M5005_spy1634* | *lacF* | PTS system, lactose-specific IIA component | 13.8 | Stat | -2.71/6.39 | NS |
| *M5005_spy1635* | *lacD.2* | Tagatose-bisphosphate aldolase | 10.6 | Stat | -1.91/5.73 | NS |
| *M5005_spy1636* | *lacC.2* | Tagatose-6-phosphate kinase | 14.3 | Stat | -2.03/8.21 | NS |
| *M5005_spy1637* | *lacB.2* | Galactose-6-phosphate isomerase lacB subunit | 13.9 | Stat | -3.22/7.56 | NS |
| *M5005_spy1638* | *lacA.2* | Galactose-6-phosphate isomerase lacA subunit | 10.3 | Stat | -2.97/6.39 | NS |
| *M5005_spy1661* |  | Transaldolase | 8.94/6.55 | Mid/Stat | 3.35 (M) | NS |
| *M5005_spy1662* | *ulaA* | Ascorbate-specific PTS system enzyme IIC | 12.4/5.71 | Mid/Stat | 5.78/2.91 | 2.01 (M) |
| *M5005_spy1663* |  | PTS system, IIB component | 23.8/18.4 | Mid/Stat | 5.89/4.86 | NS |
| *M5005_spy1664* |  | PTS system, mannitol (cryptic)-specific IIA component | 5.09/3.09 | Mid/Stat | 3.10 (M) | NS |
| *M5005_spy1682* | *msmK* | Multiple sugar transport ATP-binding protein | 2.10 | Mid | NS | NS |
| *M5005_spy1692* |  | PTS system, glucose-specific IIABC component | 2.32 | Mid | NS | NS |
| *M5005_spy1693* |  | PTS system, glucose-specific IIABC component | 2.35 | Mid | NS | NS |
| *M5005_spy1744* |  | PTS system, cellobiose-specific IIC component | 6.22/5.46 | Mid/Stat | 2.07/3.31 | NS |
| *M5005_spy1745* |  | PTS system, cellobiose-specific IIB component | 15.9/6.45 | Mid/Stat | 4.31/3.56 | NS |
| *M5005_spy1746* |  | PTS system, cellobiose-specific IIA component | 10.7/4.69 | Mid/Stat | 3.03/2.00 | NS |
| *M5005_spy1783* | *dexS* | Trehalose-6-phosphate hydrolase | 2.93/1.27 | Mid/Stat | NS | 3.21/2.84 |
| *M5005_spy1784* |  | PTS system, trehalose-specific IIBC component | 3.42 | Mid | NS | 2.29/4.25 |
| **Cell motility** | | |  |  |  |  |
| *M5005_spy1007* |  | Phage protein | 2.55/2.55 | Mid/Stat | NS | NS |
| **Cellular processing** | | |  |  |  |  |
| *M5005_spy0835* |  | Class B acid phosphatase | 5.49/4.78 | Mid/Stat | 2.74 (M) | NS |
| *M5005_spy0836* |  | Acid phosphatase/phosphotransferase | 7.01/4.11 | Mid/Stat | 3.10 (M) | NS |
| **Cell wall/membrane biogenesis** | | |  |  |  |  |
| *M5005_spy0500* |  | N-acetylmuramoyl-L-alanine amidase | 2.33/3.30 | Mid/Stat | NS | NS |
| *M5005_spy0598* | *mscL* | Large-conductance mechanosensitive channel | 2.44 | Mid | NS | NS |
| *M5005_spy1843* |  | Transglycosylase SLT domain-containing protein | 5.28/6.88 | Mid/Stat | 5.54 (S) | NS |
| *M5005_spy1851* | *hasA* | Hyaluronan synthase | 25.2/73.0 | Mid/Stat | NS | 41.3/49.6 |
| *M5005_spy1852* | *hasB* | UDP-glucose 6-dehydrogenase | 17.7/47.9 | Mid/Stat | NS | 32.8/36.6 |
| *M5005_spy1853* | *hasC* | UTP-glucose-1-phosphate uridylyltransferase | 16.3/30.7 | Mid/Stat | NS | 29.0/24.5 |
| **Coenzyme and cofactor metabolism** | | |  |  |  |  |
| M5005_spy0898 |  | 2-(5’’-triphosphoribosyl)-3’-dephosphocoenzyme-A synthase | 0.47 | Stat | NS | NS |
| *M5005_spy0908* | *citX* | Apo-citrate lyase phosphoribosyl-dephospho-CoA transferase | 5.96/3.40 | Mid/Stat | 5.90 (M) | NS |
| *M5005_spy0945* | *coaA* | Pantothenate kinase | 2.36/8.01 | Mid/Stat | NS | NS |
| *M5005_spy1357* | *nadE* | NAD synthatase | -5.41 | Mid | NS | NS |
| **Defense mechanisms/virulence** | | |  |  |  |  |
| *M5005_spy0041* |  | Na+ driven multidrug efflux pump | 2.12 | Mid | NS | NS |
| *M5005_spy0042* |  | Na+ driven multidrug efflux pump | 2.09 | Mid | NS | NS |
| *M5005_spy0139* | *nga* | NAD glycohydrolase | 31.2/28.6 | Mid/Stat | 3.56 (S) | 12.1/21.3 |
| *M5005_spy0141* | *slo* | Streptolysin O | 25.3/32.5 | Mid/Stat | 2.73 (S) | 12.3/25.2 |
| *M5005_spy0341* | *spyCEP* | IL-8-degrading proteinase | 25.5/34.2 | Mid/Stat | 8.33/7.43 | 17.4/10.1 |
| *M5005_spy0351* | *spyA* | ADP-ribosyltransferase, C3 family | 5.2/10.3 | Mid/Stat | NS | 3.78/14.7 |
| *M5005_spy0356* | *speJ* | Exotoxin type J precursor | 2.2/5.1 | Mid/Stat | NS | 2.77/3.14 |
| *M5005_spy0561* | *epf* | Putative extracellular matrix binding protein | 17.0/11.1 | Mid/Stat | NS | 14.7/15.3 |
| *M5005_spy0562* | *sagA* | Streptolysin S precursor | 8.35/8.36 | Mid/Stat | 2.68 (M) | 2.31/5.34 |
| *M5005_spy0563* | *sagB* | Streptolysin S biosynthesis protein sagB | 10.5/19.2 | Mid/Stat | 3.65/5.28 | 3.21/15.5 |
| *M5005_spy0564* | *sagC* | Streptolysin S biosynthesis protein sagC | 8.84/15.3 | Mid/Stat | 3.12/4.12 | 2.81/8.71 |
| *M5005_spy0565* | *sagD* | Streptolysin S biosynthesis protein sagD | 9.79/12.5 | Mid/Stat | 3.82/4.60 | 4.02/10.0 |
| *M5005_spy0566* | *sagE* | Streptolysin S putative self-immunity protein sagE | 10.4/19.3 | Mid/Stat | 3.73/5.84 | 3.01/14.9 |
| *M5005_spy0567* | *sagF* | Streptolysin S biosynthesis protein sagF | 6.15/9.57 | Mid/Stat | 3.14/4.65 | 2.56/11.2 |
| *M5005_spy0568* | *sagG* | Streptolysin S export ATP-binding protein sagG | 5.32/8.30 | Mid/Stat | NS | 2.07/6.57 |
| *M5005_spy0569* | *sagH* | Streptolysin S export transmembrane protein sagH | 6.50/10.1 | Mid/Stat | 3.03/3.93 | 2.38/8.12 |
| *M5005_spy0570* | *sagI* | Streptolysin S export transmembrane protein sagI | 5.22/6.94 | Mid/Stat | 2.23/3.01 | 2.15/5.51 |
| *M5005_spy0571* |  | Endonuclease/exonuclease/phosphatase family protein | 3.89/3.87 | Mid/Stat | NS | NS |
| *M5005_spy0667* |  | Exotoxin type C precursor | 40.2/83.4 | Mid/Stat | NS | 41.6/75.9 |
| *M5005_spy0668* | *mac* | IgG-degrading protease of GAS | 30.8/46.4 | Mid/Stat | NS | 38.8/50.2 |
| *M5005_spy0803* | *srtI* | Protein involved in lantibiotic (srt) production | 3.16 | Mid | NS | 2.71/2.11 |
| *M5005_spy0996* | *speA2* | Exotoxin type A precursor, A2 allele | 4.14/8.65 | Mid/Stat | NS | 7.27/19.5 |
| *M5005_spy1012* |  | Antigen A | 2.52/2.10 | Mid/Stat | NS | NS |
| *M5005_spy1013* |  | Antigen B | 2.57/3.13 | Mid/Stat | NS | NS |
| *M5005_spy1106* | *grab* | Protein G related α-2M binding protein | 2.32 | Mid | NS | 3.57 (M) |
| *M5005_spy1415* | *sdaD2* | Streptodornase | 2.2/10.1 | Mid/Stat | 2.31 (M) | 2.69/7.86 |
| *M5005_spy1540* | *endoS* | Endo-beta-N-acetylglucosaminidase F2 precursor | 26.6 (S) | Stat | 21.3 (S) | 2.61 (S) |
| *M5005_spy1684* | *ska* | Streptokinase | 2.34/17.6 | Mid/Stat | NS | 3.24/13.3 |
| *M5005_spy1687* | *sclA* | Collagen-like surface protein A | 33.9/41.1 | Mid/Stat | NS | 37.4/17.5 |
| *M5005_spy1688* |  | Immunoglobulin receptor precursor | 98.2/35.3 | Mid/Stat | NS | 2.29 (M) |
| *M5005_spy1689* |  | Collagen-like surface protein | 19.2/453 | Mid/Stat | NS | 21.4/196 |
| *M5005_spy1691* |  | Endonuclease/exonuclease/phosphatase family protein | 7.65 | Mid | 3.15/2.01 | 2.97/4.24 |
| *M5005_spy1714* | *fba* | Fibronectin binding protein | 5.01 | Stat | 3.23 (S) | 4.12 (S) |
| *M5005_spy1715* | *scpA* | C5a peptidase precursor protein | 5.23 | Stat | 2.23 (S) | 5.13 (S) |
| *M5005_spy1718* | *sic* | Streptococcal inhibitor of complement | 4.23 | Stat | NS | 2.39 (S) |
| *M5005_spy1735* | *speB* | Cysteine protease | -5.23 | Mid | -5.21 (M) | 2.23 (M) |
| *M5005_spy1738* | *sda* | Streptodornase | 3.02 | Mid | NS | 2.24 (M) |
| **Energy production and conversion** | | |  |  |  |  |
| *M5005_spy0039* | *adh2* | Alcohol/acetaldehyde-CoA dehydrogenase | 11.0 | Mid | NS | 2.60/2.23 |
| *M5005_spy0040* | *adhA* | Alcohol dehydrogenase | 12.1/4.14 | Mid/Stat | 5.91/2.52 | NS |
| *M5005_spy0126* | *ntpI* | V-type sodium ATP synthase subunit I | 4.32/29.2 | Mid/Stat | 12.3/0.18 | NS |
| *M5005_spy0127* | *ntpK* | V-type sodium ATP synthase subunit K | 3.58/20.9 | Mid/Stat | 11.3/3.11 | NS |
| *M5005_spy0128* | *ntpE* | V-type sodium ATP synthase subunit E | 4.21/17.0 | Mid/Stat | 11.0/3.61 | NS |
| *M5005_spy0129* | *ntpC* | V-type ATP synthase subunit C | 7.92/26.5 | Mid/Stat | 18.9/4.09 | NS |
| *M5005_spy0130* | *ntpF* | V-type ATP synthase subunit F | 3.92/13.6 | Mid/Stat | 11.8/3.11 | NS |
| *M5005_spy0131* | *ntpA* | V-type sodium ATP synthase subunit A | 3.46/12.1 | Mid/Stat | 9.47/2.85 | NS |
| *M5005_spy0132* | *ntpB* | V-type sodium ATP synthase subunit B | 4.87/19.4 | Mid/Stat | 12.6/3.61 | NS |
| *M5005_spy0133* | *ntpD* | V-type sodium ATP synthase subunit D | 4.83/18.5 | Mid/Stat | 14.4/3.32 | NS |
| *M5005_spy0340* | *lctO* | L-lactate oxidase | 39.4/6.16 | Mid/Stat | 13.8/2.19 | NS |
| *M5005_spy0790* | *gabD* | Succinate-semialdehyde dehydrogenase [NADP+] | 2.96/3.88 | Mid/Stat | NS | NS |
| *M5005_spy0900* |  | Mg2+/citrate complex secondary transporter | 5.76/7.97 | Mid/Stat | NS | NS |
| *M5005_spy0903* | *oadB* | Oxaloacetate decarboxylase beta chain | 2.06 | Mid | NS | 2.70 (S) |
| *M5005_spy0905* | *citD* | Citrate lyase subunit gamma/acyl carrier protein | 2.44 | Mid | NS | NS |
| *M5005_spy0906* | *citE* | Citrate lyase beta chain/citryl-CoA lyase subunit | 2.29 | Mid | NS | 2.13 (S) |
| *M5005_spy0907* | *citF* | Citrate lyase alpha chain/citrate CoA-transferase | 2.52 | Mid | NS | 2.16 (S) |
| *M5005_spy0909* | *oadA* | Oxaloacetate decarboxylase alpha chain | 2.26/0.40 | Mid/Stat | NS | NS |
| *M5005_spy1380* | *glpO* | Alpha-glycerophosphate oxidase | 11.2/9.97 | Mid/Stat | 5.07/3.23 | NS |
| *M5005_spy1381* | *glpK* | Glycerol kinase | 8.27/7.06 | Mid/Stat | 2.63 (M) | NS |
| **Inorganic ion transport and metabolism** | | |  |  |  |  |
| *M5005_spy0543* | *adcA* | High-affinity zinc uptake system protein znuA precursor | 2.40 | Mid | NS | 2.28 (M) |
| *M5005_spy0786* |  | Iron(III)-binding protein | 2.33 | Stat | NS | NS |
| *M5005_spy0985* |  | PhnA protein | -3.52 | Mid | NS | NS |
| *M5005_spy1161* |  | Formate transporter | 3.16/2.71 | Mid/Stat | NS | NS |
| *M5005_spy1167* |  | Lead, cadmium, zinc and mercury transporting ATPase | 2.30 | Mid | NS | NS |
| *M5005_spy1403* |  | Copper chaperone | 3.24 | Mid | NS | 3.43/3.48 |
| *M5005_spy1711* | *lmb* | Laminin binding protein | 2.92 | Mid | NS | 2.00 (M) |
| **Intracellular trafficking and secretion** | | |  |  |  |  |
| *M5005_spy0664* | *mur1.2* | Autolysin | 2.37/2.38 | Mid/Stat | NS | NS |
| *M5005_spy1751* | *secE* | Protein translocase subunit secE | 2.19 | Mid | NS | NS |
| **Lipid transport and metabolism** | | |  |  |  |  |
| *M5005_spy0116* | *atoE* | Short-chain fatty acids transporter | 11.9 | Mid | NS | NS |
| *M5005_spy0119* |  | Acetyl-CoA acetyltransferase | 3.95/3.42 | Mid/Stat | NS | NS |
| *M5005_spy0120* | *atoD.2* | Acetate CoA-transferase alpha subunit | 2.87/6.96 | Mid/Stat | NS | NS |
| *M5005_spy0121* |  | Acetyl-CoA:acetoacetyl-CoA transferase beta subunit | 3.22/5.72 | Mid/Stat | NS | NS |
| *M5005_spy0359* | *fabG* | 3-ketoacyl-acyl carrier protein reductase | -2.16 | Stat | NS | -2.56 (S) |
| *M5005_spy0534* | *bsaA* | Acetoin (diacetyl) reductase | 3.31 | Mid | NS | NS |
| *M5005_spy0535* |  | Acetoin dehydrogenase | 5.12/1.68 | Mid/Stat | 2.30 (M) | NS |
| *M5005_spy0902* |  | Acetyl-CoA carboxylase biotin carboxyl carrier protein subunit | 2.48/0.08 | Mid/Stat | NS | 2.40 (S) |
| **Nucleotide transport and metabolism** | | |  |  |  |  |
| *M5005_spy0080* |  | Bis(5’-nucleosyl)-tetraphosphatase | 3.22 | Stat | 5.82 (S) | -4.90 (S) |
| *M5005_spy0639* | *pyrR* | Pyramidine regulatory protein | -5.82 | Stat | NS | -6.88 (S) |
| *M5005_spy0640* | *pyrP* | Uracil permease | -6.23 | Stat | NS | -8.72 (S) |
| *M5005_spy0641* | *pyrB* | Aspartate carbamoyl transferase | -6.71 | Stat | NS | -5.51 (S) |
| *M5005_spy0642* | *carA* | Carbamoyl phosphate synthase | -5.85 | Stat | NS | -5.69 (S) |
| *M5005_spy0643* | *carB* | Carbamoyl phosphate synthase | -4.82 | Stat | NS | -5.71 (S) |
| *M5005_spy0678* |  | 5’-nucleotidase | 4.89/8.65 | Mid/Stat | 2.25/4.59 | NS |
| *M5005_spy0775* |  | Nucleoside diphosphate kinase | 2.70 | Mid | NS | 2.02 (M) |
| *M5005_spy0857* | *guaC* | GMP reductase | 2.16 | Mid | NS | NS |
| *M5005_spy0858* | *xpt* | Xanthine phosphoribosyl transferase | -10.3 | Mid | NS | NS |
| *M5005_spy0859* |  | Xanthine permease | -12.2 | Mid | NS | NS |
| *M5005_spy0939* |  | Nucleoside transport system permease protein | 2.16/2.98 | Mid/Stat | NS | NS |
| *M5005_spy1477* |  | Guanine/hypoxanthine permease | -8.7 | Mid | NS | NS |
| *M5005_spy1585* | *deoC* | Deoxyribose-phosphate aldolase | 2.00 | Mid | NS | NS |
| *M5005_spy1587* | *udp* | Uridine phosphorylase | 2.75 | Mid | NS | NS |
| **Phage** | |  |  |  |  |  |
| *M5005_spy0459* |  | Portal protein | 5.99 | Mid | 5.01 (M) | 2.26 (M) |
| *M5005_spy0995* |  | Phage protein | 8.54/11.9 | Mid/Stat | 2.73/4.82 | 2.51 (M) |
| *M5005_spy0997* |  | Phage protein | 3.78/2.20 | Mid/Stat | NS | NS |
| *M5005_spy0999* |  | Phage protein | 8.82/2.20 | Mid/Stat | 2.09 (M) | NS |
| *M5005_spy1000* |  | Phage protein | 2.92/4.64 | Mid/Stat | NS | NS |
| *M5005_spy1004* |  | Phage protein | 3.30/5.00 | Mid/Stat | NS | NS |
| *M5005_spy1005* |  | Phage protein | 2.12/2.08 | Mid/Stat | NS | NS |
| *M5005_spy1006* |  | Phage structural protein | 10.3/3.50 | Mid/Stat | NS | NS |
| *M5005_spy1009* |  | Phage protein | 4.50/2.04 | Mid/Stat | NS | NS |
| *M5005_spy1010* |  | Phage protein | 9.10/6.17 | Mid/Stat | NS | NS |
| *M5005_spy1011* |  | Phage protein | 4.82/6.78 | Mid/Stat | NS | NS |
| *M5005_spy1017* |  | Phage protein | 2.56/3.21 | Mid/Stat | NS | NS |
| *M5005_spy1018* |  | Phage protein | 2.32 | Mid | NS | NS |
| *M5005_spy1019* |  | Phage scaffold protein | 8.21/11.6 | Mid/Stat | NS | NS |
| *M5005_spy1020* |  | Phage protein | 2.84 | Mid | NS | NS |
| *M5005_spy1021* |  | Phage protein | 13.2/9.11 | Mid/Stat | 3.21 (S) | 4.53 (M) |
| *M5005_spy1022* |  | Portal protein | 2.93/2.42 | Mid/Stat | NS | 2.53 (M) |
| *M5005_spy1029* |  | Phage protein | 2.43/2.99 | Mid/Stat | NS | NS |
| *M5005_spy1038* |  | Phage protein | 2.12 | Mid | NS | NS |
| *M5005_spy1047* |  | Phage protein | 2.12/2.17 | Mid/Stat | NS | NS |
| *M5005_spy1049* |  | Phage protein | 10.8 | Mid | NS | NS |
| *M5005_spy1172* |  | Holin | 2.90 | Mid | 2.35 (M) | NS |
| *M5005_spy1173* |  | Phage protein | 5.08 | Mid | 2.88 (M) | 5.35 (M) |
| *M5005_spy1175* |  | Phage protein | 12.1 | Mid | NS | 3.40 (M) |
| *M5005_spy1176* |  | Phage infection protein | 2.08/5.87 | Mid/Stat | 4.49/3.29 | NS |
| *M5005_spy1201* |  | Phage protein | 2.75 | Mid | 3.81 (M) | NS |
| *M5005_spy1203* |  | Phage protein | 5.12/5.33 | Mid/Stat | NS | NS |
| *M5005_spy1429* |  | Phage protein | -2.19 | Mid | NS | -2.28/-4.08 |
| **Post-translational modification, protein turnover, chaperones** | | |  |  |  |  |
| *M5005_spy1080* |  | Hypothetical protein | 8.76/7.30 | Mid/Stat | 2.39/2.45 | NS |
| *M5005_spy1282* | *msrA* | Bifunctional methionine sulfoxide reductase A/B peptide | 3.44 | Mid | 3.44 (M) | NS |
| **Replication, recombination and repair** | | |  |  |  |  |
| *M5005_spy0113* |  | Transposase | 18.0/11.7 | Mid/Stat | NS | 97.5/11.5 |
| *M5005_spy0254* |  | Transposase | 6.48/4.07 | Mid/Stat | 4.70/2.32 | 4.50 (M) |
| *M5005_spy0800* |  | DNA-cytosine methyltransferase | 4.59/4.85 | Mid/Stat | NS | NS |
| *M5005_spy0840* | *radC* | DNA repair protein radC | 2.97 | Mid | NS | NS |
| *M5005_spy1043* |  | Phage protein | 2.76 | Mid | NS | NS |
| *M5005_spy1285* |  | Hypothetical protein | 4.40/6.31 | Mid/Stat | NS | 2.04/5.73 |
| *M5005_spy1286* |  | DNA polymerase | 3.43/4.84 | Mid/Stat | NS | NS |
| *M5005_spy1287* |  | Hypothetical protein | 4.22/6.93 | Mid/Stat | NS | 2.08/7.95 |
| *M5005_spy1643* |  | DNA integration/recombination/invertion protein | 2.48 | Mid | 2.42 (M) | NS |
| **Signal transduction mechanisms** | | |  |  |  |  |
| *M5005_spy1276* |  | Transcription regulator, Crp family | 2.80/2.35 | Mid/Stat | NS | NS |
| *M5005_spy1305* | *lytR* | Two-component response regulator, yesN | 3.11/2.12 | Mid/Stat | NS | NS |
| *M5005_spy1306* | *lytS* | Two-component sensor kinase, yesM | 3.19/2.07 | Mid/Stat | NS | NS |
| *M5005_spy1574* |  | Universal stress protein | -3.32 | Stat | NS | NS |
| **Stress** | |  |  |  |  |  |
| *M5005_spy1378* |  | NADH peroxidase | 13.2/8.21 | Mid/Stat | 6.47/3.08 | NS |
| **Transcription** | | |  |  |  |  |
| *M5005_spy0117* |  | Transcriptional regulators, LysR family | 14.1/16.5 | Mid/Stat | 4.91/4.27 | NS |
| *M5005_spy0118* |  | Transcriptional regulator, LysR family | 15.9/16.4 | Mid/Stat | 4.73/4.64 | NS |
| *M5005_spy0124* | *sloR* | Transcriptional regulator | -4.25 | Mid | NS | NS |
| *M5005_spy0474* | *licT* | Transcription antiterminator, BglG family | 17.0/30.6 | Mid/Stat | 6.41/12.2 | NS |
| *M5005_spy1045* |  | Transcriptional regulator | 2.02 | Mid | NS | NS |
| *M5005_spy1061* |  | Transcriptional regulator, LacI family | 0.37/0.27 | Mid/Stat | NS | NS |
| *M5005_spy1277* | *ahrC.2* | Arginine repressor, ArgR family | 3.01/3.13 | Mid/Stat | NS | NS |
| *M5005_spy1315* |  | Transcriptional regulator, GntR family | 2.04 | Mid | NS | NS |
| *M5005_spy1377* |  | Trans-acting positive regulator | 10.5/6.52 | Mid/Stat | 4.78/2.49 | NS |
| *M5005_spy1392* |  | Transcriptional regulator, TetR family | 2.16/2.21 | Mid/Stat | NS | NS |
| *M5005_spy1578* |  | Transcriptional regulator, Cro/CI family | 2.86 | Mid | NS | NS |
| *M5005_spy1668* |  | Putative transcriptional regulator | 3.60/4.00 | Mid/Stat | NS | NS |
| *M5005_spy1760* |  | Transcriptional regulator, MutR family | 39.6/4.59 | Mid/Stat | 8.39 (M) | 4.63 (M) |
| *M5005_spy1779* |  | Transcriptional regulator, LuxR family | 23.1/16.8 | Mid/Stat | 11.8/7.66 | NS |
| *M5005_spy1825* |  | Transcriptional regulator, PhdR family | -8.76 | Stat | NS | NS |
| **Translation** | |  |  |  |  |  |
| *M5005_spy0123* |  | Translation initiation inhibitor | -4.25 | Mid | NS | NS |
| *M5005_spy0798* |  | IFN-response binding factor 1 | 4.30 | Mid | 3.38 (M) | 5.92 (M) |
| **Unknown** | |  |  |  |  |  |
| *M5005_spy0015* |  | Hypothetical protein | 14.2/2.18 | Mid/Stat | NS | 5.28 (M) |
| *M5005_spy0098* |  | Hypothetical protein | 2.14 | Mid | NS | NS |
| *M5005_spy0115* |  | Hypothetical protein | 48.3/265 | Mid/Stat | NS | 63.0/300 |
| *M5005_spy0125* |  | Hypothetical protein | 4.47/30.7 | Mid/Stat | 12.5/4.29 | NS |
| *M5005_spy0142* |  | Hypothetical protein | 3.22/9.28 | Mid/Stat | NS | 313/165 |
| *M5005_spy0143* |  | Hypothetical protein | 122/112 | Mid/Stat | NS | 171.94.5 |
| *M5005_spy0144* |  | Hypothetical protein | 382/351 | Mid/Stat | NS | NS |
| *M5005_spy0177* |  | BioY protein | 2.19/2.79 | Mid/Stat | NS | NS |
| *M5005_spy0281* |  | Hypothetical cytosolic protein | 2.33/4.36 | Mid/Stat | NS | 2.78/3.77 |
| *M5005_spy0352* |  | Hypothetical membrane associated protein | 31.7/69.0 | Mid/Stat | NS | 30.4/45.0 |
| *M5005_spy0353* |  | Hypothetical membrane spanning protein | 4.24/16.8 | Mid/Stat | NS | 2.68/8.17 |
| *M5005_spy0354* |  | Hypothetical protein | 6.30/10.6 | Mid/Stat | NS | 11.2/7.54 |
| *M5005_spy0355* |  | Hypothetical protein | 16.8/128 | Mid/Stat | NS | 38.4/81.8 |
| *M5005_spy0357* |  | Hypothetical protein | 3.14/5.05 | Mid/Stat | NS | 5.12/2.69 |
| *M5005_spy0360* |  | NAD-dependent oxidoreductase | -2.28 | Stat | NS | -2.56 (S) |
| *M5005_spy0404* |  | Hypothetical protein | 2.35 | Mid | NS | NS |
| *M5005_spy0518* |  | Oligohyaluronate lyase | 5.71/11.3 | Mid/Stat | 2.21/2.12 | 2.41/6.17 |
| *M5005_spy0666* |  | Hypothetical protein | 47.3/33.5 | Mid/Stat | NS | 49.3/36.3 |
| *M5005_spy0742* |  | Hypothetical protein | 2.64/2.19 | Mid/Stat | NS | NS |
| *M5005_spy0773* |  | Hypothetical protein | 14.8/4.99 | Mid/Stat | 5.63 (M) | 7.48/3.49 |
| *M5005_spy0812* |  | Hypothetical protein | 5.09 | Mid | 4.29 (M) | 3.35 (M) |
| *M5005_spy0852* |  | Short chain dehydrogenase | 2.71 | Mid | NS | NS |
| *M5005_spy0853* |  | Short chain dehydrogenase | 2.75 | Mid | NS | NS |
| *M5005_spy0880* |  | Hypothetical protein | -3.56 | Stat | NS | NS |
| *M5005_spy0979* |  | Hypothetical protein | 2.36 | Mid | NS | NS |
| *M5005_spy0981* | *cfa* | cAMP factor | 4.33 | Mid | NS | NS |
| *M5005_spy1001* |  | Phage-associated cell wall hydrolase | 2.36/2.60 | Mid/Stat | NS | NS |
| *M5005_spy1008* |  | Hypothetical protein | 4.21/3.27 | Mid/Stat | NS | NS |
| *M5005_spy1023* |  | Terminase large subunit | 4.38 | Mid | NS | NS |
| *M5005_spy1078* |  | Hypothetical protein | 6.58/3.66 | Mid/Stat | 3.66/2.17 | NS |
| *M5005_spy1084* |  | Outer surface protein | 53.9/41.8 | Mid/Stat | 4.35/3.31 | NS |
| *M5005_spy1093* |  | Hypothetical protein | 29.1/6.00 | Mid/Stat | 7.49 (M) | NS |
| *M5005_spy1142* |  | Hypothetical protein | 18.4/5.21 | Mid/Stat | NS | 16.1/3.97 |
| *M5005_spy1143* |  | Hypothetical protein | 6.57/4.15 | Mid/Stat | NS | 6.56/2.40 |
| *M5005_spy1144* |  | Hypothetical protein | 3.53/3.09 | Mid/Stat | NS | 3.97/2.44 |
| *M5005_spy1289* |  | Hypothetical protein | 3.64/6.20 | Mid/Stat | NS | NS |
| *M5005_spy1290* |  | Hypothetical protein | 4.96/9.93 | Mid/Stat | NS | 2.11/15.7 |
| *M5005_spy1307* |  | Hypothetical protein | 4.29/2.61 | Mid/Stat | NS | NS |
| *M5005_spy1541* |  | Hypothetical protein | 33.2 | Stat | 27.3 (S) | 3.05 (S) |
| *M5005_spy1556* |  | Hypothetical protein | 4.41/7.24 | Mid/Stat | NS | 5.12/8.80 |
| *M5005_spy1667* |  | Hypothetical protein | 4.07 | Mid | 3.50 (M) | 3.22 (M) |
| *M5005_spy1703* |  | Hypothetical cytosolic protein | 8.10 | Mid | 4.41 (M) | 6.81 (M) |
| *M5005_spy1731* |  | Hypothetical cytosolic protein | 4.16/38.8 | Mid/Stat | NS | 3.72/4.18 |

^1^positive numbers indicated increased transcript levels in mutant strain, whereas negative numbers indicate higher transcript levels in wild-type strain

^2^NS = no significant difference in transcript level between wild-type and isogenic mutant strain. If 2 numbers are listed then transcript levels were significantly different in both the mid-exponential and stationary growth phases. If 1 number is listed then the transcript level was only significantly different at one growth phase which is indicated by (M) for mid-exponential and (S) for stationary.
