# Supplementary material for: A Combination of Independent Transcriptional Regulators Shapes Bacterial Virulence Gene Expression during Infection
Source: PLoS Pathog. 2010 Mar 19;6(3):e1000817. doi: 10.1371/journal.ppat.1000817 (PMC2841617; doi:10.1371/journal.ppat.1000817)
Supplement: Table S5 — Primers and probes used in this study (0.09 MB DOC) [file ppat.1000817.s011.doc]

**Table S5 Primers and probes used in this study**.

| Primer | Sequence (5’→3’) | Target |
| --- | --- | --- |
| ccpA-A | GGG ATT GGC TGC ATT ATT TC | 5’ primer for 5’ region of *ccpA* |
| ccpA-B | GTT ATA GTT ATT ATA ACA TGT ATT CTT CAC GGG CAA CAT CAT AA | 3’ primer for 5’ region of *ccpA* with *spc* sequence overlap |
| ccpA-C | CTA TTT AAA TAA CAG ATT AAA AAA ATT ATA AAA ACG TGG GAC AAC TAA GTA ACT G | 5’ primer for 3’ region of *ccpA* with *spc* sequence overlap |
| ccpA-D | GAT TTT TCG GGT GGT TAC CA | 3’ primer for 3’ region of *ccpA* |
| ccpA-spcF | TTA TGA TGT TGC CCG TGA AGA ATA CAT GTT ATA ATA ACT ATA AC | 5’ primer for *spc* along with *ccpA* overlap sequence |
| ccpA-spcR | CAG TTA CTT AGT TGT CCC ACG TTT TTA TAA TTT TTT TAA TCT GTT ATT TAA ATA G | 3’ primer for *spc* along with *ccpA* overlap sequence |
| ccpA-SouthF | AAC ATG CTA GCA ATG CCT TC | 5’ *ccpA* primer for Southern blot |
| ccpA-SouthR | ACC AGT GAT ATG ACC AGA ACT GTG | 3’ *ccpA* primer for Southern blot |
| ccpA-*Xma*I-comp | ATA TCC CGG GCA AGA TAC AAA CGT ATT TG | 5’ *ccpA* primer for complementation |
| ccpA-*Eco*RI-comp | CGA GGA ATT CAG ATG GTG CTC ATA ATT C | 3’ *ccpA* primer for complementation |
| ccpA-prot-NcoI | ACC ATG GGT AAT ACA GAT GAT ACC ATT AC | 5’ *ccpA* primer for protein expression |
| ccpA-prot-EcoRI | CGA ATT CTT ACT TAG TTG TCC CAC GTT T | 3’ *ccpA* primer for protein expression |
| *ccpA*-QRT-5’ | GCG CAG CGC GTC ATT AA | 5’ *ccpA* primer for QRT-PCR |
| *ccpA*-QRT-3’ | AGC CAC ATA AGC CGC TGT TGC TCC T | 3’ *ccpA* primer for QRT-PCR |
| *ccpA*-QRT-probe | AGC CAC ATA AGC CGC TGT TGC TCC T | *ccpA* probe for QRT-PCR |
| HPr-Prot-NcoI | ACC ATG GGT GCT TCA AAA GAC TTT C | 5’ *hpr* primer for protein expression |
| HPr-Prot-EcoRI | CGA ATT CTT ATG CCA ATC CCT CTT TAG | 3’ *hpr* primer for protein expression |
| HPrK-Prot-NheI | GGA ATT CCA TAT GAG TAT TTG CAA GGG TCT AAC G | 5’ *hprK* primer for protein expression |
| HPrK-Prot-XhoI | CCG CTC GAG TCA TTG ACT CAC CTC ATT TTT GG | 3’ *hprK* primer for protein expression |
| CovR-Prot-NheI | GGT GGT CAT ATG ACA AAG AAA ATT TTA ATT | 5’ *covR* primer for protein expression |
| CovR-Prot-SapI | GGT GGT GCT CTT CCG CAT TTC TCA CGA ATA ACT T | 3’ *covR* primer for protein expression |
| *covR*-QRT-5’ | ATG TCT ATA TTC GCT ATC TCC GCG GCA AA | 5’ primer for *covR* QRT-PCR |
| *covR*-QRT-3’ | TGA TGA AGC CGT TGA GAC TAA TG | 3’ primer for *covR* QRT-PCR |
| *covR*-QRT-probe | GGA TAT AAG ATT CCT TGC CTG GAA | Probe for *covR* QRT-PCR |
| 5' *covRS* PCR | GAC CAT AGA GGG CAG AGA AGG | 5’ primer for PCR for *covRS* operon sequencing |
| 3’ *covRS* PCR | CAG TTG TGG CAA AAG ACT GC | 3’ primer for PCR for *covRS* operon sequencing |
| *covRS* seq1 | AAA ATT TGT GGG CTA TGT TCA AGT | *covRS* sequencing primer |
| *covRS* seq2 | GTC AGA AAA TCC AAA AAC GAT AGC | *covRS* sequencing primer |
| *covRS* seq3 | AAT ATC ACT GTC ATC TTT ACC CCA A | *covRS* sequencing primer |
| *covRS* seq4 | TAG AGT ATT TGT GAT ATC GCG CTC | *covRS* sequencing primer |
| *slo* QRT 5’ | GAAATATCCGTATCATGGCTAGAGAGT | 5’ primer for *slo* QRT-PCR |
| *slo* QRT 3’ | CACATCTCTTTCGTCGATCACTTT | 3’ primer for *slo* QRT-PCR |
| *slo* QRT probe | GCCACCATTCCCAAGCTAAGCCAGTG | Probe for *slo* QRT-PCR |
| *spyCEP* QRT 5’ | AAG GAG CTT GGG ACA AGG GAT A | 5’ primer for *spyCEP* QRT-PCR |
| *spyCEP* QRT 3’ | TGA TGG GCC GGA TCG A | 3’ primer for *spyCEP* QRT-PCR |
| *spyCEP* probe | AAG GAG CTT GGG ACA AGG GAT A | Probe for *spyCEP* QRT-PCR |
| *sagA* QRT 5’ | TTG CTC CTG GAG GCT GCT | 5’ primer for *sagA* QRT-PCR |
| *sagA* QRT 3’ | CTT CCG CTA CCA CCT TGA GAA T | 3’ primer for *sagA* QRT-PCR |
| *sagA* probe | ACC ACT TCC AGT AGC AAT TGA GAA GCA ACA AG | Probe for *sagA* QRT-PCR |
| *speB* QRT 5’ | CGC ACT AAA CCC TTC AGC TCT T | 5’ primer for *speB* QRT-PCR |
| *speB* QRT 3’ | ACA GCA CTT TGG TAA CCG TTG A | 3’ primer for *speB* QRT-PCR |
| *speB* probe | GCC TGC GCC GCC ACC AGT A | Probe for *speB* QRT-PCR |
| *hasA* QRT 5’ | ACC GTT CCC TTG TCA ATA AAG G | 5’ primer for *hasA* QRT-PCR |
| *hasA* QRT 3’ | CGT CAG CGT CAG ATC TTT CAA A | 3’ primer for *hasA* QRT-PCR |
| *hasA* probe | CGC CAT GCT CAA GCG TGG GC | Probe for *hasA* QRT-PCR |
| *speB* *cre* | AAA GAT AGC GCT ATC ACA CCC CCT GTG ATA GCG CTA TC TTT | Fluorescein tagged DNA from *speB* early intragenic region for CcpA DNA protein binding assay |
| *nga/slo* *cre* | GTT GAA ATC GAT TAC TAC ACC CCC TGT AGT AAT CGA TTT CAA C | Fluorescein tagged DNA from *nga/slo* promoter region for CcpA DNA protein binding assay |
| *spyCEP cre* | TAG GAA GCG TTT TCT ACC CCC TAG GAA AAC GCT TCC TA | Fluorescein tagged DNA from *spyCEP* promoter region for CcpA DNA protein binding assay |
| *arcA cre* | ATG TAA GCG ATT ACT CAA CCC CC TTG AGT AAT CGC TTA CAT | Fluorescein tagged DNA from *arcA* promoter region for CcpA DNA protein binding assay |
| *amyA cre* | ATG AAA GCG ATT ACG ATC CCC CAT CGT AAT CGC TTT CAT | Fluorescein tagged DNA from *amyA* promoter region for CcpA DNA protein binding assay |
| *ftsX* promoter probe | TCG TGT TGT TGC TAT TGA CCC CCT CAA TAG CAA CAA CAC GA | Fluorescein tagged DNA from *ftsX* promoter region for CcpA DNA protein binding assay |
| *hasA* promoter probe | CCT TAT TTT TAA AAA AAC TTC CCC CAA GTT TTT TTA AAA ATA AGG | Fluorescein tagged DNA from *hasA* promoter region for CovR DNA protein binding assay |
| *typA* promoter probe | AAG GCA GTC ACT GTC TCA AAC CCC CTT TGA GAC AGT GAC TGC CTT | Flourescein tagged DNA from *typA* promoter region for CovR DNA protein binding assay |
| *arcA* promoter probe | ACA AAA ACC AAT TAG AAA GAC CCC CTC TTT CTA ATT GGT TTT TGT | Flourescein tagged DNA from *arcA* promoter region for CovR DNA protein binding assay |
| *amyA* promoter probe | CAA CCA AAC TCT CAT TAG AAA CCC CCC TTT CTA ATG AGA GTT TGG TTG | Flourescein tagged DNA from *amyA* promoter region for CovR DNA protein binding assay |
| 5’ *sagA* promoter probe | TTT TGT CAT TTT TGA TAA TAT TAA AAA GAA AGG GTT TAC ATA TTA ATC AT | 5’-fluorescein tagged DNA from *sagA* promoter region for CovR DNA protein binding assay |
| 3’ *sagA* promoter probe | ATG ATT AAT ATG TAA ACC CTT TCT TTT TAA TAT TAT CAA AAA TGA CAA AA | 3’ DNA from *sagA* promoter region for CovR DNA protein binding assay |
